# miRNA Expression Profile Analysis in Kidney of Different Porcine Breeds

**DOI:** 10.1371/journal.pone.0055402

**Published:** 2013-01-25

**Authors:** Oriol Timoneda, Ingrid Balcells, Jose Ignacio Núñez, Raquel Egea, Gonzalo Vera, Anna Castelló, Anna Tomàs, Armand Sánchez

**Affiliations:** 1 Departament de Genètica Animal, Centre de Recerca en AgriGenòmica (CRAG), Universitat Autònoma de Barcelona, Campus UAB, Bellaterra, Spain; 2 Centre de Recerca en Sanitat Animal (CReSA), UAB, Bellaterra, Spain; 3 Program Infection and Immunity, FISIB, Bunyola, Spain; University of Iowa, United States of America

## Abstract

microRNAs (miRNAs) are important post-transcriptional regulators in eukaryotes that target mRNAs repressing their expression. The uncertain process of pig domestication, with different origin focuses, and the selection process that commercial breeds suffered, have generated a wide spectrum of breeds with clear genetic and phenotypic variability. The aim of this work was to define the miRNAs expression profile in kidney of several porcine breeds. Small RNA libraries from kidney were elaborated and high-throughput sequenced with the 454 Genome Sequencer FLX (Roche). Pigs used were classified into three groups: the European origin group (Iberian breed and European Wild Boar ancestor), European commercial breeds (Landrace, Large White and Piétrain breeds) and breeds with Asian origin (Meishan and Vietnamese breeds). A total of 229 miRNAs were described in the pig kidney miRNA profile, including 110 miRNAs out of the 257 previously described pig miRNAs and 119 orthologous miRNAs. The most expressed miRNAs in pig kidney microRNAome were Hsa-miR-200b-3p, Ssc-miR-125b and Ssc-miR-23b. Moreover, 5 novel porcine miRNAs and 3 orthologous miRNAs could be validated through RT-qPCR. miRNA sequence variation was determined in 116 miRNAs, evidencing the presence of isomiRs. 125 miRNAs were differentially expressed between breed groups. The identification of breed-specific miRNAs, which could be potentially associated to certain phenotypes, is becoming a new tool for the study of the genetic variability underlying complex traits and furthermore, it adds a new layer of complexity to the interesting process of pig evolution.

## Introduction

MicroRNAs (miRNAs) are small regulatory RNAs that play important roles in the regulation of gene expression [1], [2]. These single-stranded non-coding RNAs that are approximately 22 nucleotides long are involved in post-transcriptional regulation mechanisms acting mainly through down-regulation of target messenger RNAs (mRNAs) in a wide range of biological and pathological processes [3], [4]. Thus, miRNAs inhibit gene expression by blocking protein translation or inducing mRNA degradation [5]–[7]. mRNAs can be regulated by several miRNAs, and each miRNA can target hundreds of mRNAs in different binding sites [8], [9]. The recent evidence about the genetic variability at both 5′ and 3′ ends of the mature miRNA sequence generates a large spectrum of sequence variants called miRNA isoforms or isomiRs [10]–[12]. Mechanisms involved in isomiRs generation and their biological relevance has increased the complexity of molecular mechanisms related to regulation of mRNA expression mediated by miRNAs.

Nowadays, a large number of miRNAs have been reported in animals, plants and viruses, with up to 18,226 entries in the miRBase database (v18, November 2011, URL: http://www.mirbase.org/) [13]–[15]. In mammalian genomes, the number of encoded miRNAs has been predicted to be up to 1000 miRNAs, comprising about 3% of all protein-coding genes [5]. In mammals, nearly all miRNAs are conserved in closely related species [16], and may have homologous in distant species, suggesting that miRNA functions could also be conserved throughout the evolution of animal lineages [17]. Several studies showed that variability in miRNA sequences has been lost over time since miRNAs have been described in unicellular eukaryotes, showing its deeper evolutionary history among eukaryotes [18].

The pig is an important livestock species, not only for its production in industry, but also as a suitable animal model for comparative genomics and biomedical studies [19]. However, the number of porcine miRNAs available in public databases is still poor, with only 257 porcine miRNAs described in the pig genome compared to the completely sequenced human (1,921), mice (1,157), bovine (676), poultry (544), equine (360) or canine (289) genomes (miRBase v18). The description of the microRNAome (miRNAome) held by different tissues under different pathophysiological states has become of great interest in the last years [20]–[24]. Determining altered patterns of miRNA expression related to disease or specific treatments or conditions would be useful in order to identify differentially expressed miRNAs that could be used as novel biomarkers [25]–[30]. In pigs, several miRNAomes have been described in different tissues such as muscle, fat, heart, liver, thymus, intestine and testes [31]–[35], or using *in vitro* cells models such as porcine PK-15 cells (derived from porcine kidney epithelial cells) and porcine dendritic cells [36]–[38]. However, the porcine kidney miRNAome has not yet been described although it is an essential organ involved in functions like blood filtering, gluconeogenesis and in the secretion of important hormones like erythropoietin, renine and vitamin D.

The process of pig domestication has been very complex. Independent geographical origins have been described [39] which have generated multiple phenotypically different breeds [40], [41]. In particular, high differences are found regarding reproductive and meat production and quality traits between Asian and European breeds. In addition, pig has become an important production animal for human, and, consequently, it has been strongly selected for traits of economical interest. As a result of this high selection, many commercial breeds have been generated. Different consuming necessities in pig industry have expanded the large phenotypic variability due to different selected traits. Genetic diversity between pig breeds has been further studied [42]–[44] showing variability in gene expression and elucidating the artificial selection performed by porcine industry during pig domestication [45]. However, the genetic variability held at miRNA sequences, which could imply changes at post-transcriptional level, has not yet been explored although it may lead to great phenotypic differences and/or pathological disorders.

Studies with mitochondrial DNA (mtDNA) assume significant differences between European and Asian pigs prior to domestication and several studies describe the phylogenetic relationships between European and Asian pig breeds, determining different groups into European diversity and other groups into Asian diversity [39], [44], [46]. Other studies confirmed some introgression events of Asian pig breeds in most European commercial breeds and evidence that local Spanish breeds have never been introgressed with Asian neither with European commercial populations [47]–[49]. In this sense, studying miRNAs differentially expressed between European and Asian pig breeds could contribute to understand their phenotypical differences.

In the present work, the miRNAome of several pig breeds belonging to the pure European and Asian branches as well as European commercial breeds that have been introgressed with Asian genes has been undertaken. The main goals of this study were to describe the pig kidney miRNAome through high throughput sequencing (HTS) technology, to study the miRNA sequence variability among porcine breeds and to determinate new porcine miRNAs.

## Materials and Methods

### Samples and RNA preparation

Kidney samples were collected from twenty pigs belonging to six different breeds and the European ancestor (Wild boar). Animals were classified into three main groups based on breed origin; (1) European breeds: Wild boar (WB, n = 2) and Iberian (IB, n = 4), (2) Asian breeds: Meishan (ME, n = 3) and Vietnamese (VT, n = 3), and (3) European commercial breeds with strong influence from Asian breeds: Landrace (LD, n = 3), Large White (LW, n = 2) and Piétrain (PT, n = 3). All samples were taken from slaughterhouse (PRIMAYOR, Mollerussa, Spain) under veterinary supervision and they were immediately snap-frozen in liquid nitrogen and stored at −80°C until use. Total RNA was isolated using TRIzol® reagent (Invitrogen, Carlsbad, USA) following the manufacturer's recommendations. Total RNA was quantified using ND 1000 Nanodrop® Spectrophotometer (Thermo Scientific, Wilmington, USA) and its quality was assessed on an Agilent 2100 Bioanalyzer using the RNA 6000 Nano kits (Agilent Technologies, Santa Clara, USA). RNA quality threshold was set to RNA Integrity Number (RIN) above seven. For RT-qPCR validations, additional kidney samples were added (n = 29: 2 WB, 4 IB, 4 LD, 4 LW, 4 PT, 4 ME and 7 VT).

### Small RNA library construction and high throughput sequencing

Small RNA fraction (15–30 nt) was excised and isolated from denaturing 12.5% polyacrilamide gel electrophoresis (PAGE) using miSpike^TM^ (IDT®, Coralville, USA) as internal size marker. For each breed, 60 μg of total RNA were loaded on separate gels to avoid cross-contamination. Gels were stained with GelStar® Acid Nucleic Gel Stain (Lonza, Basel, Switzerland) for UV visualization. Excised small RNA fraction were purified using Performa® DTR gel filtration cartridges (EdgeBio, Gaithersburg, USA) and, then, isolated small RNAs were pooled by breed to construct small RNA libraries. Briefly, 3′ and 5′ linkers from miRCat^TM^ kit (IDT, Coralville, USA) were ligated at both ends of the small RNAs in two separated reactions using a T4 RNA ligase without ATP (Fermentas, Germany) and T4 RNA ligase with ATP (Ambion, Austin, USA), respectively. Between 3′ and 5′ primer ligations, the 60 nt RNAs were purified by PAGE to eliminate unligated products. Then, linked products were used to perform a reverse transcription reaction using the SuperScript^TM^ III Reverse Transcriptase kit (Invitrogen^TM^, Carlsbad, USA) and the cDNA obtained was amplified with the Platinum® Taq DNA Polimerase High Fidelity kit (Invitrogen^TM^, Carlsbad, USA). PCRs were done with primers complementary to 3′ and 5′ linkers and, in addition, they included multiplex identifiers at the 5′ end (a five nucleotide sequence tag) to allow differentiation between libraries (Table S1). Obtained PCR products were purified using the QIAquick PCR Purification Kit (Qiagen®, Germany). Libraries were quantified with Qubit^TM^ fluorometer, Quant-IT^TM^ (Invitrogen^TM^, Carlsbad, USA) and prepared to a 10^8^ DNA molecules/μL concentration for sequencing by 454 Genome Sequencer FLX Titanium (Roche, Germany) following the manufacturer's protocol at DNA sequencing facilities at CRAG (Bellaterra, Spain).

### Sequence annotation for miRNA expression profile and validation of selected miRNAs through Reverse Transcription quantitative real time PCR (RT-qPCR)

Primers were trimmed from sequences and only those sequences between 15 and 29 nucleotides and with total number of sequences ≥3 were kept for further analysis. Sequences were compared to all available miRNA sequences (miRBase v18) using local Blast. Parameters were set to 100% identity and up to 4 mismatches allowed at the end of the sequences to assume variability on 3′ and 5′ ends [31].

Differences in miRNA expression between breed groups (European breeds: WB and IB, Asian breeds: ME and VT, and European commercial breeds: LD, LW and PT) were assessed using the miRNAs annotated through miRBase database. Total number of sequences obtained for each miRNA was normalised by library size (in counts per thousand) and, then, averaged by group. Fold changes (FC) between groups were calculated using normalised data. According to sequence count, nine miRNAs highly expressed (Hsa-miR-200b-3p, Hsa-miR-200c-3p, Ssc-miR-126, Ssc-miR-126*, Ssc-miR-99a, Ssc-miR-532-5p, Ssc-miR-92a, Ssc-miR-26a and Bta-miR-193b, n>100) and four miRNAs lowly expressed (Ssc-miR-423-5p, Ssc-miR-29c, Ssc-miR-486 and Ssc-let-7f, n<100) in the kidney miRNAome were selected to measure their expression levels by RT-qPCR. Hsa-let-7a, Hsa-miR-25 and Hsa-miR-93 were used as reference miRNAs based on literature [50], [51]. Primers used were designed by Exiqon® (Denmark). For low expressed miRNAs and Bta-miR-193b primers were designed following the methodology suggested by Balcells et al [52] (Table 1), where it is described that miR-specific quantitative RT-qPCR with DNA primers is a highly specific, sensitive and accurate method for microRNA quantification.

**Table 1:** Primers used for the RT-qPCR validation design.

miRNA/Cluster	Forward primer (5′-3′)	Reverse primer (5′-3′)	Primer conc. (nM each)
Bta-miR-193b	AGAACTGGCCCACAAAGTC	TCCAGTTTTTTTTTTTTTTTAGCGG	250
Ssc-miR-423-5p	TGAGGGGCAGAGAGCGA	GTCCAGTTTTTTTTTTTTTTTAAAGTC	250
Ssc-miR-29c	GCAGTAGCACCATTTGAAATC	GGTCCAGTTTTTTTTTTTTTTTAACC	250
Ssc-miR-486	GCAGTCCTGTACTGAGCTG	GGTCCAGTTTTTTTTTTTTTTTCTCG	250
Ssc-let-7f	CGCAGTGAGGTAGTAGATTG	AGGTCCAGTTTTTTTTTTTTTTTAACT	250
Cl-2	CAGCTGCTATGCCAACA	CCAGTTTTTTTTTTTTTTTGGCAA	250
Cl-5	GCTGTAACAGCAACTCCA	TCCAGTTTTTTTTTTTTTTTCCAC	500
Cl-15	GCGACCCACTCTTGGT	TCCAGTTTTTTTTTTTTTTTCATGG	No amplification
Cl-16	CAGTTGGTGACCAGGTG	AGTTTTTTTTTTTTTTCTCCCTGA	No amplification
Cl-24	GCTGCATTTCCTGGCTG	GGTCCAGTTTTTTTTTTTTTTTAATTAAG	No amplification
Cl-25	CAGCTGGTGTTGTGAATC	GTTTTTTTTTTTTTTTCGGCCT	125
Cl-29	GGTTGGTGTACACTGGAA	GTCCAGTTTTTTTTTTTTTTTAGCT	250
Cl-38	GTCTCCGTTTGCCTGT	CCAGTTTTTTTTTTTTTTTCAGCA	250

Reverse transcription (RT) reactions were performed in duplicate with the universal cDNA synthesis kit (Exiqon, Denmark) using 300 ng of total RNA following the manufacturer's instructions. Non template controls (NTC) and minus poly(A) polymerase controls for each tissue were included. qPCRs were performed in duplicate using miRCURY^TM^ LNA^TM^ Universal RT microRNA PCR kit (Exiqon, Vedbaek, Denmark) on an 7900HT Sequence Detection System (Applied Biosystems, Warrington, UK). Each qPCR was done in 20 µL total volume including 10 µL SYBR Green master mix (Exiqon, Vedbaek, Denmark), 0.5 µL of each LNA primer set (Exiqon, Denmark) and 5 µl of a 1∶20 dilution of the cDNA. To assess qPCR efficiency, standard curves with 10-fold serial dilutions of a pool of equal amounts of cDNA from all samples were included in each assay. Thermal profile was set as follows: 95°C for 10 min and 40 cycles at 95°C for 15 sec and 60°C for 60 sec. NTC and minus poly(A) polymerase controls were included. Melting curve analysis was included at the end of the qPCR to detect unspecific amplifications.

Quantities from each sample were obtained from the calibration (standard) curve added in each RT-qPCR reaction. GeNorm v.3.5 software [53] was used to examine the stability of the reference miRNAs (M<1.5) and to obtain a normalization factor (NF). The quantity obtained from each miRNA and sample was normalised by the NF and FCs were calculated in relation to the lowest normalised value. FCs were log_2_ transformed and expression data were analysed by a two-way analysis of variance (ANOVA) with the General Linear Models procedure of the Statistical Package for the Social Scientists (IBM® SPSS® Statistics 19; IBM Corporation, Armonk, USA) including the RT and breed as fixed factors. Significance threshold was set at α<0.05. Scheffe test was used to determine significant differential expression between breed groups.

### Sequence analysis for novel miRNAs discovery and validation through RT-qPCR

All primer trimmed sequences were mapped in the pig genome retrieved from Ensembl Genome Browser (Ensembl release 62, ftp://ftp.ensembl.org/pub/release-62/fasta/sus_scrofa/dna/, Sscrofa9.62) considering 100% of alignment and identity (perfect match). Sequences that mapped only once in the genome and with a length from 19 to 23 nt were selected. Among these, only sequences with unknown annotation at Ensembl genome browser and with copy number (CN) higher than 2 were considered. They were clustered taking into account only the position in the genome. Hence, sequences positioned in the same region were grouped and the sequence with higher CN was selected as the reference sequence for each cluster. Fifteen clusters were considered novel miRNAs to be validated. Flanking regions (50 nt) of the selected reference sequences were used to predict pre-miRNA folding structure using MFold software [54] following the guidelines reported by Ambros et al. [55]. At the end, 8 clusters were selected for RT-qPCR validation. RT reactions were performed in duplicate using total RNA as previously described by Balcells et al [52]. Briefly, 600 ng of total RNA in a final volume of 20 μL including 2 μL of 10x poly(A) polymerase buffer, 0.1 mM of ATP, 0.1 mM of each dNTP, 1 μM of RT-primer, 200 U of M-MuLV Reverse Transcriptase (New England Biolabs, USA) and 2 U of poly(A) polimerase (New England Biolabs, USA) was incubated at 42°C for 1 hour and at 95°C for 5 minutes for enzyme inactivation. Non template controls (NTC) and minus poly(A) polymerase controls for each tissue were included.

DNA primers for each miRNA were designed following the methodology suggested by Balcells et al [52] (Table 1). qPCR reactions were performed in duplicate in 20 μL final volume including 10 μL FastStart Universal SYBR Green Master (Roche, Germany), 125–500 nM of each primer (Table 1) and 5 μL of a 1∶100 dilution of the cDNA. Standard curves were generated by 10 fold serial dilutions of a pool of all cDNAs in order to calculate the qPCR efficiency. For Cluster 2 (Cl-2), standard curve was performed by using 2-fold serial dilutions. qPCR settings and data analysis was performed as explained above. Hsa-let-7a, Hsa-miR-25 and Hsa-miR-103 were used as reference miRNAs [50], [51].

### Target prediction and functional analysis

DIANA – microT v3.0 web server [56], [57] was used to identify *in silico* potential mRNA targets for differentially expressed miRNAs. Porcine genes are not included in the current version of DIANA – microT v3.0 and predictions were based on the human mRNA: miRNA interactions assuming sequence conservation. *In silico* functional annotation of putative mRNA target genes for each miRNA were analyzed with WEB-based Gene Set Analysis Toolkit (WebGestalt, [58]). Predicted miRNA targets were functionally annotated through the biological process information supported by Gene Ontology (GO, [59]) and the pathways in which they were involved were described by using the Kyoto Encyclopedia of Genes and Genomes database (KEGG, [60], [61]). Over or under represented functional categories were identified with hypergeometric test corrected by the multiple test adjustment proposed by Benjamini & Hochberg [62]. Significant threshold was set at α<0.05.

## Results

### Characterisation of miRNA expression profile in porcine kidney

Small RNA libraries from kidney of six porcine breeds (Iberian, Landrace, Large White, Piétrain, Meishan and Vietnamese) and the European ancestor (Wild Boar) were sequenced in a high throughput 454 GS FLX sequencer (Roche). A total of 115,305 reads (corresponding to 17,913 unique sequences) containing short inserts of length ranging from 15 to 29 nt (corresponding to miRNA size) were obtained (Table 2). The total number of reads obtained per breed ranged from 13,704 to 23,815 counts; however, the Iberian breed yielded a much lower number of total counts (4,022). If we consider the number of unique sequences obtained per breed, as an average, 17% of total counts corresponded to unique sequences except for Iberian and Vietnamese breeds that had 39% and 38% of unique sequences, respectively.

**Table 2:** Summary of sequence alignment with miRBase database^1^.

Library	Item	Total	miRBase homology*	*Sus scrofa *homology*	Other species homology*	No homology in miRBase
Iberian (IB)	Total counts	4,022	2,748	1,897	851	1,274
	Unique sequences	1,575	512	289	223	1,063
	miRNAs		125	62	63	
Wild Boar (WB)	Total counts	18,391	14,787	7,146	7,641	3,604
	Unique sequences	3,480	1,295	726	569	2,185
	miRNAs		183	92	91	
Landrace (LD)	Total counts	13,704	11,131	4,897	6,234	2,573
	Unique sequences	2,670	982	545	437	1,688
	miRNAs		165	86	79	
Large White (LW)	Total counts	23,815	19,728	10,609	9,119	4,087
	Unique sequences	4,078	1,492	865	627	2,586
	miRNAs		191	99	92	
Piétrain (PT)	Total counts	20,215	16,971	9,890	7,081	3,244
	Unique sequences	3,307	1,351	781	570	1,956
	miRNAs		183	93	90	
Meishan (ME)	Total counts	17,407	14,990	5,380	9,610	2,417
	Unique sequences	2,607	1,079	597	482	1,528
	miRNAs		165	88	77	
Vietnamese (VT)	Total counts	17,751	9,239	5,105	4,134	8,512
	Unique sequences	6,695	1,022	577	445	5,673
	miRNAs		157	82	75	
**TOTAL**	Total counts	**115,305**	**89,594**	**44,924**	**44,670**	**25,711**
	Unique sequences	**17,913**	**3,624**	**2,007**	**1,617**	**14,289**
	miRNAs		**229**	**110**	**119**	

1 : miRBase database (v18, November 2011).

* Mismatches or gaps have not been allowed into the sequence alignment. Up to 4 mismatches allowed in the ends of the sequences.

Alignment of sequences to miRBase database revealed that 77.7% (89,594) of the total sequences yielded a positive match to a known miRNA. In total, 229 different miRNAs were found to be expressed in porcine kidney. Among them, 110 miRNAs corresponded to previously described porcine miRNAs and 119 to orthologous miRNAs. The percentage of detected miRNAs per breed varied from 55 to 83%, being the Iberian breed the library with less detected miRNAs and the Large White the most, in agreement with the total number of sequences obtained in these libraries. The proportion of porcine miRNAs and orthologous miRNAs was similar in all libraries, around 50% both. Sixty-two miRNAs had a CN below 3 and were excluded from the analysis. Overall, the porcine kidney miRNA profile was estimated to be of 167 miRNAs (Table S2). The most expressed miRNAs (CN>350, 0.30%) in porcine kidney are listed in Table 3. The most abundant miRNA was Hsa-miR-200b-3p (27,097, representing 23.50% of all porcine kidney miRNAs), followed by Ssc-miR-125b (8,809; 7.64%), Ssc-miR-23b (5,412; 4.69%), Ssc-miR-126 (5,274; 4.57%) and Ssc-miR-23a (5,156; 4.47%). The miRNA expression pattern was similar between breeds; however, some differences were found. As an example, Ssc-miR-125b was the most expressed miRNA in Iberian breed, followed by Ssc-miR-99a and Hsa-miR-200b-3p, and Ssc-miR-192 and Hsa-miR-200c-3p were the fourth and fifth most expressed miRNAs in Large White breed, respectively. In addition, Hsa-miR-200c-3p appeared as the fifth most expressed miRNA in Meishan breed.

**Table 3:** Most abundant miRNA profile in pig kidney (CN>350).

miRNA name	Total counts (%)	IsomiRs	IB	WB	LD	LW	PT	ME	VT
Hsa-miR-200b-3p	27,097 (23.50%)	123	239	4,924	4,298	5,163	3,683	6,987	1,803
Ssc-miR-125b	8,809 (7.64%)	51	567	1,245	933	2,170	2,322	798	774
Ssc-miR-23b	5,412 (4.69%)	59	116	992	638	935	1,090	673	968
Ssc-miR-126	5,274 (4.57%)	49	158	928	419	1,308	1,186	660	615
φSsc-miR-23a	5,156 (4.47%)	51	123	828	535	801	1,015	598	1,256
Ssc-miR-192	3,863 (3.35%)	41	89	723	194	1,105	810	484	458
φSsc-miR-99a	3,781 (3.28%)	32	339	615	375	884	888	234	446
Hsa-miR-200c-3p	3,478 (3.02%)	32	42	367	309	1,076	762	631	291
Ssc-miR-10b	2,846 (2.47%)	31	69	589	271	504	519	561	333
Ssc-miR-126*	2,796 (2.43%)	26	60	440	387	503	599	418	389
φSsc-miR-30d	1,977 (1.72%)	31	82	223	142	757	434	238	101
φSsc-miR-125a	1,369 (1.19%)	29	103	192	194	308	288	173	111
Ssc-miR-10a	1,317 (1.14%)	19	43	287	176	219	188	284	120
Ssc-miR-365-3p	986 (0.86%)	16	10	168	173	225	246	100	64
Ssc-miR-92a	797 (0.69%)	17	12	96	105	217	125	145	97
Ssc-miR-204	748 (0.65%)	14	10	61	271	173	133	58	42
Ssc-miR-378	743 (0.64%)	20	67	127	52	179	148	103	67
Ssc-miR-26a	598 (0.52%)	17	3	94	76	159	134	91	41
Bta-miR-200a	485 (0.42%)	9	18	87	99	95	58	101	27
φBta-miR-193b	473 (0.41%)	26	69	73	83	102	78	36	32
φSsc-miR-30e-5p	461 (0.40%)	14	27	57	43	134	93	56	51
Ssc-miR-100	405 (0.35%)	6	20	55	72	92	83	39	44
Ssc-miR-99b	350 (0.30%)	8	15	43	47	102	73	28	42

IB: Iberian breed, WB: European Wild Boar, LD: Landrace breed, LW: Large White breed, PT: Piétrain breed, ME: Meishan breed, VT: Vietnamese breed.

miRNA name represents the most expressed miRNA sequence in the cluster.

Underlined miRNAs correspond to miRNAs that had not been previously described.

φ: miRNA clusters whose predominant isomiR is different from that described in miRBase database (v18, November 2011).

### Sequence miRNA variation

**Figure 1:**
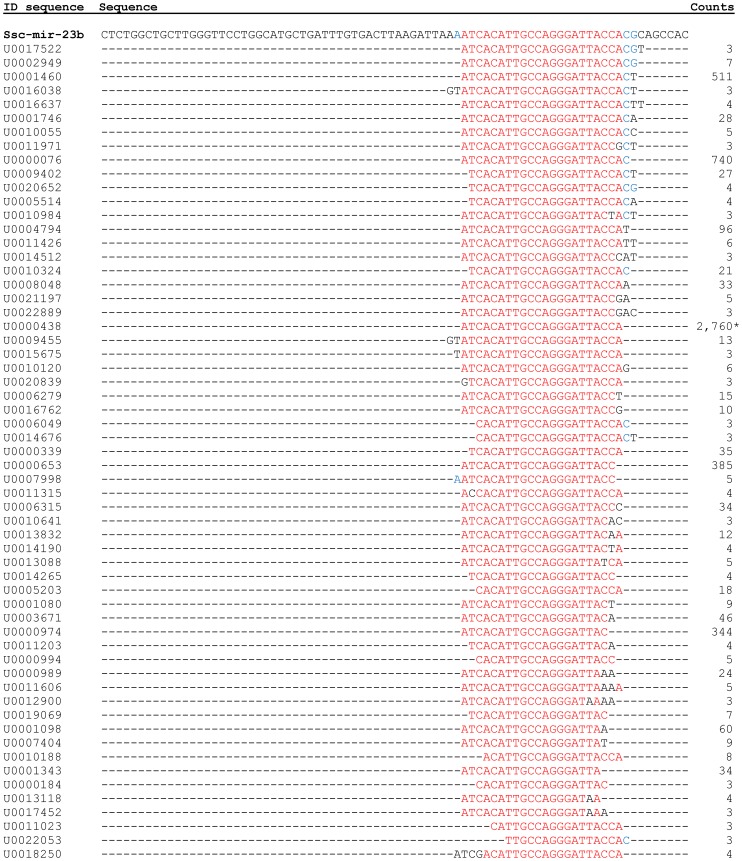
Sequence variability in Ssc-miR-23b. The number of total counts for each sequence is indicated. First sequence belongs to the precursor miRNA. Red bases match with precursor miRNA and belong to mature miRNA. Blue bases match also with precursor miRNA but do not belong to mature miRNA sequence. Sequence corresponding to the annotated miRNA in miRBase database is marked with an asterisk.

All miRNA sequences showed variability at both ends although it was higher in the 3′ end. In some cases, nucleotide variability could be also detected inside the miRNA sequence. In general terms, for each miRNA there was one sequence more expressed called reference miRNA sequence and, then, there was a number of sequence variations with less number of copies (Figure 1). Nucleotide variation was found in 70% of the miRNAs expressed in kidney and only a small proportion of kidney miRNAs did not show isomiRs. Nevertheless, those miRNAs without sequence variations were low expressed, from 3 to 38 total reads. Overall, 116 miRNAs presented isomiRs with an average rate of 10 isomiRs per miRNA. The miRNA with more variants was Hsa-miR-200b-3p with 123 variants, followed by Ssc-miR-23b and Ssc-miR-125b, with 59 and 51 variants, respectively. These three miRNAs are, in fact, the most expressed miRNAs in porcine kidney (Table 3). In this sense, variants expression followed two main patterns: according to those miRNAs with more than 1,000 total reads, they were distributed in those miRNAs with a strong predominant isomiR, such as Hsa-miR-200b-3p, Ssc-miR-125b, Ssc-miR-23b, Ssc-miR-23a, Ssc-miR-192, Ssc-miR-10b, Ssc-miR-126* and Ssc-miR-10a, and those miRNAs where there is not a really strong predominant isomiR, like Ssc-miR-126, Ssc-miR-99a, Hsa-miR-200c-3p, Ssc-miR-30d and Ssc-miR-125a (Table S3). Furthermore, the most expressed isoform was, in most cases, the same in all breeds; it only differed between breeds in Ssc-miR-99a and Has-miR-200c-3p.

Interestingly, reference miRNA sequence for each miRNA was not always the same as the one described in miRBase database. This was the case of 72 miRNAs (Table S4). Sequence differences were mainly found at 3′ end of the miRNA (in 90% of cases) and consisted, basically, in the addition or deletion of one or two nucleotides. In Table 4 are shown those most expressed miRNA sequences (CN>100) differing from the miRBase reference sequence. These differences in miRNA sequences are not influenced by the miRNA abundance, as total reads in these 72 miRNAs include high expressed miRNAs (Ssc-miR-23a, 5,156 reads) and low expressed miRNAs (Mmu-miR-5100, 3 reads). Moreover, counts of these 72 miRNAs represent the 21% of total counts of kidney miRNA profile, a considerable amount.

**Table 4:** Most expressed miRNAs diverging between the reference miRNA sequence and the miRBase^1^ described miRNA sequence.

miRNA name	Total counts	IsomiRs	Most expressed isomiR sequence (5′-3′)	Counts	Described miRNA sequence in miRBase database (5′-3′)	Counts
Ssc-miR-23a	5,156	51	ATCACATTGCCAGGGATTTCCA	3,116	ATCACATTGCCAGGGATTTCC	622
Ssc-miR-99a	3,781	32	AACCCGTAGATCCGATCTTGTGA	1,124	AACCCGTAGATCCGATCTTGTG	1,085
Ssc-miR-30d	1,977	31	TGTAAACATCCCCGACTGGAAGC	711	TGTAAACATCCCCGACTGGAAGCT	190
Ssc-miR-125a	1,369	29	TCCCTGAGACCCTTTAACCTGT	479	TCCCTGAGACCCTTTAACCTGTG	326
Bta-miR-193b	473	26	AACTGGCCCACAAAGTCCCGCT	196	AACTGGCCCACAAAGTCCCGCTTT	21
Ssc-miR-30e-5p	461	14	TGTAAACATCCTTGACTGGAAGC	268	TGTAAACATCCTTGACTGGAAGCT	74
Ssc-miR-139-5p	329	8	TCTACAGTGCACGTGTCTCCAGT	216	TCTACAGTGCACGTGTCTCCAG	85
Ssc-miR-30a-5p	314	10	TGTAAACATCCTCGACTGGAAGC	198	TGTAAACATCCTCGACTGGAAGCT	15
Ssc-miR-362	303	13	AATCCTTGGAACCTAGGTGTGAGT	133	AATCCTTGGAACCTAGGTGTGAGTG	0
Hsa-miR-29c-5p	260	20	GACCGATTTCTCCTGGTGTTCA	37	TGACCGATTTCTCCTGGTGTTC	22
Ssc-miR-374a	253	6	TTATAATACAACCTGATAAGTGT	117	TTATAATACAACCTGATAAGTG	13
Ssc-miR-145	222	12	GTCCAGTTTTCCCAGGAATCCCT	91	GTCCAGTTTTCCCAGGAATCCCTT	3
Hsa-miR-324-3p	211	22	ACTGCCCCAGGTGCTGCTGGT	45	ACTGCCCCAGGTGCTGCTGG	0
Ssc-miR-21	210	4	TAGCTTATCAGACTGATGTTGAC	105	TAGCTTATCAGACTGATGTTGA	77
Hsa-miR-874	195	8	CTGCCCTGGCCCGAGGGACCGAC	56	CTGCCCTGGCCCGAGGGACCGA	42
Ssc-miR-218b	185	7	TTGTGCTTGATCTAACCATGT	56	TTGTGCTTGATCTAACCATGTG	27
Ssc-miR-191	183	8	CAACGGAATCCCAAAAGCAGCT	63	CAACGGAATCCCAAAAGCAGCTG	45
Hsa-miR-150-5p	174	12	GTTCTCCCAACCCTTGTACCAGT	47	TCTCCCAACCCTTGTACCAGTG	14
Ssc-miR-22-3p	106	5	AAGCTGCCAGTTGAAGAAC	43	AAGCTGCCAGTTGAAGAACTGT	28

1 : miRBase database (v18, November 2011).

Bta: *Bos taurus*, Hsa: *Homo sapiens*, Ssc: *Sus scrofa*.

Marked in bold the nucleotide variation between pair sequences.

Furthermore, the most abundant reference sequence for a specific miRNA varied also between breeds. We found 76 miRNAs for which the most abundant sequence in each breed was not the same (data not shown). Nonetheless, differences in isomiR predominance between breeds did not follow any pattern related to breed origin. In addition, for some miRNAs, the described sequence in miRBase was expressed at a low level or even could not be detected in the porcine kidney samples analysed in this study. This was the case of Bta-miR-193b, Ssc-miR-374a, Ssc-miR-145, Ssc-miR-362, and Hsa-miR-324-3p, that had a CN of 21, 13, 3, 0 and 0, respectively.

### Between-breed kidney miRNA differential expression study

Differential miRNA expression analysis was performed between breed groups: EU, EA and AS. miRNAs were considered differentially expressed (DE) when fold change difference between groups was greater than 1.5 or when a miRNA was not expressed in one group although *p-value*s were not significant (*p-value*<0.05). In this sense, 42 miRNAs (25%) were equally expressed among analysed groups whereas 125 miRNAs (75%) were DE between breed groups (Table S5). About 77% of the described miRNAs were present in all groups. Focusing on miRNAs that were differential expressed from one group to the remaining groups, 26 miRNAs were up regulated in EU, 25 in EA and 7 in AS. Conversely, 16 miRNAs were down regulated only in EU, 9 in EA and 31 in AS (Table S5). Looking at the most expressed miRNAs, Ssc-miR-125b and Ssc-miR-99a were up regulated in EU, Hsa-miR-200c-3p and Ssc-miR-192 were up regulated in EA and Hsa-miR-200b-3p, Ssc-miR-23a and Ssc-miR-23b were up regulated in AS. Several miRNAs displayed high fold changes (FC≥5) in at least one comparison (Table S5); however, their CN tended to be low, suggesting that FCs were overestimated. Additionally, looking at normalised counts for each breed (Table S6), there were some miRNAs with low frequency which their expression was higher in some breed, such as Hsa-miR-193b-5p, Hsa-miR-140-5p, Ssc-miR-19b, Ssc-miR-423-5p and Ssc-miR-24 in Iberian breed, Ssc-miR-151-5p, Hsa-miR-4454, Ssc-miR-199a-3p and Ssc-miR-374b-5p in Landrace breed, Ssc-miR-486 in Piétrain breed and Ssc-let-7f and Hsa-let-7i-5p in Meishan breed. Thirteen miRNAs displaying expression differences between breeds by HTS were selected to be validated through RT-qPCR (Table 5). For RT-qPCR validations, additional kidney samples were added (n = 29: 2 WB, 4 IB, 4 LD, 4 LW, 4 PT, 4 ME and 7 VT). Hsa-miR-200b-3p, Hsa-miR-200c-3p and Bta-miR-193b were chosen to be orthologous miRNAs and thus, not described in pig yet. Ssc-miR-99a, Bta-miR-193b and Ssc-miR-423-5p were selected to be up regulated in EU, Hsa-miR-200b-3p, Ssc-miR-532-5p and Ssc-let-7f to be up regulated in AS and, Hsa-miR-200c-3p, Ssc-miR-92a, Ssc-miR-26a, Ssc-miR-486 and Ssc-miR-29c to be up regulated in EA. Finally, Ssc-miR-126 and Ssc-miR-126* were also included to be suggestively up regulated in EA. These miRNAs were mapped in the pig genome sequence (Sscrofa 9.62) and miRNA folding was predicted for Hsa-miR-200b-3p and Hsa-miR-200c-3p using MFold software [54] following the guidelines described by Ambros et al. [55]. Unfortunately, Bta-miR-193b could not be mapped in the pig genome and, consequently, its miRNA folding could not be predicted. RT-qPCR efficiencies obtained were high (ranging from 90% to 110%) and standard curves correlations were at least of 0.99, allowing us to perform a good gene expression quantification [63]. Eight miRNAs (Hsa-miR-200b-3p, Hsa-miR-200c-3p, Ssc-miR-126, Ssc-miR-126*, Ssc-miR-99a, Bta-miR-193b, Ssc-miR-486 and Ssc-let-7f) were differentially expressed in at least one comparison by RT-qPCR (*p-value*<0.05) (Table 6). Relative quantification from RT-qPCR data determined that Hsa-miR-200b-3p, Bta-miR-193b and Ssc-let-7f were up regulated in EU while Ssc-miR-126, Ssc-miR-126* and Ssc-miR-99a were down regulated in AS. On the other hand, Hsa-miR-200c-3p and Ssc-miR-486 were down and up regulated in EA, respectively. Correlation between HTS and qPCR data was medium to low (0.52>r2>0.003). Therefore, not all the results obtained with both techniques are in agreement.

**Table 5:** Selected miRNA and putative novel miRNAs (Clusters) for further validation through RT-qPCR.

miRNA/Cluster	Total counts	IsomiRs	Sequence (5′-3′)
Hsa-miR-200b-3p	27,097	123	TAATACTGCCTGGTAATGATGA
Ssc-miR-126	5,274	49	TCGTACCGTGAGTAATAATGCG
Ssc-miR-99a	3,781	32	AACCCGTAGATCCGATCTTGTGA
Hsa-miR-200c-3p	3,478	32	TAATACTGCCGGGTAATGATGGA
Ssc-miR-126*	2,796	26	CATTATTACTTTTGGTACGCG
Ssc-miR-92a	797	17	TATTGCACTTGTCCCGGCCTGT
Ssc-miR-26a	598	17	TTCAAGTAATCCAGGATAGGCT
Bta-miR-193b	493	26	AACTGGCCCACAAAGTCCCGCT
Ssc-miR-532-5p	113	3	CATGCCTTGAGTGTAGGACCGT
Ssc-miR-423-5p	74	10	TGAGGGGCAGAGAGCGAGACTTT
Ssc-miR-29c	23	3	TAGCACCATTTGAAATCGGTTA
Ssc-miR-486	23	4	TCCTGTACTGAGCTGCCCCGAG
Ssc-let-7f	17	1	TGAGGTAGTAGATTGTATAGTT
Cl-2 (Hsa-miR-31-3p)^1^	4	1	CTGCTATGCCAACATATTGCCA
Cl-5 (Hsa-miR-194-5p)^1^	12	2	CTGTAACAGCAACTCCATGTGGAA
Cl-15 (Hsa-miR-551a)^1^	9	2	GCGACCCACTCTTGGTTTCCATG
Cl-16	6	1	TTGGTGACCAGGTGCTCAGGGAG
Cl-24	3	2	CTGCATTTCCTGGCTGCCTTAATT
Cl-25 (Hsa-miR-138-5p)^1^	7	3	CAGCTGGTGTTGTGAATCAGGCCG
Cl-29	3	1	GTTGGTGTACACTGGAATAGCT
Cl-38 (Bta-miR-1468)^1^	21	3	TCTCCGTTTGCCTGTTTTGCTGA

1 : miRBase (v18) homology (e-value ≤1e-03). Internal mismatches were accepted.

Bta: *Bos taurus*, Hsa: *Homo sapiens*, *Ssc: Sus scrofa*.

**Table 6:** Fold Change (FC) comparison from HTS differential expression study and RT-qPCR data.

	HTS				RT-qPCR			
miRNA/Cluster	EU^2^ *vs*. EA^3^	EU *vs*. AS^4^	EA *vs*. AS	ANOVA group factor *p-value*	EU *vs*. EA	EU *vs*. AS	EA *vs*. AS	ANOVA group factor *p-value*
Hsa-miR-200b-3p	−1.45	−1.54	−1.06	0.794	1.29	1.50^*^	1.16	0.012
Ssc-miR-126	−1.07	1.24	1.32	0.576	1.09	1.60^***^	1.47^***^	<.001
Ssc-miR-99a	1.63	3.05	1.87	0.237	1.10	1.29^**^	1.18^§^	0.005
Hsa-miR-200c-3p	−2.31	−1.73	1.34	0.266	1.39	−1.15	−1.61^**^	0.009
Ssc-miR-126*	−1.36	−1.18	1.15	0.352	1.08	1.35^§^	1.25	0.044
Ssc-miR-92a	−1.87	−1.68	1.11	0.168	1.20	1.02	−1.18	0.614
Ssc-miR-26a	−2.15	−1.29	1.67	0.235	1.13	1.14	1.01	0.602
Bta-miR-193b	2.23	5.46	2.45	0.285	1.25	1.55^**^	1.25	0.003
Ssc-miR-532-5p	−1.16	−2.49	−2.14	0.082	1.05	−1.06	−1.12	0.685
Ssc-miR-423-5p	5.28	3.76	−1.40	0.110	1.09	1.14	1.05	0.416
Ssc-miR-29c	−3.78	1.45	5.47	0.028	1.01	1.20	1.19	0.105
Ssc-miR-486	−5.66	−1.55	3.64	0.179	−1.48^*^	−1.28	1.16	0.025
Ssc-let-7f	−2.76	−11.60	−4.21	0.344	1.22	1.33^*^	1.09	0.017
Cl-2 (Hsa-miR-31-3p)^1^	-	-	-	-	1.05	1.12	1.07	0.631
Cl-5 (Hsa-miR-194-5p)^1^	-	-	-	-	1.02	1.24	1.22^§^	0.049
Cl-25 (Hsa-miR-138-5p)^1^	-	-	-	-	1.35^**^	1.36^**^	1.00	0.003
Cl-29	-	-	-	-	−1.03	1.21	1.24^*^	0.009
Cl-38 (Bta-miR-1468)^1^	-	-	-	-	1.03	1.60^***^	1.55^***^	<.001

1 : miRBase (v18) homology (e-value≤1e-03). Internal mismatches were accepted.

2 : EU: European breeds. ^3^: EA: European commercial breeds. ^4^: AS: Asian breeds.

For HTS data, fold changes from sequence counting between breed groups were calculated from normalised data in counts per thousand for each library and averaged per groups. Positive and negative signs indicate that the level of gene expression is higher for the first or the second group of the test, respectively. Analysis of variance (ANOVA) including breed as fixed factor was performed. Significance was set at P<0.05. Fold changes for clusters could not be calculated due to their low total counts.

For qPCR data expression study, the quantity obtained of each miRNA in each sample was normalised by the Normalization Factor and corrected in relation to the lowest normalised value. Analysis of variance (ANOVA) including the RT and breed as fixed factors was performed and fold change from Least Squares Means (LSM) between breed groups were calculated. Significance was set at P<0.05. Scheffe test determined whether there was significant differential expression between breed groups (^§^: suggestive *p-value*<0.1, ^*^: significant with *p-value*<0.05, ^**^: significant with *p-value*<0.01, ^***^: significant with *p-value*<0.001).

### Validation of novel miRNAs through RT-qPCR

**Figure 2:**
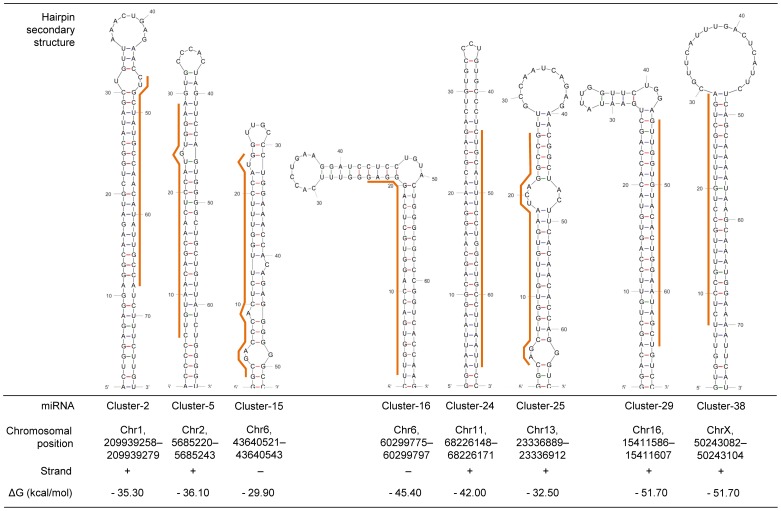
Predicted miRNA folding for cluster candidates to novel miRNAs using MFold software^1^. ^1^: [54]. Orange line points out the miRNA position into the pre-miRNA structure.

Unannotated sequences (486 unique sequences) that mapped in a unique region within the pig genome (Sscrofa 9.62) were clustered taking into account their position in the genome. After filtering by CN>2, 15 clusters remained for further analysis (Table S7). A compatible pre-miRNA folding structure could be successfully predicted *in silico* for 8 clusters (Cl-2, Cl-5, Cl-15, Cl-16, Cl-24, Cl-25, Cl-29 and Cl-38, Figure 2). Then, a validation protocol through RT-qPCR was designed for them. Five clusters (Cl-2, Cl-5, Cl-25, Cl-29 and Cl-38) were successfully amplified and, therefore, they have been considered as novel porcine miRNAs (Table 5). RT-qPCR efficiencies were high (ranging from 90% to 110%) and standard curves correlations were at least of 0.99. In addition, expression data showed that 4 clusters (Cl-5, Cl-25, Cl-29 and Cl-38) were differentially expressed (*p-value*<0.05) between breed groups (Table 6). Cl-5, Cl-29 and Cl-38 were down regulated in AS whereas Cl-25 was up regulated in EU.

To propose a nomenclature for the novel miRNAs, they were aligned to miRBase database accepting internal mismatches and some variation at the ends (Table 6). Cl-5 had a positive match with Hsa-miR-194-5p (*p-value* = 0.0004) with two nucleotide deletions at the ends, and also with Ssc-miR-194 (*p-value* = 0.002) although in this alignment one internal mismatch was detected in addition of the two nucleotide deletions at the ends. Cl-25 had homology with Hsa-miR-551a and Cl-29 did not match to any orthologous miRNA in miRBase database. Cl-38 matched with Bta-miR-1468 (*p-value* = 0.0003) with one nucleotide deletion in the 5′ end. Cl-2 was not differentially expressed through RT-qPCR but it matched with Hsa-miR-31-3p (*p-value* = 0.001) having two nucleotide deletion at the ends.

### Target prediction and functional analysis of differentially expressed porcine miRNAs

Target genes for the eight differentially expressed miRNAs (Hsa-miR-200b-3p, Hsa-miR-200c-3p, Ssc-miR-126, Ssc-miR-126^*^, Ssc-miR-99a, Bta-miR-193b, Ssc-miR-486 and Ssc-let-7f) were predicted *in silico*. A total of 1,884 target genes were identified (Table S8) and they were functionally analyzed through KEGG pathways database where 96 biological pathways were determined (data not shown). Interestingly, Hsa-miR-200b-3p and Hsa-miR-200c-3p, from the same miRNA family, practically share the same pathways, related to cellular processes, signal transduction and some biological processes, like immune, nervous, circulatory and endocrine systems. They have also been related to cancer pathways, like renal cell carcinoma, among others. Ssc-miR-126, Ssc-miR-126*, Ssc-miR-99a and Ssc-let-7f have also been described in some cancer pathways, emphasizing the importance of miRNAs in pathways related with cell cycle, growth and death. No significant related pathways were obtained from targets associated to Bta-miR-193b and Ssc-miR-486.

## Discussion

The present study represents the first work that describes the porcine kidney microRNAome in 6 different breeds and the European Ancestor, the Wild Boar, to determine differentially expressed miRNAs related to breed origin. A total of 110 out of 257 known porcine miRNAs in miRBase database have been determined in this study by high throughput sequencing. It is known that some miRNAs could be tissue specific [64] and this could explain why this study has only described the 43,28% of the porcine miRNAs reported up to date which is in agreement with other studies [22], [65]. Interestingly, among all described kidney miRNAs, 119 mammalian orthologous miRNAs were identified. Assuming high miRNA conservation in mammals [16], [66], the high proportion of orthologous miRNAs (52%) suggests that there are still many miRNAs not described in pigs yet.

miRNA expression profile in kidney revealed that the most expressed miRNAs were Hsa-miR-200b-3p, Ssc-miR-125b and Ssc-miR-23b. Interestingly, Hsa-miR-200b-3p is the most expressed miRNA in all breeds except for Iberian breed, which Ssc-miR-125b (567 reads), Ssc-miR-99a (339 reads) and Hsa-miR-200b-3p (239 reads) are the first, the second and the third most expressed miRNAs, respectively. This variation of the expression in Iberian breed could be explained by many reasons. Firstly, some bias could be assumed because of the low number of reads obtained in this library in comparison with the other libraries (Table 2). However, the total reads of each miRNA in Iberian breed evidence that there is some tendency in its expression. Secondly, this change in the most expressed miRNA could also be explained as Iberian breed was exclusively originated from European Wild Boar and not mixed with any other ancestor neither being in contact with other commercial breeds in Europe.

Importantly, the most expressed miRNA in kidney was Hsa-miR-200b-3p which has not already been described in pig. Its sequence could not be found in the previous pig genome sequence (v9.2) but it could be now annotated in the last version (Sscrofa10, ftp://ftp.ensembl.org/pub/release-67/fasta/sus_scrofa/dna/) probably due to it was located on a gap in the pig genome sequence that has been solved in the new version. Its expression was significantly higher than other profiled miRNAs, as it had been detected 27,097 times corresponding to the 23.50% of the total reads. This expression pattern in miRNAs profile has been defined in more studies [67], where a miRNA is predominant over the rest meaning that it could have a significant role in the expressed tissue. It is hypothesised that it could governs or be implicated on the major constitutive functions carried out by this tissue.

The multiple sequences obtained for each specific miRNA were aligned to study the variability in the 3′ and 5′ miRNA ends. Most of the identified miRNAs (70%) showed nucleotide polymorphisms and variations in sequence length, according to previous studies [31], [68]. One example in this study is Ssc-miR-23b, where the length varies from 18 to 25 nucleotides (Figure 1). This variability in the 5′ and 3′ ends could be favoured by processes prior to the high throughput sequencing sample preparation steps [69], being explained by the cleavage of the same miRNA precursor at different nucleotides during Drosha and Dicer processing. Another reason of this variability could be the nucleotide replacement and additions or deletions in the 5′ and 3′ miRNA ends. All this post-transcriptional mechanisms, called RNA editing, generate the isomiRs, defined as sequence variations in mature miRNAs and commonly reported in miRNA studies [10], [70]. Mechanisms of miRNA sequence diversification are extensively reviewed by Berezikov [16], suggesting a functional meaning to all these processes. Furthermore, different patterns described on isomiR expression suggest different strategies in the regulation of the expression. Assuming the expression data obtained in this study, there are miRNAs where the main isomiR could represent the major regulation function and there are miRNAs which their regulation function could be assumed by more than one isomiR (Table S3).

Considering the proportion of unique sequences obtained per breed in reference to the total sequences, Iberian and Vietnamese breeds obtained the highest proportion (38% and 39%, respectively) showing that, if the number of miRNAs was not increased in these libraries, what was increased was the number of described isomiRs. The percentage of unique sequences in the other libraries and also in the whole data (all libraries) was between 15 and 19%. Thus, the variability in miRNA sequences seemed to be higher in European breeds like Iberian breed and in Asian breeds such as Vietnamese breed. In contrast, when European commercial breeds were created through the European and Asian breeds introgression, their variability could be reduced due to their strong selection in production traits, as it is indicated if we consider the number of isomiRs in each European commercial breed studied.

Looking at the most expressed isomiR by miRNA in each breed we can determine that largely it was the same isomiR in all studied breeds. However, in 76 miRNAs, like Ssc-miR-99a, the most expressed isomiR varies depending on the breed, being more expressed one isomiR (U0009355: 5′-AACCCGTAGATCCGATCTTGTG-3′) in European and Asian breeds, and other isomiR (U0009353: 5′-AACCCGTAGATCCGATCTTGTGA-3′) in European commercial breeds. In other cases, there is an agreement in all breeds for the most expressed isomiR except in one breed, like in Ssc-miR-21 and Ssc-miR-100 in Iberian breed, Ssc-miR-152 in Piétrain breed, Has-miR-200c-3p in Vietnamese breed or Hsa-let-7d-5p in Landrace breed. There are also few cases where the most expressed isomiR is different in almost each breed, such as in Ssc-miR-199a* and Ssc-miR-423-5p. All these different situations suggest that the genetic variability degree between breeds is high and it could play an important role in the post-transcriptional regulation mechanisms leading to perform different functions.

Another variation factor was that there were some differences between the most expressed isomiR in each miRNA and its concordance with the described sequence in miRBase database, diverging them in 72 miRNAs found in this study. As example, the most representative sequence for Ssc-miR-23a in this study was 5′-ATCACATTGCCAGGGATTTCCA-3′, found 3,116 times and it corresponded to Bta-miR-23a. The sequence described in miRBase database for Ssc-miR-23a (5′-ATCACATTGCCAGGGATTTCC-3′) was found only 622 times (Table 4). It also suggests that there is not a fixed predominant isomiR, but there is variability in isomiR expression given not only by many factors like age, tissue or disease [71], but also by other factors like breed [50].

Despite of all miRNA homologies found, this study was also focused on describing novel miRNAs. The strict methodology used proposed 8 novel miRNAs to be validated through RT-qPCR and finally 5 miRNAs were successfully validated. These confirmed novel porcine miRNAs were mapped once in the pig genome (Table S7) and their pre-miRNA folding were successfully predicted (Figure 2). Moreover, three out of the thirteen selected miRNAs from the kidney miRNAome to be amplified through RT-qPCR were orthologous (Hsa-miR-200b-3p, Hsa-miR-200c-3p and Bta-miR-193b, Table 5) and, therefore, they were also confirmed as new porcine miRNAs. Significant differential expression regarding breed groups was obtained by RT-qPCR in eight miRNAs: Hsa-miR-200b-3p, Hsa-miR-200c-3p, Ssc-miR-126, Ssc-miR-126*, Ssc-miR-99a, Bta-miR-193b, Ssc-miR-486 and Ssc-let-7f. However, expression results from RT-qPCR were not in agreement with the differential expression study from high throughput sequencing data in some cases. These differences could be explained by many factors, like some possible bias, the fact that not each miRNA is amplified in the same proportion with high throughput sequencing [72], [73] and the presence of isomiRs, which make difficult to perform an accurate filtering of sequences. Other factors to be considered are some diversity and complexity in the miRNAs nomenclature between species listed in the miRNAs databases [15], which make difficult to perform a proper sequence classification. In this sense, cluster analysis methodology for novel miRNA detection was chosen to avoid problems with miRNA nomenclature. It is also important to consider the specificity in qPCR amplification, where primers designed in each miRNA were exclusive for the most expressed isomiR in each miRNA, amplifying only that one. Overall, the relevant topic of next generation sequencing about if it can be treated as expression data taking into account its large amount of reads, is still discussed [74]. The slightly different expression results between high throughput sequencing and RT-qPCR data reveal a change of expression tendency in some miRNAs evidencing the “semi-quantitative” nature of high throughput sequencing methods. Low correlations (from 0.52 to 0.003) were determined between high throughput sequencing by 454 (Roche, Germany) and RT-qPCR data. In this sense, the bias in this study could be explained by the lower number of sequences obtained. For instance, Hsa-miR-200b-3p appears in a higher expression in Asian breeds in sequencing data while it is more expressed in European breeds according to real time RT-qPCR data. Furthermore, Ssc-miR-126* was more represented in European commercial breeds, whereas in RT-qPCR it was up regulated in European breeds. The same case happened with Ssc-miR-126, a conclusive fact taking into account that both miRNAs are transcribed together. There are similar cases with low expressed miRNAs (according to sequence count) such as Ssc-let-7f, which was more expressed in Asian breeds but appears up regulated in European breeds in real time RT-qPCR data.

Analysing the target pathways of differentially expressed miRNAs between breed groups, it was found that Hsa-mir-200b-3p, the most expressed miRNA in this study and up regulated in European breeds, was related in several kidney diseases like tubulointerstitial fibrosis [75] or hypertensive glomerulosclerosis [12], elucidating the importance of this miRNA in kidney physiology. Targets of Hsa-miR-200b-3p were involved in several pathways like renal cell carcinoma, associated to some oncogenes such as MET, or tumors suppressors like VHL, FH and BHD. Interestingly, miRNA targets were related with the vascular smooth muscle contraction and calcium signaling pathways. Both pathways are important in muscular growth and development in which Hsa-miR-200b-3p, being more expressed in European pig breeds, could contribute to a greater efficiency. Furthermore, European commercial breeds are precisely selected for production traits and strongly characterized for animal production. Kidney and muscle are strongly related because kidney is in charge of secreting erythropoietin, the hormone responsible to activate erythropoiesis and also to transport oxygen to the muscles and other tissues, and thus, favoring muscular growth. Hsa-miR-200c-3p, which belongs to the same family that Hsa-miR-200b-3p, is also differentially expressed being up regulated in Asian breeds. Some studies have related it to some mechanisms of cancer development in many organs, such as pancreas, bladder, ovaries, prostate or breast. [76]–[80]. Functional analysis related targets of Hsa-miR-200c-3p with neoplasias, specifically in renal cell carcinoma. Furthermore, Hsa-miR-200c-3p is also involved in some pathways related to reproduction, like oocyte meiosis or progesterone-mediated oocyte maturation. Breeds with an Asian origin have better phenotypes for reproduction traits and, interestingly, Asian breeds have Hsa-miR-200c-3p up regulated suggesting its involvement in the regulation of the reproduction traits. However, Ssc-miR-99a and Hsa-miR-200b-3p targets are also related to these reproduction pathways, both up regulated miRNAs in European breeds which could play an antagonistic role in European breeds. Some targets of Ssc-miR-126, a down regulated miRNA in Asian breeds, participate in cellular processes like focal adhesion and regulation of actin cytoskeleton, while Ssc-miR-126*, also down regulated in Asian breeds, regulate targets implicated with cellular processes like adherens, gap and tight junction. Moreover, both miRNAs also have a strong relation with many cancer pathways, which were related in all miRNAs. Ssc-let-7f is an ubiquitous miRNA from let-7 family, related to many pathways involved in wide range of physiological functions (cellular, metabolic and environmental information processes) and also to many diseases, like renal cell carcinoma [81], [82].

This study opens a wide field about the miRNA specificity, which is not only by tissue, age or species level, but also breed factor is crucial to manage the phenotypic changes and, therefore, the genetic variability. The role of miRNAs in gene expression regulation is much more complex than it was thought initially, being necessary to study in depth which roles isomiRs play and to uncover all factors that make the post-transcriptional regulation through miRNAs so complex.

## Supporting Information

Table S1 **Adaptors used for the construction of each library.** ^1^: Forward adaptor, ^2^: Reverse adaptor. The 5 nt code used for each library is in bold in the adaptor sequence. Reverse adaptor was used in all breeds libraries.
(DOC)

Table S2 **Complete miRNA profile in healthy kidney of 6 porcine breeds and the European ancestor from 454 GS FLX run data.** IB: Iberian breed, WB: European Wild Boar, LD: Landrace breed, LW: Large White breed, PT: Piétrain breed, ME: Meishan breed, VT: Vietnamese breed. miRNA name represents the most expressed sequence in the cluster. Bta: *Bos taurus*, Dre: *Danio rerio*, Eca: *Equus caballus*, Hsa: *Homo sapiens*, Mdo: *Monodelphis domestica*, Mmu: *Mus musculus*, Rno: *Ratus norvegicus*, Sha: *Sarcophilus harrisii*, Ssc: *Sus scrofa*.
(DOC)

Table S3 **Summary of the isomiR distribution in the most expressed miRNAs** (**CN>1,000**)**.** miRNA name represents the most expressed sequence in the cluster. Hsa: *Homo sapiens*, Ssc: *Sus scrofa*.
(DOC)

Table S4 **Described miRNAs from HTS presenting variation between the reference miRNA sequence and the miRBase described miRNA sequence.** Bta: *Bos taurus*, Eca: *Ecuus caballus*, Hsa: *Homo sapiens*, Mdo: *Monodelphis domestica*, Mmu: *Mus musculus*, Rno: *Rattus norvegicus*, Sha: *Sarcophilus harrisii*, Ssc: *Sus scrofa*. Marked in bold the nucleotide variation between pair sequences.
(DOC)

Table S5 **List of differentially expressed miRNAs** (**Fold Change >1.5** **times**) **between porcine breed groups.** EU: European breeds; EA: European commercial breeds; AS: Asian breeds. Bta: *Bos taurus*, Dre: *Danio rerio*, Eca: *Equus caballus*, Hsa: *Homo sapiens*, Mdo: *Monodelphis domestica*, Mmu: *Mus musculus*, Rno: *Ratus norvegicus*, Sha: *Sarcophilus harrisii*, Ssc: *Sus scrofa*. Positive and negative signs indicate that the level of gene expression is higher for the first or the second group of the test, respectively.
(DOC)

Table S6 **Normalised 454 GS FLX run data by library in counts per thousand.** IB: Iberian breed, WB: European Wild Boar, LD: Landrace breed, LW: Large White breed, PT: Piétrain breed, ME: MeiShan breed, VT: Vietnamese breed. miRNA name represents the most expressed sequence in the cluster. Bta: *Bos taurus*, Dre: *Danio rerio*, Eca: *Equus caballus*, Hsa: *Homo sapiens*, Mdo: *Monodelphis domestica*, Mmu: *Mus musculus*, Rno: *Ratus norvegicus*, Sha: *Sarcophilus harrisii*, Ssc: *Sus scrofa*.
(DOC)

Table S7 **Resulting clusters from sequence analysis for novel miRNAs discovery.** ^1^: chromosome: start position: end position: strand. Sequences mapped at pig genome sequence (Sscrofa 9.62).
(DOC)

Table S8 **Putative target genes of the eight differentially expressed miRNAs analised by qRT-PCR.** Bta: *Bos taurus*, Hsa: *Homo sapiens*, Ssc: *Sus scrofa*. Potential mRNA target genes for differentially expressed miRNAs predicted *in silico* with DIANA – microT v3.0 web server.
(DOC)

## References

[1] Ambros V (2008) The evolution of our thinking about microRNAs. Nature Medicine 14: 1036–1040. 10.1038/nm1008-1036 18841144

[2] Ruvkun G (2008) The perfect storm of tiny RNAs. Nature Medicine 14: 1041–1045. 10.1038/nm1008-1041 18841145

[3] Bartel DP (2009) MicroRNAs: Target recognition and regulatory functions. Cell 136: 215–233. 19167326 10.1016/j.cell.2009.01.002 PMC3794896

[4] Krol J, Loedige I, Filipowicz W (2010) The widespread regulation of microRNA biogenesis, function and decay. Nature Reviews. Genetics 11: 597–610. 20661255 10.1038/nrg2843

[5] Bartel DP (2004) MicroRNAs: Genomics, biogenesis, mechanism, and function. Cell 116: 281–297. 14744438 10.1016/s0092-8674(04)00045-5

[6] Guo H, Ingolia NT, Weissman JS, Bartel DP (2010) Mammalian microRNAs predominantly act to decrease target mRNA levels. Nature 466: 835–840. 20703300 10.1038/nature09267 PMC2990499

[7] Huntzinger E, Izaurralde E (2011) Gene silencing by microRNAs: Contributions of translational repression and mRNA decay. Nature Reviews. Genetics 12: 99–110. 10.1038/nrg2936 21245828

[8] Ambros V (2004) The functions of animal microRNAs. Nature Medicine 431: 350–355. 10.1038/nature02871 15372042

[9] Lewis BP, Burge CB, Bartel DP (2005) Conserved seed pairing, often flanked by adenosines, indicates that thousands of human genes are microRNA targets. Cell 120: 15–20. 15652477 10.1016/j.cell.2004.12.035

[10] Morin RD, O'Connor MD, Griffith M, Kuchenbauer F, Delaney A, et al (2008) Application of massively parallel sequencing to microRNA profiling and discovery in human embryonic stem cells. Genome Research 18: 610–621. 18285502 10.1101/gr.7179508 PMC2279248

[11] Saal S, Harvey SJ (2009) MicroRNAs and the kidney: Coming of age. Current Opinion in Nephrology and Hypertension 18: 317–323. 19424061 10.1097/MNH.0b013e32832c9da2

[12] Li JY, Yong TY, Michael MZ, Gleadle JM (2010) Review: The role of microRNAs in kidney disease. Nephrology (Carlton, Vic.) 15: 599–608. 10.1111/j.1440-1797.2010.01363.x 20883280

[13] Kozomara A, Griffiths-Jones S (2011) miRBase: Integrating microRNA annotation and deep-sequencing data. Nucleic Acids Research 39: D152–7. 21037258 10.1093/nar/gkq1027 PMC3013655

[14] Griffiths-Jones S, Saini HK, van Dongen S, Enright AJ (2008) miRBase: Tools for microRNA genomics. Nucleic Acids Research 36: D154–8. 17991681 10.1093/nar/gkm952 PMC2238936

[15] Griffiths-Jones S, Grocock RJ, van Dongen S, Bateman A, Enright AJ (2006) miRBase: MicroRNA sequences, targets and gene nomenclature. Nucleic Acids Research 34: D140–4. 16381832 10.1093/nar/gkj112 PMC1347474

[16] Berezikov E (2011) Evolution of microRNA diversity and regulation in animals. Nature Reviews. Genetics 12: 846–860. 22094948 10.1038/nrg3079

[17] Lim LP, Glasner ME, Yekta S, Burge CB, Bartel DP (2003) Vertebrate microRNA genes. Science (New York, N.Y.) 299: 1540. 10.1126/science.1080372 12624257

[18] Tarver JE, Donoghue PC, Peterson KJ (2012) Do miRNAs have a deep evolutionary history? BioEssays: News and Reviews in Molecular, Cellular and Developmental Biology 34: 857–866. 10.1002/bies.201200055 22847169

[19] Lunney JK (2007) Advances in swine biomedical model genomics. International Journal of Biological Sciences 3: 179–184. 17384736 10.7150/ijbs.3.179 PMC1802015

[20] Glazov EA, Cottee PA, Barris WC, Moore RJ, Dalrymple BP, et al (2008) A microRNA catalog of the developing chicken embryo identified by a deep sequencing approach. Genome Research 18: 957–964. 18469162 10.1101/gr.074740.107 PMC2413163

[21] Huang TH, Zhu MJ, Li XY, Zhao SH (2008) Discovery of porcine microRNAs and profiling from skeletal muscle tissues during development. PloS One 3: e3225. 18795099 10.1371/journal.pone.0003225 PMC2528944

[22] Ribeiro-dos-Santos A, Khayat AS, Silva A, Alencar DO, Lobato J, et al (2010) Ultra-deep sequencing reveals the microRNA expression pattern of the human stomach. PloS One 5: e13205. 20949028 10.1371/journal.pone.0013205 PMC2951895

[23] Shao NY, Hu HY, Yan Z, Xu Y, Hu H, et al (2010) Comprehensive survey of human brain microRNA by deep sequencing. BMC Genomics 11: 409. 20591156 10.1186/1471-2164-11-409 PMC2996937

[24] Xie SS, Li XY, Liu T, Cao JH, Zhong Q, et al (2011) Discovery of porcine microRNAs in multiple tissues by a solexa deep sequencing approach. PloS One 6: e16235. 21283541 10.1371/journal.pone.0016235 PMC3026822

[25] Chen X, Ba Y, Ma L, Cai X, Yin Y, et al (2008) Characterization of microRNAs in serum: A novel class of biomarkers for diagnosis of cancer and other diseases. Cell Research 18: 997–1006. 18766170 10.1038/cr.2008.282

[26] Chow TF, Youssef YM, Lianidou E, Romaschin AD, Honey RJ, et al (2010) Differential expression profiling of microRNAs and their potential involvement in renal cell carcinoma pathogenesis. Clinical Biochemistry 43: 150–158. 19646430 10.1016/j.clinbiochem.2009.07.020

[27] Godwin JG, Ge X, Stephan K, Jurisch A, Tullius SG, et al (2010) Identification of a microRNA signature of renal ischemia reperfusion injury. Proceedings of the National Academy of Sciences of the United States of America 107: 14339–14344. 20651252 10.1073/pnas.0912701107 PMC2922548

[28] Patel N, Sauter ER (2011) Body fluid micro(mi)RNAs as biomarkers for human cancer. Journal of Nucleic Acids Investigation 2: e1.

[29] Fendler A, Stephan C, Yousef GM, Jung K (2011) MicroRNAs as regulators of signal transduction in urological tumors. Clinical Chemistry 57: 954–968. 21632885 10.1373/clinchem.2010.157727

[30] Catto JW, Alcaraz A, Bjartell AS, De Vere White R, Evans CP, et al (2011) MicroRNA in prostate, bladder, and kidney cancer: A systematic review. European Urology 59: 671–681. 21296484 10.1016/j.eururo.2011.01.044

[31] Nielsen M, Hansen JH, Hedegaard J, Nielsen RO, Panitz F, et al (2010) MicroRNA identity and abundance in porcine skeletal muscles determined by deep sequencing. Animal Genetics 41: 159–168. 10.1111/j.1365-2052.2009.01981.x 19917043

[32] Reddy AM, Zheng Y, Jagadeeswaran G, Macmil SL, Graham WB, et al (2009) Cloning, characterization and expression analysis of porcine microRNAs. BMC Genomics 10: 65. 19196471 10.1186/1471-2164-10-65 PMC2644714

[33] Cho IS, Kim J, Seo HY, Lim do H, Hong JS, et al (2010) Cloning and characterization of microRNAs from porcine skeletal muscle and adipose tissue. Molecular Biology Reports 37: 3567–3574. 20180025 10.1007/s11033-010-0005-6

[34] Sharbati S, Friedlander MR, Sharbati J, Hoeke L, Chen W, et al (2010) Deciphering the porcine intestinal microRNA transcriptome. BMC Genomics 11: 275. 20433717 10.1186/1471-2164-11-275 PMC2873480

[35] Lian C, Sun B, Niu S, Yang R, Liu B, et al.. (2012) A comparative profile of the microRNA transcriptome in immature and mature porcine testes using solexa deep sequencing. The FEBS Journal. 10.1111/j.1742-4658.2012.08480.x 22240065

[36] Wu YQ, Chen DJ, He HB, Chen DS, Chen LL, et al (2012) Pseudorabies virus infected porcine epithelial cell line generates a diverse set of host microRNAs and a special cluster of viral microRNAs. PloS One 7: e30988. 22292087 10.1371/journal.pone.0030988 PMC3264653

[37] Anselmo A, Flori L, Jaffrezic F, Rutigliano T, Cecere M, et al (2011) Co-expression of host and viral microRNAs in porcine dendritic cells infected by the pseudorabies virus. PloS One 6: e17374. 21408164 10.1371/journal.pone.0017374 PMC3050891

[38] Li S, Ran XQ, Xu L, Wang JF (2011) microRNA and mRNA expression profiling analysis of dichlorvos cytotoxicity in porcine kidney epithelial PK15 cells. DNA and Cell Biology 30: 1073–1083. 21702683 10.1089/dna.2011.1267

[39] Larson G, Dobney K, Albarella U, Fang M, Matisoo-Smith E, et al (2005) Worldwide phylogeography of wild boar reveals multiple centers of pig domestication. Science (New York, N.Y.) 307: 1618–1621. 10.1126/science.1106927 15761152

[40] Larson G, Albarella U, Dobney K, Rowley-Conwy P, Schibler J, et al (2007) Ancient DNA, pig domestication, and the spread of the neolithic into europe. Proceedings of the National Academy of Sciences of the United States of America 104: 15276–15281. 17855556 10.1073/pnas.0703411104 PMC1976408

[41] Larson G, Liu R, Zhao X, Yuan J, Fuller D, et al (2010) Patterns of east asian pig domestication, migration, and turnover revealed by modern and ancient DNA. Proceedings of the National Academy of Sciences of the United States of America 107: 7686–7691. 20404179 10.1073/pnas.0912264107 PMC2867865

[42] Amaral AJ, Ferretti L, Megens HJ, Crooijmans RP, Nie H, et al (2011) Genome-wide footprints of pig domestication and selection revealed through massive parallel sequencing of pooled DNA. PloS One 6: e14782. 21483733 10.1371/journal.pone.0014782 PMC3070695

[43] Ojeda A, Ramos-Onsins SE, Marletta D, Huang LS, Folch JM, et al (2011) Evolutionary study of a potential selection target region in the pig. Heredity 106: 330–338. 20502482 10.1038/hdy.2010.61 PMC3183885

[44] Giuffra E, Kijas JM, Amarger V, Carlborg O, Jeon JT, et al (2000) The origin of the domestic pig: Independent domestication and subsequent introgression. Genetics 154: 1785–1791. 10747069 10.1093/genetics/154.4.1785 PMC1461048

[45] Gao Y, Zhang YH, Jiang H, Xiao SQ, Wang S, et al (2011) Detection of differentially expressed genes in the longissimus dorsi of northeastern indigenous and large white pigs. Genetics and Molecular Research: GMR 10: 779–791. 21563072 10.4238/vol10-2gmr1170

[46] Fang M, Andersson L (2006) Mitochondrial diversity in european and chinese pigs is consistent with population expansions that occurred prior to domestication. Proceedings.Biological Sciences/the Royal Society 273: 1803–1810. 10.1098/rspb.2006.3514 PMC1634785 16790414

[47] Clop A, Amills M, Noguera JL, Fernandez A, Capote J, et al (2004) Estimating the frequency of asian cytochrome B haplotypes in standard european and local spanish pig breeds. Genetics, Selection, Evolution: GSE 36: 97–104. 10.1186/1297-9686-36-1-97 PMC2697182 14713412

[48] Alves E, Ovilo C, Rodriguez MC, Silio L (2003) Mitochondrial DNA sequence variation and phylogenetic relationships among iberian pigs and other domestic and wild pig populations. Animal Genetics 34: 319–324. 14510666 10.1046/j.1365-2052.2003.01010.x

[49] Kim KI, Lee JH, Li K, Zhang YP, Lee SS, et al (2002) Phylogenetic relationships of asian and european pig breeds determined by mitochondrial DNA D-loop sequence polymorphism. Animal Genetics 33: 19–25. 11849133 10.1046/j.1365-2052.2002.00784.x

[50] Timoneda O, Balcells I, Cordoba S, Castello A, Sanchez A (2012) Determination of reference microRNAs for relative quantification in porcine tissues. PloS One 7: e44413. 22970213 10.1371/journal.pone.0044413 PMC3438195

[51] Peltier HJ, Latham GJ (2008) Normalization of microRNA expression levels in quantitative RT-PCR assays: Identification of suitable reference RNA targets in normal and cancerous human solid tissues. RNA (New York, N.Y.) 14: 844–852. 10.1261/rna.939908 PMC2327352 18375788

[52] Balcells I, Cirera S, Busk PK (2011) Specific and sensitive quantitative RT-PCR of miRNAs with DNA primers. BMC Biotechnology 11: 70. 21702990 10.1186/1472-6750-11-70 PMC3135530

[53] Vandesompele J, De Preter K, Pattyn F, Poppe B, Van Roy N, et al (2002) Accurate normalization of real-time quantitative RT-PCR data by geometric averaging of multiple internal control genes. Genome Biology 3: RESEARCH0034. 12184808 10.1186/gb-2002-3-7-research0034 PMC126239

[54] Zuker M (2003) Mfold web server for nucleic acid folding and hybridization prediction. Nucleic Acids Research 31: 3406–3415. 12824337 10.1093/nar/gkg595 PMC169194

[55] Ambros V, Bartel B, Bartel DP, Burge CB, Carrington JC, et al (2003) A uniform system for microRNA annotation. RNA (New York, N.Y.) 9: 277–279. 10.1261/rna.2183803 PMC1370393 12592000

[56] Maragkakis M, Alexiou P, Papadopoulos GL, Reczko M, Dalamagas T, et al (2009) Accurate microRNA target prediction correlates with protein repression levels. BMC Bioinformatics 10: 295. 19765283 10.1186/1471-2105-10-295 PMC2752464

[57] Maragkakis M, Reczko M, Simossis VA, Alexiou P, Papadopoulos GL, et al (2009) DIANA-microT web server: Elucidating microRNA functions through target prediction. Nucleic Acids Research 37: W273–6. 19406924 10.1093/nar/gkp292 PMC2703977

[58] Zhang B, Kirov S, Snoddy J (2005) WebGestalt: An integrated system for exploring gene sets in various biological contexts. Nucleic Acids Research 33: W741–8. 15980575 10.1093/nar/gki475 PMC1160236

[59] Ashburner M, Ball CA, Blake JA, Botstein D, Butler H, et al (2000) Gene ontology: Tool for the unification of biology. the gene ontology consortium. Nature Genetics 25: 25–29. 10802651 10.1038/75556 PMC3037419

[60] Kanehisa M, Goto S (2000) KEGG: Kyoto encyclopedia of genes and genomes. Nucleic Acids Res. 28: 27–30. 10.1093/nar/28.1.27 PMC102409 10592173

[61] Ogata H, Goto S, Sato K, Fugibuchi W, Bono H, et al (1999) KEGG: Kyoto encyclopedia of genes and genomes. Nucleic Acids Res. 27: 29–34. 10.1093/nar/27.1.29 PMC148090 9847135

[62] Benjamini Y, Hochberg Y (1995) Controlling the false discovery rate – a practical and powerful approach to multiple testing rid C-4219-2008. J. R. Stat. Soc. Ser. B Stat. Methodol. 57: 283–300.

[63] Bustin SA, Benes V, Garson JA, Hellemans J, Huggett J, et al (2009) The MIQE guidelines: Minimum information for publication of quantitative real-time PCR experiments. Clinical Chemistry 55: 611–622. 19246619 10.1373/clinchem.2008.112797

[64] Lagos-Quintana M, Rauhut R, Yalcin A, Meyer J, Lendeckel W, et al (2002) Identification of tissue-specific microRNAs from mouse. Current Biology: CB 12: 735–739. 12007417 10.1016/s0960-9822(02)00809-6

[65] Li HY, Xi QY, Xiong YY, Liu XL, Cheng X, et al.. (2012) Identification and comparison of microRNAs from skeletal muscle and adipose tissues from two porcine breeds. Animal Genetics. 10.1111/j.1365-2052.2012.02332.x 22497549

[66] Lau NC, Lim LP, Weinstein EG, Bartel DP (2001) An abundant class of tiny RNAs with probable regulatory roles in caenorhabditis elegans. Science (New York, N.Y.) 294: 858–862. 10.1126/science.1065062 11679671

[67] Zhou Y, Tang X, Song Q, Ji Y, Wang H, et al.. (2012) Identification and characterization of pig embryo MicroRNAs by solexa sequencing. Reproduction in Domestic Animals = Zuchthygiene. 10.1111/j.1439-0531.2012.02040.x 22646905

[68] Landgraf P, Rusu M, Sheridan R, Sewer A, Iovino N, et al (2007) A mammalian microRNA expression atlas based on small RNA library sequencing. Cell 129: 1401–1414. 17604727 10.1016/j.cell.2007.04.040 PMC2681231

[69] Lee LW, Zhang S, Etheridge A, Ma L, Martin D, et al (2010) Complexity of the microRNA repertoire revealed by next-generation sequencing. RNA (New York, N.Y.) 16: 2170–2180. 10.1261/rna.2225110 PMC2957056 20876832

[70] Fernandez-Valverde SL, Taft RJ, Mattick JS (2010) Dynamic isomiR regulation in drosophila development. RNA (New York, N.Y.) 16: 1881–1888. 10.1261/rna.2379610 PMC2941097 20805289

[71] Li SC, Liao YL, Ho MR, Tsai KW, Lai CH, et al (2012) miRNA arm selection and isomiR distribution in gastric cancer. BMC Genomics 13 Suppl 1S13. 10.1186/1471-2164-13-S1-S13 PMC3303722 22369582

[72] Dohm JC, Lottaz C, Borodina T, Himmelbauer H (2008) Substantial biases in ultra-short read data sets from high-throughput DNA sequencing. Nucleic Acids Research 36: e105. 18660515 10.1093/nar/gkn425 PMC2532726

[73] Taub MA, Corrada Bravo H, Irizarry RA (2010) Overcoming bias and systematic errors in next generation sequencing data. Genome Medicine 2: 87. 21144010 10.1186/gm208 PMC3025429

[74] Malone JH, Oliver B (2011) Microarrays, deep sequencing and the true measure of the transcriptome. BMC Biology 9: 34. 21627854 10.1186/1741-7007-9-34 PMC3104486

[75] Oba S, Kumano S, Suzuki E, Nishimatsu H, Takahashi M, et al (2010) miR-200b precursor can ameliorate renal tubulointerstitial fibrosis. PloS One 5: e13614. 21049046 10.1371/journal.pone.0013614 PMC2963611

[76] Li Y, VandenBoom TG,2nd, Kong D, Wang Z, Ali S, et al (2009) Up-regulation of miR-200 and let-7 by natural agents leads to the reversal of epithelial-to-mesenchymal transition in gemcitabine-resistant pancreatic cancer cells. Cancer Research 69: 6704–6712. 19654291 10.1158/0008-5472.CAN-09-1298 PMC2727571

[77] Han Y, Chen J, Zhao X, Liang C, Wang Y, et al (2011) MicroRNA expression signatures of bladder cancer revealed by deep sequencing. PloS One 6: e18286. 21464941 10.1371/journal.pone.0018286 PMC3065473

[78] Marchini S, Cavalieri D, Fruscio R, Calura E, Garavaglia D, et al (2011) Association between miR-200c and the survival of patients with stage I epithelial ovarian cancer: A retrospective study of two independent tumour tissue collections. The Lancet Oncology 12: 273–285. 21345725 10.1016/S1470-2045(11)70012-2

[79] Tang X, Tang X, Gal J, Kyprianou N, Zhu H, et al (2011) Detection of microRNAs in prostate cancer cells by microRNA array. Methods in Molecular Biology (Clifton, N.J.) 732: 69–88. 10.1007/978-1-61779-083-6_6 21431706

[80] Bockmeyer CL, Christgen M, Muller M, Fischer S, Ahrens P, et al.. (2011) MicroRNA profiles of healthy basal and luminal mammary epithelial cells are distinct and reflected in different breast cancer subtypes. Breast Cancer Research and Treatment. 10.1007/s10549-010-1303-3 21409395

[81] Gottardo F, Liu CG, Ferracin M, Calin GA, Fassan M, et al (2007) Micro-RNA profiling in kidney and bladder cancers. Urologic Oncology 25: 387–392. 17826655 10.1016/j.urolonc.2007.01.019

[82] White NM, Bui A, Mejia-Guerrero S, Chao J, Soosaipillai A, et al (2010) Dysregulation of kallikrein-related peptidases in renal cell carcinoma: Potential targets of miRNAs. Biological Chemistry 391: 411–423. 20180642 10.1515/BC.2010.041

