# Supplementary material for: miRNA Expression Profile Analysis in Kidney of Different Porcine Breeds
Source: PLoS One. 2013 Jan 25;8(1):e55402. doi: 10.1371/journal.pone.0055402 (PMC3555835; doi:10.1371/journal.pone.0055402)
Supplement: Table S1 — Adaptors used for the construction of each library. 1: Forward adaptor, 2: Reverse adaptor. The 5 nt code used for each library is in bold in the adaptor sequence. Reverse adaptor was used in all breeds libraries. (DOC) [file pone.0055402.s001.doc]

Table S1. Adaptors used for the construction of each library.

| **Reference** | **Fwd^1^/Rev^2^** | **Adaptor sequence (5’-3’)** |
| --- | --- | --- |
| Wild Boar | Fwd | GCCTCCCTCGCGCCATCA**GTATG**CTGGAATTCTCGGGCACC |
| Iberian | Fwd | GCCTCCCTCGCGCCATCA**GCTAG**ATGGAATTCTCGGGCACC |
| Ladrace | Fwd | GCCTCCCTCGCGCCATCA**GCATC**ATGGAATTCTCGGGCACC |
| Large White | Fwd | GCCTCCCTCGCGCCATCA**GTGCG**ATGGAATTCTCGGGCACC |
| Piétrain | Fwd | GCCTCCCTCGCGCCATCA**GCTAC**GTGGAATTCTCGGGCACC |
| Meishan | Fwd | GCCTCCCTCGCGCCATCA**GAGTA**CTGGAATTCTCGGGCACC |
| Vietnamese | Fwd | GCCTCCCTCGCGCCATCA**GTCAT**CTGGAATTCTCGGGCACC |
| All breeds | Rev | GCCTTGCCAGCCCGCTCAG**ATAGC**GATTGATGGTGCCTACAG |

^1^:Forward adaptor, ^2^:Reverse adaptor.
The 5 nt code used for each library is in bold in the adaptor sequence. Reverse adaptor was used in all breeds libraries.
