# Supplementary material for: miRNA Expression Profile Analysis in Kidney of Different Porcine Breeds
Source: PLoS One. 2013 Jan 25;8(1):e55402. doi: 10.1371/journal.pone.0055402 (PMC3555835; doi:10.1371/journal.pone.0055402)
Supplement: Table S2 — Complete miRNA profile in healthy kidney of 6 porcine breeds and the European ancestor from 454 GS FLX run data. IB: Iberian breed, WB: European Wild Boar, LD: Landrace breed, LW: Large White breed, PT: Piétrain breed, ME: Meishan breed, VT: Vietnamese breed. miRNA name represents the most expressed sequence in the cluster. Bta: Bos taurus, Dre: Danio rerio, Eca: Equus caballus, Hsa: Homo sapiens, Mdo: Monodelphis domestica, Mmu: Mus musculus, Rno: Ratus norvegicus, Sha: Sarcophilus harrisii, Ssc: Sus scrofa. (DOC) [file pone.0055402.s002.doc]

**Table S2. Complete miRNA profile in healthy kidney of 6 porcine breeds and the European ancestor from 454 GS FLX** run data.

| **miRNA name** | **Total counts** | **IsomiRs** | **IB** | **WB** | **LD** | **LW** | **PT** | **ME** | **VT** |
| --- | --- | --- | --- | --- | --- | --- | --- | --- | --- |
| Hsa-miR-200b-3p | 27,097 | 123 | 239 | 4,924 | 4,298 | 5,163 | 3,683 | 6,987 | 1,803 |
| Ssc-miR-125b | 8,809 | 51 | 567 | 1,245 | 933 | 2,170 | 2,322 | 798 | 774 |
| Ssc-miR-23b | 5,412 | 59 | 116 | 992 | 638 | 935 | 1,090 | 673 | 968 |
| Ssc-miR-126 | 5,274 | 49 | 158 | 928 | 419 | 1,308 | 1,186 | 660 | 615 |
| Ssc-miR-23a | 5,156 | 51 | 123 | 828 | 535 | 801 | 1,015 | 598 | 1,256 |
| Ssc-miR-192 | 3,863 | 41 | 89 | 723 | 194 | 1,105 | 810 | 484 | 458 |
| Ssc-miR-99a | 3,781 | 32 | 339 | 615 | 375 | 884 | 888 | 234 | 446 |
| Hsa-miR-200c-3p | 3,478 | 32 | 42 | 367 | 309 | 1,076 | 762 | 631 | 291 |
| Ssc-miR-10b | 2,846 | 31 | 69 | 589 | 271 | 504 | 519 | 561 | 333 |
| Ssc-miR-126* | 2,796 | 26 | 60 | 440 | 387 | 503 | 599 | 418 | 389 |
| Ssc-miR-30d | 1,977 | 31 | 82 | 223 | 142 | 757 | 434 | 238 | 101 |
| Ssc-miR-125a | 1,369 | 29 | 103 | 192 | 194 | 308 | 288 | 173 | 111 |
| Ssc-miR-10a | 1,317 | 19 | 43 | 287 | 176 | 219 | 188 | 284 | 120 |
| Ssc-miR-365-3p | 986 | 16 | 10 | 168 | 173 | 225 | 246 | 100 | 64 |
| Ssc-miR-92a | 797 | 17 | 12 | 96 | 105 | 217 | 125 | 145 | 97 |
| Ssc-miR-204 | 748 | 14 | 10 | 61 | 271 | 173 | 133 | 58 | 42 |
| Ssc-miR-378 | 743 | 20 | 67 | 127 | 52 | 179 | 148 | 103 | 67 |
| Ssc-miR-26a | 598 | 17 | 3 | 94 | 76 | 159 | 134 | 91 | 41 |
| Bta-miR-200a | 485 | 9 | 18 | 87 | 99 | 95 | 58 | 101 | 27 |
| Bta-miR-193b | 473 | 26 | 69 | 73 | 83 | 102 | 78 | 36 | 32 |
| Ssc-miR-30e-5p | 461 | 14 | 27 | 57 | 43 | 134 | 93 | 56 | 51 |
| Ssc-miR-100 | 405 | 6 | 20 | 55 | 72 | 92 | 83 | 39 | 44 |
| Ssc-miR-99b | 350 | 8 | 15 | 43 | 47 | 102 | 73 | 28 | 42 |
| Ssc-miR-139-5p | 329 | 8 | 13 | 48 | 22 | 106 | 68 | 42 | 30 |
| Ssc-miR-324 | 314 | 7 | 14 | 24 | 11 | 136 | 92 | 22 | 15 |
| Ssc-miR-30a-5p | 314 | 10 | 17 | 39 | 41 | 91 | 59 | 43 | 24 |
| Ssc-miR-362 | 303 | 13 | 4 | 22 | 5 | 144 | 75 | 26 | 27 |
| Ssc-miR-429 | 287 | 10 | 4 | 45 | 67 | 46 | 51 | 51 | 23 |
| Hsa-miR-29c-5p | 260 | 20 | 7 | 50 | 17 | 55 | 66 | 30 | 35 |
| Ssc-miR-374a | 253 | 6 | 1 | 32 | 39 | 50 | 49 | 63 | 19 |
| Hsa-miR-500a-5p | 246 | 12 | 8 | 17 | 34 | 63 | 66 | 42 | 16 |
| Ssc-miR-145 | 222 | 12 | 18 | 26 | 26 | 45 | 63 | 26 | 18 |
| Hsa-miR-324-3p | 211 | 22 | 18 | 33 | 30 | 43 | 33 | 30 | 24 |
| Ssc-miR-21 | 210 | 4 | 1 | 35 | 27 | 58 | 49 | 20 | 20 |
| Hsa-miR-25-3p | 198 | 7 | 11 | 22 | 36 | 50 | 41 | 30 | 8 |
| Hsa-miR-874 | 195 | 8 | 8 | 23 | 22 | 57 | 53 | 19 | 13 |
| Ssc-miR-218b | 185 | 7 | 2 | 45 | 10 | 35 | 32 | 38 | 23 |
| Ssc-miR-191 | 183 | 8 | 14 | 32 | 9 | 55 | 37 | 23 | 13 |
| Hsa-miR-150-5p | 174 | 12 | 4 | 26 | 36 | 48 | 25 | 28 | 7 |
| Ssc-miR-193a-5p | 148 | 4 | 20 | 32 | 18 | 19 | 16 | 38 | 5 |
| Ssc-let-7a | 125 | 6 | 2 | 15 | 20 | 20 | 16 | 32 | 20 |
| Ssc-miR-532-5p | 113 | 3 | 2 | 14 | 11 | 19 | 12 | 35 | 20 |
| Ssc-miR-22-3p | 106 | 5 | 5 | 29 | 16 | 20 | 15 | 8 | 13 |
| Ssc-miR-450b-5p | 97 | 7 | 0 | 18 | 6 | 15 | 22 | 19 | 17 |
| Ssc-miR-664-5p | 89 | 6 | 4 | 11 | 9 | 31 | 15 | 17 | 2 |
| Ssc-miR-374b-5p | 86 | 5 | 1 | 7 | 32 | 21 | 8 | 12 | 5 |
| Hsa-let-7b-5p | 77 | 7 | 1 | 11 | 13 | 13 | 17 | 13 | 9 |
| Ssc-miR-29b | 75 | 5 | 5 | 14 | 1 | 26 | 18 | 4 | 7 |
| Ssc-miR-151-3p | 75 | 10 | 5 | 15 | 15 | 17 | 8 | 12 | 3 |
| Hsa-miR-29a-5p | 75 | 5 | 4 | 9 | 2 | 22 | 27 | 5 | 6 |
| Ssc-miR-532-3p | 75 | 6 | 7 | 7 | 7 | 20 | 16 | 13 | 5 |
| Ssc-miR-24 | 74 | 7 | 11 | 6 | 9 | 22 | 10 | 10 | 6 |
| Ssc-miR-423-5p | 74 | 10 | 12 | 20 | 4 | 10 | 9 | 10 | 9 |
| Ssc-miR-186 | 73 | 6 | 4 | 5 | 17 | 11 | 7 | 22 | 7 |
| Hsa-let-7d-5p | 68 | 4 | 4 | 14 | 3 | 12 | 8 | 15 | 12 |
| Ssc-miR-500 | 68 | 8 | 3 | 16 | 5 | 22 | 8 | 4 | 10 |
| Ssc-let-7c | 67 | 6 | 0 | 17 | 4 | 15 | 15 | 11 | 5 |
| Hsa-miR-652-3p | 64 | 4 | 9 | 8 | 16 | 8 | 8 | 13 | 2 |
| Ssc-miR-181a | 59 | 5 | 2 | 4 | 12 | 24 | 7 | 3 | 7 |
| Ssc-miR-451 | 57 | 2 | 4 | 7 | 12 | 8 | 11 | 4 | 11 |
| Ssc-miR-450c-5p | 56 | 2 | 4 | 9 | 7 | 12 | 12 | 3 | 9 |
| Hsa-miR-214-5p | 55 | 2 | 2 | 5 | 6 | 16 | 12 | 9 | 5 |
| Hsa-let-7d-3p | 55 | 7 | 5 | 7 | 5 | 10 | 3 | 10 | 15 |
| Ssc-miR-19b | 52 | 6 | 6 | 12 | 4 | 12 | 11 | 4 | 3 |
| Dre-miR-20b | 50 | 2 | 2 | 6 | 7 | 11 | 5 | 17 | 2 |
| Ssc-miR-16 | 49 | 4 | 0 | 4 | 2 | 20 | 17 | 5 | 1 |
| Hsa-miR-140-5p | 48 | 4 | 6 | 5 | 5 | 8 | 6 | 11 | 7 |
| Ssc-miR-363 | 46 | 4 | 0 | 9 | 0 | 13 | 7 | 13 | 4 |
| Hsa-miR-200a-5p | 45 | 4 | 0 | 7 | 7 | 11 | 7 | 9 | 4 |
| Hsa-miR-146a-5p | 43 | 7 | 1 | 13 | 9 | 7 | 3 | 4 | 6 |
| Ssc-miR-30c | 41 | 4 | 1 | 7 | 2 | 15 | 7 | 4 | 5 |
| Ssc-miR-15b | 40 | 2 | 1 | 4 | 6 | 11 | 5 | 4 | 9 |
| Ssc-miR-450a | 39 | 7 | 3 | 4 | 0 | 9 | 16 | 6 | 1 |
| Hsa-miR-20a-5p | 38 | 1 | 1 | 10 | 2 | 6 | 11 | 6 | 2 |
| Ssc-miR-27b | 38 | 1 | 0 | 9 | 0 | 10 | 8 | 8 | 3 |
| Hsa-miR-455-5p | 36 | 1 | 0 | 6 | 5 | 9 | 11 | 3 | 2 |
| Hsa-miR-93-5p | 34 | 3 | 1 | 5 | 8 | 5 | 5 | 5 | 5 |
| Ssc-miR-503 | 32 | 4 | 1 | 3 | 2 | 4 | 13 | 2 | 7 |
| Ssc-miR-320 | 31 | 5 | 2 | 15 | 2 | 2 | 3 | 3 | 4 |
| Ssc-miR-152 | 30 | 2 | 3 | 1 | 5 | 8 | 6 | 4 | 3 |
| Hsa-miR-221-3p | 29 | 3 | 3 | 4 | 6 | 4 | 1 | 10 | 1 |
| Ssc-miR-18a | 28 | 3 | 1 | 3 | 0 | 6 | 12 | 3 | 3 |
| Ssc-miR-181c | 27 | 3 | 2 | 6 | 4 | 5 | 5 | 0 | 5 |
| Hsa-miR-193b-5p | 27 | 2 | 4 | 4 | 3 | 5 | 2 | 4 | 5 |
| Hsa-miR-192-3p | 26 | 3 | 1 | 5 | 1 | 10 | 5 | 2 | 2 |
| Ssc-miR-199a* | 25 | 5 | 1 | 5 | 0 | 9 | 2 | 2 | 6 |
| Ssc-miR-28-3p | 25 | 5 | 0 | 3 | 6 | 8 | 0 | 8 | 0 |
| Ssc-miR-29c | 23 | 3 | 0 | 3 | 4 | 8 | 6 | 0 | 2 |
| Ssc-miR-486 | 23 | 4 | 0 | 2 | 3 | 5 | 10 | 0 | 3 |
| Hsa-miR-542-5p | 23 | 2 | 0 | 2 | 8 | 4 | 2 | 1 | 6 |
| Hsa-let-7i-5p | 22 | 4 | 0 | 0 | 3 | 3 | 5 | 11 | 0 |
| Rno-miR-125b* | 22 | 4 | 2 | 2 | 5 | 3 | 3 | 7 | 0 |
| Ssc-miR-424 | 22 | 3 | 0 | 0 | 3 | 9 | 2 | 7 | 1 |
| Ssc-miR-199a-3p | 21 | 3 | 1 | 2 | 9 | 3 | 3 | 0 | 3 |
| Ssc-miR-29a | 21 | 4 | 0 | 2 | 2 | 8 | 6 | 0 | 3 |
| Bta-miR-1468 | 20 | 2 | 1 | 0 | 2 | 4 | 8 | 4 | 1 |
| Hsa-miR-505-3p | 20 | 4 | 3 | 2 | 2 | 4 | 4 | 3 | 2 |
| Ssc-miR-32 | 19 | 1 | 0 | 3 | 4 | 7 | 3 | 1 | 1 |
| Ssc-miR-676-3p | 18 | 2 | 1 | 1 | 3 | 1 | 2 | 2 | 8 |
| Ssc-let-7f | 17 | 1 | 0 | 1 | 0 | 3 | 2 | 10 | 1 |
| Ssc-miR-30b-5p | 17 | 2 | 0 | 5 | 0 | 2 | 5 | 3 | 2 |
| Ssc-miR-425-3p | 16 | 3 | 0 | 9 | 0 | 1 | 6 | 0 | 0 |
| Hsa-miR-4454 | 16 | 1 | 0 | 3 | 8 | 3 | 1 | 1 | 0 |
| Rno-miR-551b | 16 | 1 | 3 | 2 | 2 | 2 | 0 | 2 | 5 |
| Ssc-miR-769-5p | 14 | 3 | 2 | 5 | 1 | 2 | 1 | 2 | 1 |
| Ssc-miR-140* | 13 | 2 | 0 | 1 | 1 | 4 | 3 | 4 | 0 |
| Ssc-miR-151-5p | 13 | 1 | 1 | 2 | 7 | 1 | 0 | 1 | 1 |
| Ssc-let-7e | 12 | 2 | 0 | 2 | 3 | 3 | 2 | 2 | 0 |
| Ssc-miR-128 | 12 | 2 | 1 | 5 | 2 | 0 | 2 | 2 | 0 |
| Hsa-miR-194-5p | 12 | 2 | 2 | 2 | 1 | 5 | 1 | 1 | 0 |
| Ssc-miR-328 | 12 | 2 | 0 | 1 | 4 | 4 | 1 | 1 | 1 |
| Ssc-miR-361-5p | 11 | 1 | 0 | 2 | 1 | 5 | 1 | 0 | 2 |
| Ssc-miR-143-3p | 10 | 1 | 0 | 0 | 0 | 1 | 5 | 4 | 0 |
| Ssc-miR-199a | 10 | 2 | 1 | 0 | 1 | 1 | 2 | 5 | 0 |
| Hsa-miR-505-5p | 10 | 2 | 1 | 0 | 1 | 5 | 1 | 2 | 0 |
| Hsa-miR-106b-3p | 9 | 1 | 3 | 0 | 2 | 2 | 1 | 0 | 1 |
| Ssc-miR-130a | 9 | 2 | 0 | 1 | 0 | 4 | 3 | 0 | 1 |
| Ssc-miR-196b-5p | 9 | 1 | 0 | 4 | 1 | 0 | 1 | 1 | 2 |
| Ssc-miR-34a | 9 | 1 | 0 | 0 | 2 | 1 | 2 | 3 | 1 |
| Ssc-miR-129a | 8 | 2 | 0 | 2 | 1 | 3 | 0 | 1 | 1 |
| Ssc-miR-27a | 8 | 1 | 0 | 0 | 3 | 4 | 1 | 0 | 0 |
| Ssc-miR-30e-3p | 8 | 2 | 2 | 1 | 0 | 3 | 0 | 2 | 0 |
| Ssc-miR-331-5p | 8 | 2 | 0 | 0 | 0 | 1 | 0 | 5 | 2 |
| Hsa-miR-335-3p | 8 | 2 | 0 | 1 | 1 | 0 | 2 | 3 | 1 |
| Hsa-miR-378a-5p | 8 | 1 | 1 | 0 | 2 | 1 | 0 | 3 | 1 |
| Bta-miR-423-3p | 8 | 1 | 1 | 0 | 0 | 0 | 2 | 0 | 5 |
| Hsa-miR-4286 | 8 | 2 | 0 | 2 | 2 | 1 | 3 | 0 | 0 |
| Hsa-miR-551a | 8 | 1 | 1 | 1 | 0 | 1 | 5 | 0 | 0 |
| Mdo-miR-106 | 7 | 1 | 0 | 1 | 0 | 2 | 4 | 0 | 0 |
| Ssc-miR-195 | 7 | 2 | 0 | 0 | 2 | 2 | 1 | 2 | 0 |
| Ssc-miR-199b* | 7 | 2 | 0 | 1 | 0 | 1 | 1 | 4 | 0 |
| Ssc-miR-339-5p | 7 | 2 | 0 | 0 | 4 | 0 | 0 | 3 | 0 |
| Hsa-miR-9-3p | 7 | 2 | 0 | 0 | 0 | 2 | 4 | 1 | 0 |
| Hsa-let-7b-3p | 6 | 1 | 1 | 2 | 0 | 0 | 0 | 3 | 0 |
| Hsa-miR-125a-3p | 6 | 1 | 0 | 0 | 1 | 1 | 2 | 2 | 0 |
| Ssc-miR-17-5p | 6 | 1 | 0 | 0 | 1 | 1 | 2 | 2 | 0 |
| Ssc-miR-20 | 6 | 2 | 0 | 0 | 0 | 2 | 1 | 3 | 0 |
| Bta-miR-2483 | 6 | 1 | 0 | 1 | 0 | 0 | 4 | 1 | 0 |
| Ssc-miR-92b-3p | 6 | 1 | 0 | 1 | 1 | 3 | 0 | 1 | 0 |
| Ssc-miR-130b | 5 | 1 | 0 | 1 | 0 | 2 | 0 | 1 | 1 |
| Ssc-miR-181b | 5 | 1 | 0 | 2 | 0 | 1 | 1 | 0 | 1 |
| Mmu-miR-29b-2-5p | 5 | 1 | 1 | 1 | 1 | 1 | 0 | 0 | 1 |
| Ssc-miR-4334-3p | 5 | 1 | 0 | 0 | 3 | 1 | 1 | 0 | 0 |
| Eca-miR-545 | 5 | 1 | 0 | 0 | 0 | 2 | 3 | 0 | 0 |
| Sha-miR-716b | 5 | 1 | 0 | 4 | 1 | 0 | 0 | 0 | 0 |
| Hsa-miR-138-5p | 4 | 1 | 0 | 0 | 0 | 0 | 2 | 0 | 2 |
| Ssc-miR-183 | 4 | 1 | 0 | 1 | 2 | 1 | 0 | 0 | 0 |
| Hsa-miR-18a-3p | 4 | 1 | 0 | 0 | 0 | 1 | 1 | 0 | 2 |
| Ssc-miR-193a-3p | 4 | 1 | 2 | 2 | 0 | 0 | 0 | 0 | 0 |
| Hsa-miR-31-3p | 4 | 1 | 0 | 4 | 0 | 0 | 0 | 0 | 0 |
| Ssc-miR-340 | 4 | 1 | 0 | 1 | 0 | 0 | 3 | 0 | 0 |
| Ssc-miR-345-3p | 4 | 1 | 2 | 0 | 2 | 0 | 0 | 0 | 0 |
| Ssc-miR-424* | 4 | 1 | 0 | 4 | 0 | 0 | 0 | 0 | 0 |
| Ssc-miR-542-3p | 4 | 1 | 1 | 0 | 2 | 0 | 0 | 0 | 1 |
| Ssc-miR-574 | 4 | 1 | 0 | 0 | 1 | 1 | 2 | 0 | 0 |
| Ssc-miR-9-1 | 4 | 1 | 0 | 3 | 0 | 1 | 0 | 0 | 0 |
| Hsa-miR-99a-3p | 4 | 1 | 0 | 0 | 0 | 3 | 1 | 0 | 0 |
| Hsa-let-7e-3p | 3 | 1 | 0 | 0 | 0 | 0 | 0 | 3 | 0 |
| Ssc-let-7g | 3 | 1 | 0 | 0 | 0 | 1 | 1 | 1 | 0 |
| Hsa-miR-1271-3p | 3 | 1 | 0 | 0 | 1 | 0 | 2 | 0 | 0 |
| Ssc-miR-1343 | 3 | 1 | 0 | 0 | 0 | 0 | 0 | 2 | 1 |
| Hsa-miR-200b-5p | 3 | 1 | 0 | 3 | 0 | 0 | 0 | 0 | 0 |
| Hsa-miR-28-5p | 3 | 1 | 0 | 0 | 3 | 0 | 0 | 0 | 0 |
| Mmu-miR-2137 | 3 | 1 | 0 | 1 | 0 | 2 | 0 | 0 | 0 |
| Ssc-miR-345-5p | 3 | 1 | 0 | 0 | 1 | 0 | 1 | 0 | 1 |
| Ssc-miR-361-3p | 3 | 1 | 0 | 1 | 1 | 0 | 0 | 1 | 0 |
| Mmu-miR-5100 | 3 | 1 | 0 | 2 | 0 | 1 | 0 | 0 | 0 |

IB: Iberian breed, WB: European Wild Boar, LD: Landrace breed, LW: Large White breed, PT: Piétrain breed, ME: Meishan breed, VT: Vietnamese breed.
miRNA name represents the most expressed sequence in the cluster.
Bta: *Bos taurus*, Dre: *Danio rerio*, Eca: *Equus caballus,* Hsa: *Homo sapiens*, Mdo: *Monodelphis domestica*, Mmu: *Mus musculus*, Rno: *Ratus norvegicus*, Sha: *Sarcophilus harrisii*, Ssc: *Sus scrofa*.
