# Supplementary material for: miRNA Expression Profile Analysis in Kidney of Different Porcine Breeds
Source: PLoS One. 2013 Jan 25;8(1):e55402. doi: 10.1371/journal.pone.0055402 (PMC3555835; doi:10.1371/journal.pone.0055402)
Supplement: Table S3 — Summary of the isomiR distribution in the most expressed miRNAs (CN>1,000). miRNA name represents the most expressed sequence in the cluster. Hsa: Homo sapiens, Ssc: Sus scrofa. (DOC) [file pone.0055402.s003.doc]

**Table S3. Summary of the isomiR distribution in the most expressed miRNAs (CN>1,000)**.

| **miRNA name** | **Total counts** | **IsomiRs** | **Counts of the most expressed isomiR (%)** | **Counts of the second most expressed isomiR (%)** | **Counts of the third most expressed isomiR (%)** |
| --- | --- | --- | --- | --- | --- |
| Hsa-miR-200b-3p | 27,097 | 123 | 12,988 (48%) | 4,127 (15%) | 3,180 (12%) |
| Ssc-miR-125b | 8,809 | 51 | 6,308 (72%) | 578 (7%) | 447 (5%) |
| Ssc-miR-23b | 5,412 | 59 | 2,760 (51%) | 740 (14%) | 511 (9%) |
| Ssc-miR-126 | 5,274 | 49 | 1,958 (37%) | 1,083 (21%) | 629 (12%) |
| Ssc-miR-23a | 5,156 | 51 | 3,116 (60%) | 622 (12%) | 284 (6%) |
| Ssc-miR-192 | 3,863 | 41 | 1,875 (49%) | 841 (22%) | 257 (7%) |
| Ssc-miR-99a | 3,781 | 32 | 1,124 (30%) | 1,085 (29%) | 912 (24%) |
| Hsa-miR-200c-3p | 3,478 | 32 | 1,711 (49%) | 1,102 (32%) | 189 (5%) |
| Ssc-miR-10b | 2,846 | 31 | 1,731 (61%) | 329 (12%) | 124 (4%) |
| Ssc-miR-126* | 2,796 | 26 | 1,326 (47%) | 749 (27%) | 313 (11%) |
| Ssc-miR-30d | 1,977 | 31 | 711 (36%) | 334 (17%) | 190 (10%) |
| Ssc-miR-125a | 1,369 | 29 | 479 (35%) | 326 (24%) | 218 (16%) |
| Ssc-miR-10a | 1,317 | 19 | 657 (50%) | 249 (19%) | 91 (7%) |

miRNA name represents the most expressed sequence in the cluster.
Hsa: *Homo sapiens*, Ssc: *Sus scrofa*.
