# Supplementary material for: miRNA Expression Profile Analysis in Kidney of Different Porcine Breeds
Source: PLoS One. 2013 Jan 25;8(1):e55402. doi: 10.1371/journal.pone.0055402 (PMC3555835; doi:10.1371/journal.pone.0055402)
Supplement: Table S4 — Described miRNAs from HTS presenting variation between the reference miRNA sequence and the miRBase described miRNA sequence. Bta: Bos taurus, Eca: Ecuus caballus, Hsa: Homo sapiens, Mdo: Monodelphis domestica, Mmu: Mus musculus, Rno: Rattus norvegicus, Sha: Sarcophilus harrisii, Ssc: Sus scrofa. Marked in bold the nucleotide variation between pair sequences. (DOC) [file pone.0055402.s004.doc]

**Table S4. Described miRNAs from HTS presenting variation between the reference miRNA sequence and the miRBase described miRNA sequence.**

| **miRNA name** | **Total counts** | **IsomiRs** | **Most expressed isomiR sequence (5’-3’)** | **Counts** | **Described miRNA sequence in miRBase database (5’-3’)** | **Counts** |
| --- | --- | --- | --- | --- | --- | --- |
| Ssc-miR-23a | 5,156 | 51 | ATCACATTGCCAGGGATTTCC**A** | 3,116 | ATCACATTGCCAGGGATTTCC | 622 |
| Ssc-miR-99a | 3,781 | 32 | AACCCGTAGATCCGATCTTGTG**A** | 1,124 | AACCCGTAGATCCGATCTTGTG | 1,085 |
| Ssc-miR-30d | 1,977 | 31 | TGTAAACATCCCCGACTGGAAGC | 711 | TGTAAACATCCCCGACTGGAAGC**T** | 190 |
| Ssc-miR-125a | 1,369 | 29 | TCCCTGAGACCCTTTAACCTGT | 479 | TCCCTGAGACCCTTTAACCTGT**G** | 326 |
| Bta-miR-193b | 473 | 26 | AACTGGCCCACAAAGTCCCGCT | 196 | AACTGGCCCACAAAGTCCCGCT**TT** | 21 |
| Ssc-miR-30e-5p | 461 | 14 | TGTAAACATCCTTGACTGGAAGC | 268 | TGTAAACATCCTTGACTGGAAGC**T** | 74 |
| Ssc-miR-139-5p | 329 | 8 | TCTACAGTGCACGTGTCTCCAG**T** | 216 | TCTACAGTGCACGTGTCTCCAG | 85 |
| Ssc-miR-30a-5p | 314 | 10 | TGTAAACATCCTCGACTGGAAGC | 198 | TGTAAACATCCTCGACTGGAAGC**T** | 15 |
| Ssc-miR-362 | 303 | 13 | AATCCTTGGAACCTAGGTGTGAGT | 133 | AATCCTTGGAACCTAGGTGTGAGT**G** | 0 |
| Hsa-miR-29c-5p | 260 | 20 | GACCGATTTCTCCTGGTGTTC**A** | 37 | **T**GACCGATTTCTCCTGGTGTTC | 22 |
| Ssc-miR-374a | 253 | 6 | TTATAATACAACCTGATAAGTG**T** | 117 | TTATAATACAACCTGATAAGTG | 13 |
| Ssc-miR-145 | 222 | 12 | GTCCAGTTTTCCCAGGAATCCCT | 91 | GTCCAGTTTTCCCAGGAATCCCT**T** | 3 |
| Hsa-miR-324-3p | 211 | 22 | ACTGCCCCAGGTGCTGCTGG**T** | 45 | ACTGCCCCAGGTGCTGCTGG | 0 |
| Ssc-miR-21 | 210 | 4 | TAGCTTATCAGACTGATGTTGA**C** | 105 | TAGCTTATCAGACTGATGTTGA | 77 |
| Hsa-miR-874 | 195 | 8 | CTGCCCTGGCCCGAGGGACCGA**C** | 56 | CTGCCCTGGCCCGAGGGACCGA | 42 |
| Ssc-miR-218b | 185 | 7 | TTGTGCTTGATCTAACCATGT | 56 | TTGTGCTTGATCTAACCATGT**G** | 27 |
| Ssc-miR-191 | 183 | 8 | CAACGGAATCCCAAAAGCAGCT | 63 | CAACGGAATCCCAAAAGCAGCT**G** | 45 |
| Hsa-miR-150-5p | 174 | 12 | **GT**TCTCCCAACCCTTGTACCAGT | 47 | TCTCCCAACCCTTGTACCAGT**G** | 14 |
| Ssc-miR-22-3p | 106 | 5 | AAGCTGCCAGTTGAAGAAC | 43 | AAGCTGCCAGTTGAAGAAC**TGT** | 28 |
| Ssc-miR-450b-5p | 97 | 7 | TTTTGCAATATGTTCCTGAAT | 52 | TTTTGCAATATGTTCCTGAAT**A** | 6 |
| Ssc-miR-664-5p | 89 | 6 | CAGGCTAGGAGAAGTGATTGGA | 52 | CAGGCTAGGAGAAGTGATTGGA**T** | 15 |
| Ssc-miR-374b-5p | 86 | 5 | ATATAATACAACCTGCTAAGTG**T** | 64 | ATATAATACAACCTGCTAAGTG | 5 |
| Ssc-miR-29b | 75 | 5 | TAGCACCATTTGAAATCAGTGT | 46 | TAGCACCATTTGAAATCAGTGT**T** | 0 |
| Hsa-miR-29a-5p | 75 | 5 | ACTGATTTCTTTTGGTGTTCAG**A** | 28 | ACTGATTTCTTTTGGTGTTCAG | 20 |
| Ssc-miR-532-3p | 75 | 6 | **GT**CCTCCCACACCCAAGGCTTGCA | 23 | CCTCCCACACCCAAGGCTTGCA | 11 |
| Ssc-miR-24 | 74 | 7 | TGGCTCAGTTCAGCAGGAACAG**T** | 20 | TGGCTCAGTTCAGCAGGAACAG | 16 |
| Ssc-miR-423-5p | 74 | 10 | TGAGGGGCAGAGAGCGAGA**AA** | 15 | TGAGGGGCAGAGAGCGAGA**CTTT** | 0 |
| Ssc-miR-186 | 73 | 6 | CAAAGAATTCTCCTTTTGGGCT | 42 | CAAAGAATTCTCCTTTTGGGCT**T** | 5 |
| Ssc-miR-500 | 68 | 8 | ATGCACCTGGGCAAGGATTCTG**A** | 35 | ATGCACCTGGGCAAGGATTCTG | 4 |
| Hsa-miR-652-3p | 64 | 4 | AATGGCGCCACTAGGGTTGTG**T** | 45 | AATGGCGCCACTAGGGTTGTG | 12 |
| Ssc-miR-181a | 59 | 5 | AACATTCAACGCTGTCGGTGAGT | 23 | AACATTCAACGCTGTCGGTGAGT**T** | 0 |
| Ssc-miR-451 | 57 | 2 | AAACCGTTACCATTACTGAGTT**T** | 30 | AAACCGTTACCATTACTGAGTT | 27 |
| Ssc-miR-19b | 52 | 6 | TGTGCAAATCCATGCAAAACTG | 20 | TGTGCAAATCCATGCAAAACTG**A** | 13 |
| Hsa-miR-140-5p | 48 | 4 | CAGTGGTTTTACCCTATGGTAG**T** | 21 | CAGTGGTTTTACCCTATGGTAG | 3 |
| Hsa-miR-146a-5p | 43 | 7 | TGAGAACTGAATTCCATGGGTT**A** | 11 | TGAGAACTGAATTCCATGGGTT | 7 |
| Ssc-miR-15b | 40 | 2 | TAGCAGCACATCATGGTTTAC | 34 | TAGCAGCACATCATGGTTTAC**A** | 6 |
| Ssc-miR-503 | 32 | 4 | TAGCAGCGGGAACAGTACTGCA | 22 | TAGCAGCGGGAACAGTACTGCA**G** | 0 |
| Hsa-miR-221-3p | 29 | 3 | AGCTACATTGTCTGCTGGGTTT**C** | 18 | AGCTACATTGTCTGCTGGGTTT | 6 |
| Ssc-miR-18a | 28 | 3 | TAAGGTGCATCTAGTGCAGAT | 12 | TAAGGTGCATCTAGTGCAGAT**A** | 5 |
| Hsa-miR-192-3p | 26 | 3 | CTGCCAATTCCATAGGTCACAG**T** | 11 | CTGCCAATTCCATAGGTCACAG | 10 |
| Ssc-miR-199a* | 25 | 5 | AGTAGTCTGCACATTGGTTA**A** | 7 | **C**AGTAGTCTGCACATTGGTTA | 0 |
| Ssc-miR-28-3p | 25 | 5 | CACTAGATTGTGAGCTCCTGGA**A** | 8 | CACTAGATTGTGAGCTCCTGGA | 6 |
| Hsa-let-7i-5p | 22 | 4 | TGAGGTAGTAGTTTGTGCTGTT**T** | 8 | TGAGGTAGTAGTTTGTGCTGTT | 6 |
| Ssc-miR-424 | 22 | 3 | CAGCAGCAATTCATGTTTTGA | 10 | CAGCAGCAATTCATGTTTTGA**A** | 8 |
| Ssc-miR-199a-3p | 21 | 3 | ACAGTAGTCTGCACATTGGTT | 14 | ACAGTAGTCTGCACATTGGTT**A** | 4 |
| Ssc-miR-29a | 21 | 4 | TAGCACCATCTGAAATCGGTTA | 7 | **C**TAGCACCATCTGAAATCGGTTA | 0 |
| Hsa-miR-505-3p | 20 | 4 | GTCAACACTTGCTGGTTTCCT | 9 | **C**GTCAACACTTGCTGGTTTCCT | 4 |
| Ssc-miR-676-3p | 18 | 2 | CCGTCCTAAGGTTGTTGAGTT**T** | 12 | CCGTCCTAAGGTTGTTGAGTT | 6 |
| Ssc-miR-425-3p | 16 | 3 | **C**ATCGGGAATGTCGTGTCCGCC | 7 | ATCGGGAATGTCGTGTCCGCC**C** | 0 |
| Hsa-miR-4454 | 16 | 1 | TCGAATCCGAGTCACGGCACCA | 16 | GGATCCGAGTCACGGCACCA | 0 |
| Rno-miR-551b | 16 | 1 | GCGACCCATACTTGGTTTCAGT | 16 | **G**GCGACCCATACTTGGTTTCAGT | 0 |
| Ssc-miR-769-5p | 14 | 3 | TGAGACCTCTGGGTTCTGAG**A** | 7 | TGAGACCTCTGGGTTCTGAG**C** | 4 |
| Ssc-miR-199a | 10 | 2 | CCCAGTGTTCAGACTACCTGTT | 7 | CCCAGTGTTCAGACTACCTGTT**C** | 3 |
| Ssc-miR-196b-5p | 9 | 1 | TAGGTAGTTTCCTGTTGTTGG | 9 | TAGGTAGTTTCCTGTTGTTGG**G** | 0 |
| Hsa-miR-335-3p | 8 | 2 | **T**TTTTTCATTATTGCTCCTGACC | 5 | TTTTTCATTATTGCTCCTGACC | 3 |
| Hsa-miR-4286 | 8 | 2 | ACCCCACTCCTGGTACC**AAAA** | 5 | ACCCCACTCCTGGTACC | 0 |
| Mdo-miR-106 | 7 | 1 | **T**AAAAGTGCTTATAGTGCAGGTAG | 7 | AAAAGTGCTTATAGTGCAGGTAG | 0 |
| Ssc-miR-199b* | 7 | 2 | TACAGTAGTCTGCACATTGGT | 4 | TACAGTAGTCTGCACATTGGT**T** | 3 |
| Hsa-let-7b-3p | 6 | 1 | CTATACAACCTACTGCCTTCC**T** | 6 | CTATACAACCTACTGCCTTCC**C** | 0 |
| Ssc-miR-181b | 5 | 1 | AACATTCATTGCTGTCGGTG**T** | 5 | AACATTCATTGCTGTCGGTG**GGTT** | 0 |
| Mmu-miR-29b-2-5p | 5 | 1 | CTGGTTTCACATGGTGGCTTAGA | 5 | CTGGTTTCACATGGTGGCTTAGA**TT** | 0 |
| Eca-miR-545 | 5 | 1 | TCAACAAACATTTATTGTGTGC**C** | 5 | **A**TCAACAAACATTTATTGTGTGC | 0 |
| Sha-miR-716b | 5 | 1 | **GC**AGATCTTGGTGGTAGTAGCA | 5 | AGATCTTGGTGGTAGTAGCA**AATAT** | 0 |
| Ssc-miR-183 | 4 | 1 | TATGGCACTGGTAGAATTCACT | 4 | TATGGCACTGGTAGAATTCACT**G** | 0 |
| Hsa-miR-18a-3p | 4 | 1 | ACTGCCCTAAGTGCTCCTTCTG | 4 | ACTGCCCTAAGTGCTCCTTCTG**G** | 0 |
| Hsa-miR-31-3p | 4 | 1 | TGCTATGCCAACATATTGCCA | 4 | TGCTATGCCAACATATTGCCA**T** | 0 |
| Ssc-miR-345-3p | 4 | 1 | CCTGAACTAGGGGTCTGGAG**T** | 4 | **C**CCTGAACTAGGGGTCTGGAG | 0 |
| Ssc-miR-9-1 | 4 | 1 | TCTTTGGTTATCTAGCTGTAT | 4 | TCTTTGGTTATCTAGCTGTAT**GA** | 0 |
| Hsa-miR-99a-3p | 4 | 1 | CAAGCTCGCTTCTATGGGTCTG**T** | 4 | CAAGCTCGCTTCTATGGGTCTG | 0 |
| Hsa-miR-1271-3p | 3 | 1 | AGTGCCTGCTATGTGCCAG**A** | 3 | AGTGCCTGCTATGTGCCAG**GCA** | 0 |
| Mmu-miR-2137 | 3 | 1 | CGGCGGGAGCCCC**GGGGAGA** | 3 | **GC**CGGCGGGAGCCCC**AGGGAG** | 0 |
| Mmu-miR-5100 | 3 | 1 | TCGAATCCCAGCGGTGCCTC**CA** | 3 | TCGAATCCCAGCGGTGCCTC**T** | 0 |

Bta: *Bos taurus*, Eca: *Ecuus caballus,* Hsa: *Homo sapiens*, Mdo: *Monodelphis domestica*, Mmu: *Mus musculus*, Rno: *Rattus norvegicus*, Sha: *Sarcophilus harrisii,* Ssc: *Sus scrofa*.
Marked in bold the nucleotide variation between pair sequences.
