# Supplementary material for: miRNA Expression Profile Analysis in Kidney of Different Porcine Breeds
Source: PLoS One. 2013 Jan 25;8(1):e55402. doi: 10.1371/journal.pone.0055402 (PMC3555835; doi:10.1371/journal.pone.0055402)
Supplement: Table S5 — List of differentially expressed miRNAs (Fold Change >1.5 times) between porcine breed groups. EU: European breeds; EA: European commercial breeds; AS: Asian breeds. Bta: Bos taurus, Dre: Danio rerio, Eca: Equus caballus, Hsa: Homo sapiens, Mdo: Monodelphis domestica, Mmu: Mus musculus, Rno: Ratus norvegicus, Sha: Sarcophilus harrisii, Ssc: Sus scrofa. Positive and negative signs indicate that the level of gene expression is higher for the first or the second group of the test, respectively. (DOC) [file pone.0055402.s005.doc]

**Table S5. List of differentially expressed miRNAs (Fold Change > 1.5 times) between porcine breed groups.**

| **miRNA name** | **Total counts** | **Fold Change EU vs EA** | **Fold Change EU vs AS** | **Fold Change EA vs AS** | **Up regulation** | **Down regulation** |
| --- | --- | --- | --- | --- | --- | --- |
| Hsa-miR-200b-3p | 27,097 | -1.45 | -1.54 | -1.06 | AS | EU |
| Ssc-miR-125b | 8,809 | 1.14 | 2.33 | 2.04 | EU | AS |
| Ssc-miR-99a | 3,781 | 1.63 | 3.05 | 1.87 | EU | AS |
| Hsa-miR-200c-3p | 3,478 | -2.31 | -1.73 | 1.34 | EA | EU |
| Ssc-miR-30d | 1,977 | -1.30 | 1.68 | 2.19 | EA | AS |
| Ssc-miR-125a | 1,369 | 1.31 | 2.23 | 1.70 | EU | AS |
| Ssc-miR-365-3p | 986 | -1.96 | 1.24 | 2.44 | EA | AS |
| Ssc-miR-92a | 797 | -1.87 | -1.68 | 1.11 | EA | EU |
| Ssc-miR-204 | 748 | -3.86 | 1.02 | 3.93 | EA | AS |
| Ssc-miR-378 | 743 | 1.90 | 2.43 | 1.28 | EU | AS |
| Ssc-miR-26a | 598 | -2.15 | -1.29 | 1.67 | EA | EU |
| Bta-miR-193b | 473 | 2.23 | 5.46 | 2.45 | EU | AS |
| Ssc-miR-30e-5p | 461 | 1.10 | 1.61 | 1.46 | EU | AS |
| Ssc-miR-100 | 405 | -1.11 | 1.69 | 1.87 | EA | AS |
| Ssc-miR-99b | 350 | -1.24 | 1.53 | 1.90 | EA | AS |
| Ssc-miR-139-5p | 329 | -1.07 | 1.42 | 1.53 | EA | AS |
| Ssc-miR-30a-5p | 314 | -1.02 | 1.66 | 1.70 | EA | AS |
| Ssc-miR-324 | 314 | -1.54 | 2.27 | 3.50 | EA | AS |
| Ssc-miR-362 | 303 | -3.08 | -1.38 | 2.24 | EA | EU |
| Ssc-miR-429 | 287 | -1.81 | -1.23 | 1.47 | EA | EU |
| Ssc-miR-374a | 253 | -2.47 | -2.36 | 1.05 | EA | EU |
| Hsa-miR-500a-5p | 246 | -1.92 | -1.14 | 1.69 | EA | EU |
| Ssc-miR-145 | 222 | 1.28 | 2.35 | 1.84 | EU | AS |
| Hsa-miR-324-3p | 211 | 1.67 | 2.04 | 1.22 | EU | AS |
| Ssc-miR-21 | 210 | -2.12 | -1.06 | 2.00 | EA | EU |
| Hsa-miR-25-3p | 198 | -1.15 | 1.81 | 2.07 | EA | AS |
| Hsa-miR-874 | 195 | -1.36 | 1.78 | 2.42 | EA | AS |
| Ssc-miR-191 | 183 | 1.63 | 2.54 | 1.56 | EU | AS |
| Hsa-miR-150-5p | 174 | -1.63 | 1.20 | 1.96 | EA | AS |
| Ssc-miR-193a-5p | 148 | 3.47 | 2.72 | -1.27 | EU | EA |
| Ssc-let-7a | 125 | -1.57 | -2.26 | -1.44 | AS | EU |
| Ssc-miR-532-5p | 113 | -1.16 | -2.49 | -2.14 | AS | EU |
| Ssc-miR-22-3p | 106 | 1.54 | 2.37 | 1.54 | EU | AS |
| Ssc-miR-450b-5p | 97 | -1.47 | -2.09 | -1.43 | AS | EU |
| Ssc-miR-664-5p | 89 | -1.13 | 1.46 | 1.65 | EA | AS |
| Ssc-miR-374b-5p | 86 | -3.83 | -1.54 | 2.48 | EA | EU |
| Hsa-let-7b-5p | 77 | -1.84 | -1.48 | 1.24 | EA | EU |
| Ssc-miR-151-3p | 75 | 1.40 | 2.40 | 1.71 | EU | AS |
| Hsa-miR-29a-5p | 75 | -1.08 | 2.37 | 2.56 | EA | AS |
| Ssc-miR-29b | 75 | 1.46 | 3.21 | 2.20 | EU | AS |
| Ssc-miR-532-3p | 75 | 1.49 | 2.06 | 1.39 | EU | AS |
| Ssc-miR-24 | 74 | 2.21 | 3.35 | 1.52 | EU | AS |
| Ssc-miR-423-5p | 74 | 5.28 | 3.76 | -1.40 | EU | EA |
| Hsa-let-7d-5p | 68 | 2.35 | 1.14 | -2.06 | EU | EA |
| Ssc-miR-500 | 68 | 1.44 | 2.04 | 1.42 | EU | AS |
| Hsa-miR-652-3p | 64 | 2.11 | 3.11 | 1.47 | EU | AS |
| Ssc-miR-181a | 59 | -2.08 | 1.26 | 2.62 | EA | AS |
| Ssc-miR-451 | 57 | 1.17 | 1.62 | 1.38 | EU | AS |
| Ssc-miR-450c-5p | 56 | 1.38 | 2.18 | 1.58 | EU | AS |
| Hsa-let-7d-3p | 55 | 2.61 | 1.14 | -2.28 | EU | EA |
| Ssc-miR-19b | 52 | 2.40 | 5.38 | 2.24 | EU | AS |
| Ssc-miR-16 | 49 | -5.60 | -1.58 | 3.54 | EA | EU |
| Hsa-miR-140-5p | 48 | 2.65 | 1.72 | -1.54 | EU | EA |
| Ssc-miR-363 | 46 | -1.22 | -1.99 | -1.63 | AS | EU |
| Hsa-miR-200a-5p | 45 | -2.31 | -1.95 | 1.18 | EA | EU |
| Hsa-miR-146a-5p | 43 | 1.30 | 1.68 | 1.29 | EU | AS |
| Ssc-miR-15b | 40 | -1.64 | -1.58 | 1.04 | EA | EU |
| Ssc-miR-450a | 39 | 1.24 | 2.40 | 1.94 | EU | AS |
| Hsa-miR-20a-5p | 38 | 1.26 | 1.73 | 1.37 | EU | AS |
| Hsa-miR-455-5p | 36 | -2.63 | 1.14 | 3.01 | EA | AS |
| Ssc-miR-503 | 32 | -1.55 | -1.24 | 1.25 | EA | EU |
| Ssc-miR-320 | 31 | 5.21 | 3.30 | -1.58 | EU | EA |
| Ssc-miR-152 | 30 | 1.20 | 2.01 | 1.67 | EU | AS |
| Hsa-miR-221-3p | 29 | 2.21 | 1.53 | -1.44 | EU | EA |
| Ssc-miR-18a | 28 | -1.37 | 1.21 | 1.65 | EA | AS |
| Ssc-miR-181c | 27 | 1.65 | 2.92 | 1.77 | EU | AS |
| Hsa-miR-193b-5p | 27 | 3.44 | 2.37 | -1.45 | EU | EA |
| Hsa-miR-192-3p | 26 | 1.05 | 2.29 | 2.17 | EU | AS |
| Ssc-miR-199a* | 25 | 1.64 | 1.15 | -1.42 | EU | EA |
| Ssc-miR-28-3p | 25 | -3.16 | -2.82 | 1.12 | EA | EU |
| Ssc-miR-29c | 23 | -3.78 | 1.45 | 5.47 | EA | AS |
| Ssc-miR-486 | 23 | -5.66 | -1.55 | 3.64 | EA | EU |
| Hsa-miR-542-5p | 23 | -5.21 | -3.64 | 1.43 | EA | EU |
| Hsa-let-7i-5p | 22 | - | - | -1.60 | - | - |
| Rno-miR-125b* | 22 | 1.42 | 1.51 | 1.06 | EU | AS |
| Ssc-miR-199a-3p | 21 | -1.74 | 2.11 | 3.67 | EA | AS |
| Ssc-miR-29a | 21 | -4.77 | -1.55 | 3.07 | EA | EU |
| Bta-miR-1468 | 20 | -1.90 | -1.15 | 1.65 | EA | EU |
| Hsa-miR-505-3p | 20 | 2.50 | 3.00 | 1.20 | EU | AS |
| Ssc-miR-32 | 19 | -3.00 | 1.43 | 4.30 | EA | AS |
| Ssc-miR-676-3p | 18 | 1.26 | -1.87 | -2.36 | AS | EA |
| Ssc-let-7f | 17 | -2.76 | -11.60 | -4.21 | AS | EU |
| Ssc-miR-425-3p | 16 | 2.17 | - | - | - | - |
| Hsa-miR-4454 | 16 | -3.10 | 2.84 | 8.81 | EA | AS |
| Rno-miR-551b | 16 | 5.58 | 2.16 | -2.59 | EU | EA |
| Ssc-miR-769-5p | 14 | 5.59 | 4.49 | -1.24 | EU | EA |
| Ssc-miR-140* | 13 | -4.77 | -4.23 | 1.13 | EA | EU |
| Ssc-miR-151-5p | 13 | -1.03 | 3.14 | 3.24 | EA | AS |
| Ssc-let-7e | 12 | -2.72 | -1.06 | 2.58 | EA | EU |
| Ssc-miR-128 | 12 | 3.19 | 4.53 | 1.42 | EU | AS |
| Hsa-miR-194-5p | 12 | 2.73 | 10.55 | 3.86 | EU | AS |
| Ssc-miR-328 | 12 | -6.24 | -2.09 | 2.98 | EA | EU |
| Ssc-miR-361-5p | 11 | -2.04 | -1.04 | 1.97 | EA | EU |
| Ssc-miR-199a | 10 | 1.74 | -1.16 | -2.01 | AS | EA |
| Hsa-miR-505-5p | 10 | 1.12 | 2.16 | 1.93 | EU | AS |
| Hsa-miR-106b-3p | 9 | 4.00 | 13.24 | 3.31 | EU | AS |
| Ssc-miR-130a | 9 | -3.88 | -1.04 | 3.74 | EA | EU |
| Ssc-miR-196b-5p | 9 | 2.66 | 1.28 | -2.08 | EU | EA |
| Ssc-miR-30e-3p | 8 | 6.57 | 4.80 | -1.37 | EU | EA |
| Ssc-miR-331-5p | 8 | - | - | -14.29 | - | - |
| Hsa-miR-335-3p | 8 | -2.11 | -4.21 | -2.00 | AS | EU |
| Hsa-miR-378a-5p | 8 | 1.98 | 1.09 | -1.83 | EU | EA |
| Bta-miR-423-3p | 8 | 3.77 | -1.13 | -4.27 | AS | EA |
| Hsa-miR-4286 | 8 | -2.06 | - | - | - | - |
| Hsa-miR-551a | 8 | 1.57 | - | - | - | - |
| Mdo-miR-106 | 7 | -3.46 | - | - | - | - |
| Ssc-miR-195 | 7 | - | - | 1.62 | - | - |
| Ssc-miR-199b* | 7 | -1.12 | -4.23 | -3.77 | AS | EU |
| Hsa-miR-9-3p | 7 | - | - | 3.27 | - | - |
| Hsa-let-7b-3p | 6 | - | 2.07 | - | - | - |
| Ssc-miR-20 | 6 | - | - | -1.94 | - | - |
| Bta-miR-2483 | 6 | -2.43 | -1.06 | 2.30 | EA | EU |
| Ssc-miR-92b-3p | 6 | -2.44 | -1.06 | 2.31 | EA | EU |
| Ssc-miR-130b | 5 | -1.03 | -2.09 | -2.03 | AS | EU |
| Ssc-miR-181b | 5 | 1.78 | 1.93 | 1.08 | EU | AS |
| Mmu-miR-29b-2-5p | 5 | 3.95 | 5.38 | 1.36 | EU | AS |
| Sha-miR-716b | 5 | 4.47 | - | - | - | - |
| Hsa-miR-138-5p | 4 | - | - | -1.71 | - | - |
| Ssc-miR-183 | 4 | -2.30 | - | - | - | - |
| Hsa-miR-18a-3p | 4 | - | - | -1.85 | - | - |
| Ssc-miR-340 | 4 | -1.82 | - | - | - | - |
| Ssc-miR-345-3p | 4 | 5.11 | - | - | - | - |
| Ssc-miR-542-3p | 4 | 2.56 | 4.41 | 1.73 | EU | AS |
| Ssc-miR-9-1 | 4 | 5.83 | - | - | - | - |
| Mmu-miR-5100 | 3 | 3.88 | - | - | - | - |

EU: European breeds; EA: European commercial breeds; AS: Asian breeds.
Bta: *Bos taurus*, Dre: *Danio rerio*, Eca: *Equus caballus*, Hsa: *Homo sapiens*, Mdo: *Monodelphis domestica*, Mmu: *Mus musculus*, Rno: *Ratus norvegicus*, Sha: *Sarcophilus harrisii*, Ssc: *Sus scrofa*.
Positive and negative signs indicate that the level of gene expression is higher for the first or the second group of the test, respectively.
