# Supplementary material for: miRNA Expression Profile Analysis in Kidney of Different Porcine Breeds
Source: PLoS One. 2013 Jan 25;8(1):e55402. doi: 10.1371/journal.pone.0055402 (PMC3555835; doi:10.1371/journal.pone.0055402)
Supplement: Table S6 — Normalised 454 GS FLX run data by library in counts per thousand. IB: Iberian breed, WB: European Wild Boar, LD: Landrace breed, LW: Large White breed, PT: Piétrain breed, ME: MeiShan breed, VT: Vietnamese breed. miRNA name represents the most expressed sequence in the cluster. Bta: Bos taurus, Dre: Danio rerio, Eca: Equus caballus, Hsa: Homo sapiens, Mdo: Monodelphis domestica, Mmu: Mus musculus, Rno: Ratus norvegicus, Sha: Sarcophilus harrisii, Ssc: Sus scrofa. (DOC) [file pone.0055402.s006.doc]

**Table S6. Normalised 454 GS FLX run data by library in counts per thousand.**

| **miRNA name** | **IB** | **WB** | **LD** | **LW** | **PT** | **ME** | **VT** |
| --- | --- | --- | --- | --- | --- | --- | --- |
| Hsa-miR-200b-3p | 59.42 | 267.74 | 313.63 | 216.80 | 182.19 | 401.39 | 101.57 |
| Ssc-miR-125b | 140.97 | 67.70 | 68.08 | 91.12 | 114.87 | 45.84 | 43.60 |
| Ssc-miR-23b | 28.84 | 53.94 | 46.56 | 39.26 | 53.92 | 38.66 | 54.53 |
| Ssc-miR-126 | 39.28 | 50.46 | 30.58 | 54.92 | 58.67 | 37.92 | 34.65 |
| Bta-miR-23a | 30.58 | 45.02 | 39.04 | 33.63 | 50.21 | 34.35 | 70.76 |
| Ssc-miR-192 | 22.13 | 39.31 | 14.16 | 46.40 | 40.07 | 27.80 | 25.80 |
| Ssc-miR-99a | 84.29 | 33.44 | 27.36 | 37.12 | 43.93 | 13.44 | 25.13 |
| Hsa-miR-200c-3p | 10.44 | 19.96 | 22.55 | 45.18 | 37.69 | 36.25 | 16.39 |
| Ssc-miR-10b | 17.16 | 32.03 | 19.78 | 21.16 | 25.67 | 32.23 | 18.76 |
| Ssc-miR-126* | 14.92 | 23.92 | 28.24 | 21.12 | 29.63 | 24.01 | 21.91 |
| Ssc-miR-30d | 20.39 | 12.13 | 10.36 | 31.79 | 21.47 | 13.67 | 5.69 |
| Ssc-miR-125a | 25.61 | 10.44 | 14.16 | 12.93 | 14.25 | 9.94 | 6.25 |
| Ssc-miR-10a | 10.69 | 15.61 | 12.84 | 9.20 | 9.30 | 16.32 | 6.76 |
| Ssc-miR-365-3p | 2.49 | 9.13 | 12.62 | 9.45 | 12.17 | 5.74 | 3.61 |
| Ssc-miR-92a | 2.98 | 5.22 | 7.66 | 9.11 | 6.18 | 8.33 | 5.46 |
| Ssc-miR-204 | 2.49 | 3.32 | 19.78 | 7.26 | 6.58 | 3.33 | 2.37 |
| Ssc-miR-378 | 16.66 | 6.91 | 3.79 | 7.52 | 7.32 | 5.92 | 3.77 |
| Ssc-miR-26a | 0.75 | 5.11 | 5.55 | 6.68 | 6.63 | 5.23 | 2.31 |
| Bta-miR-200a | 4.48 | 4.73 | 7.22 | 3.99 | 2.87 | 5.80 | 1.52 |
| Bta-miR-193b | 17.16 | 3.97 | 6.06 | 4.28 | 3.86 | 2.07 | 1.80 |
| Ssc-miR-30e-5p | 6.71 | 3.10 | 3.14 | 5.63 | 4.60 | 3.22 | 2.87 |
| Ssc-miR-100 | 4.97 | 2.99 | 5.25 | 3.86 | 4.11 | 2.24 | 2.48 |
| Ssc-miR-99b | 3.73 | 2.34 | 3.43 | 4.28 | 3.61 | 1.61 | 2.37 |
| Bta-miR-139 | 3.23 | 2.61 | 1.61 | 4.45 | 3.36 | 2.41 | 1.69 |
| Bta-miR-30a-5p | 4.23 | 2.12 | 2.99 | 3.82 | 2.92 | 2.47 | 1.35 |
| Ssc-miR-324 | 3.48 | 1.30 | 0.80 | 5.71 | 4.55 | 1.26 | 0.85 |
| Ssc-miR-362 | 0.99 | 1.20 | 0.36 | 6.05 | 3.71 | 1.49 | 1.52 |
| Ssc-miR-429 | 0.99 | 2.45 | 4.89 | 1.93 | 2.52 | 2.93 | 1.30 |
| Hsa-miR-29c-5p | 1.74 | 2.72 | 1.24 | 2.31 | 3.26 | 1.72 | 1.97 |
| Ssc-miR-374a | 0.25 | 1.74 | 2.85 | 2.10 | 2.42 | 3.62 | 1.07 |
| Hsa-miR-500a-5p | 1.99 | 0.92 | 2.48 | 2.65 | 3.26 | 2.41 | 0.90 |
| Ssc-miR-145 | 4.48 | 1.41 | 1.90 | 1.89 | 3.12 | 1.49 | 1.01 |
| Hsa-miR-324-3p | 4.48 | 1.79 | 2.19 | 1.81 | 1.63 | 1.72 | 1.35 |
| Ssc-miR-21 | 0.25 | 1.90 | 1.97 | 2.44 | 2.42 | 1.15 | 1.13 |
| Hsa-miR-25-3p | 2.73 | 1.20 | 2.63 | 2.10 | 2.03 | 1.72 | 0.45 |
| Hsa-miR-874 | 1.99 | 1.25 | 1.61 | 2.39 | 2.62 | 1.09 | 0.73 |
| Ssc-miR-218b | 0.50 | 2.45 | 0.73 | 1.47 | 1.58 | 2.18 | 1.30 |
| Ssc-miR-191 | 3.48 | 1.74 | 0.66 | 2.31 | 1.83 | 1.32 | 0.73 |
| Hsa-miR-150-5p | 0.99 | 1.41 | 2.63 | 2.02 | 1.24 | 1.61 | 0.39 |
| Ssc-miR-193a-5p | 4.97 | 1.74 | 1.31 | 0.80 | 0.79 | 2.18 | 0.28 |
| Ssc-let-7a | 0.50 | 0.82 | 1.46 | 0.84 | 0.79 | 1.84 | 1.13 |
| Ssc-miR-532-5p | 0.50 | 0.76 | 0.80 | 0.80 | 0.59 | 2.01 | 1.13 |
| Ssc-miR-22-3p | 1.24 | 1.58 | 1.17 | 0.84 | 0.74 | 0.46 | 0.73 |
| Ssc-miR-450b-5p | 0.00 | 0.98 | 0.44 | 0.63 | 1.09 | 1.09 | 0.96 |
| Ssc-miR-664-5p | 0.99 | 0.60 | 0.66 | 1.30 | 0.74 | 0.98 | 0.11 |
| Ssc-miR-374b-5p | 0.25 | 0.38 | 2.34 | 0.88 | 0.40 | 0.69 | 0.28 |
| Hsa-let-7b-5p | 0.25 | 0.60 | 0.95 | 0.55 | 0.84 | 0.75 | 0.51 |
| Ssc-miR-151-3p | 1.24 | 0.82 | 1.09 | 0.71 | 0.40 | 0.69 | 0.17 |
| Hsa-miR-29a-5p | 0.99 | 0.49 | 0.15 | 0.92 | 1.34 | 0.29 | 0.34 |
| Hsa-miR-29b-3p | 1.24 | 0.76 | 0.07 | 1.09 | 0.89 | 0.23 | 0.39 |
| Ssc-miR-532-3p | 1.74 | 0.38 | 0.51 | 0.84 | 0.79 | 0.75 | 0.28 |
| Ssc-miR-24 | 2.73 | 0.33 | 0.66 | 0.92 | 0.49 | 0.57 | 0.34 |
| Ssc-miR-423-5p | 2.98 | 1.09 | 0.29 | 0.42 | 0.45 | 0.57 | 0.51 |
| Ssc-miR-186 | 0.99 | 0.27 | 1.24 | 0.46 | 0.35 | 1.26 | 0.39 |
| Hsa-let-7d-5p | 0.99 | 0.76 | 0.22 | 0.50 | 0.40 | 0.86 | 0.68 |
| Hsa-miR-500a-3p | 0.75 | 0.87 | 0.36 | 0.92 | 0.40 | 0.23 | 0.56 |
| Ssc-let-7c | 0.00 | 0.92 | 0.29 | 0.63 | 0.74 | 0.63 | 0.28 |
| Hsa-miR-652-3p | 2.24 | 0.43 | 1.17 | 0.34 | 0.40 | 0.75 | 0.11 |
| Ssc-miR-181a | 0.50 | 0.22 | 0.88 | 1.01 | 0.35 | 0.17 | 0.39 |
| Bta-miR-451 | 0.99 | 0.38 | 0.88 | 0.34 | 0.54 | 0.23 | 0.62 |
| Ssc-miR-450c-5p | 0.99 | 0.49 | 0.51 | 0.50 | 0.59 | 0.17 | 0.51 |
| Hsa-let-7d-3p | 1.24 | 0.38 | 0.36 | 0.42 | 0.15 | 0.57 | 0.85 |
| Hsa-miR-214-5p | 0.50 | 0.27 | 0.44 | 0.67 | 0.59 | 0.52 | 0.28 |
| Ssc-miR-19b | 1.49 | 0.65 | 0.29 | 0.50 | 0.54 | 0.23 | 0.17 |
| Dre-miR-20b | 0.50 | 0.33 | 0.51 | 0.46 | 0.25 | 0.98 | 0.11 |
| Ssc-miR-16 | 0.00 | 0.22 | 0.15 | 0.84 | 0.84 | 0.29 | 0.06 |
| Hsa-miR-140-5p | 1.49 | 0.27 | 0.36 | 0.34 | 0.30 | 0.63 | 0.39 |
| Ssc-miR-363 | 0.00 | 0.49 | 0.00 | 0.55 | 0.35 | 0.75 | 0.23 |
| Hsa-miR-200a-5p | 0.00 | 0.38 | 0.51 | 0.46 | 0.35 | 0.52 | 0.23 |
| Hsa-miR-146a-5p | 0.25 | 0.71 | 0.66 | 0.29 | 0.15 | 0.23 | 0.34 |
| Ssc-miR-30c | 0.25 | 0.38 | 0.15 | 0.63 | 0.35 | 0.23 | 0.28 |
| Ssc-miR-15b | 0.25 | 0.22 | 0.44 | 0.46 | 0.25 | 0.23 | 0.51 |
| Ssc-miR-450a | 0.75 | 0.22 | 0.00 | 0.38 | 0.79 | 0.34 | 0.06 |
| Hsa-miR-20a-5p | 0.25 | 0.54 | 0.15 | 0.25 | 0.54 | 0.34 | 0.11 |
| Ssc-miR-27b | 0.00 | 0.49 | 0.00 | 0.42 | 0.40 | 0.46 | 0.17 |
| Hsa-miR-455-5p | 0.00 | 0.33 | 0.36 | 0.38 | 0.54 | 0.17 | 0.11 |
| Hsa-miR-93-5p | 0.25 | 0.27 | 0.58 | 0.21 | 0.25 | 0.29 | 0.28 |
| Ssc-miR-503 | 0.25 | 0.16 | 0.15 | 0.17 | 0.64 | 0.11 | 0.39 |
| Ssc-miR-320 | 0.50 | 0.82 | 0.15 | 0.08 | 0.15 | 0.17 | 0.23 |
| Ssc-miR-152 | 0.75 | 0.05 | 0.36 | 0.34 | 0.30 | 0.23 | 0.17 |
| Hsa-miR-221-3p | 0.75 | 0.22 | 0.44 | 0.17 | 0.05 | 0.57 | 0.06 |
| Ssc-miR-18a | 0.25 | 0.16 | 0.00 | 0.25 | 0.59 | 0.17 | 0.17 |
| Ssc-miR-181c | 0.50 | 0.33 | 0.29 | 0.21 | 0.25 | 0.00 | 0.28 |
| Hsa-miR-193b-5p | 0.99 | 0.22 | 0.22 | 0.21 | 0.10 | 0.23 | 0.28 |
| Hsa-miR-192-3p | 0.25 | 0.27 | 0.07 | 0.42 | 0.25 | 0.11 | 0.11 |
| Ssc-miR-199a* | 0.25 | 0.27 | 0.00 | 0.38 | 0.10 | 0.11 | 0.34 |
| Ssc-miR-28-3p | 0.00 | 0.16 | 0.44 | 0.34 | 0.00 | 0.46 | 0.00 |
| Ssc-miR-29c | 0.00 | 0.16 | 0.29 | 0.34 | 0.30 | 0.00 | 0.11 |
| Ssc-miR-486 | 0.00 | 0.11 | 0.22 | 0.21 | 0.49 | 0.00 | 0.17 |
| Hsa-miR-542-5p | 0.00 | 0.11 | 0.58 | 0.17 | 0.10 | 0.06 | 0.34 |
| Hsa-let-7i-5p | 0.00 | 0.00 | 0.22 | 0.13 | 0.25 | 0.63 | 0.00 |
| Rno-miR-125b* | 0.50 | 0.11 | 0.36 | 0.13 | 0.15 | 0.40 | 0.00 |
| Ssc-miR-424 | 0.00 | 0.00 | 0.22 | 0.38 | 0.10 | 0.40 | 0.06 |
| Ssc-miR-199a-3p | 0.25 | 0.11 | 0.66 | 0.13 | 0.15 | 0.00 | 0.17 |
| Ssc-miR-29a | 0.00 | 0.11 | 0.15 | 0.34 | 0.30 | 0.00 | 0.17 |
| Bta-miR-1468 | 0.25 | 0.00 | 0.15 | 0.17 | 0.40 | 0.23 | 0.06 |
| Hsa-miR-505-3p | 0.75 | 0.11 | 0.15 | 0.17 | 0.20 | 0.17 | 0.11 |
| Ssc-miR-32 | 0.00 | 0.16 | 0.29 | 0.29 | 0.15 | 0.06 | 0.06 |
| Ssc-miR-676-3p | 0.25 | 0.05 | 0.22 | 0.04 | 0.10 | 0.11 | 0.45 |
| Ssc-let-7f | 0.00 | 0.05 | 0.00 | 0.13 | 0.10 | 0.57 | 0.06 |
| Ssc-miR-30b-5p | 0.00 | 0.27 | 0.00 | 0.08 | 0.25 | 0.17 | 0.11 |
| Ssc-miR-425-3p | 0.00 | 0.49 | 0.00 | 0.04 | 0.30 | 0.00 | 0.00 |
| Hsa-miR-4454 | 0.00 | 0.16 | 0.58 | 0.13 | 0.05 | 0.06 | 0.00 |
| Rno-miR-551b | 0.75 | 0.11 | 0.15 | 0.08 | 0.00 | 0.11 | 0.28 |
| Ssc-miR-769-5p | 0.50 | 0.27 | 0.07 | 0.08 | 0.05 | 0.11 | 0.06 |
| Ssc-miR-140* | 0.00 | 0.05 | 0.07 | 0.17 | 0.15 | 0.23 | 0.00 |
| Ssc-miR-151-5p | 0.25 | 0.11 | 0.51 | 0.04 | 0.00 | 0.06 | 0.06 |
| Ssc-let-7e | 0.00 | 0.11 | 0.22 | 0.13 | 0.10 | 0.11 | 0.00 |
| Ssc-miR-128 | 0.25 | 0.27 | 0.15 | 0.00 | 0.10 | 0.11 | 0.00 |
| Hsa-miR-194-5p | 0.50 | 0.11 | 0.07 | 0.21 | 0.05 | 0.06 | 0.00 |
| Ssc-miR-328 | 0.00 | 0.05 | 0.29 | 0.17 | 0.05 | 0.06 | 0.06 |
| Ssc-miR-361-5p | 0.00 | 0.11 | 0.07 | 0.21 | 0.05 | 0.00 | 0.11 |
| Ssc-miR-143-3p | 0.00 | 0.00 | 0.00 | 0.04 | 0.25 | 0.23 | 0.00 |
| Ssc-miR-199a | 0.25 | 0.00 | 0.07 | 0.04 | 0.10 | 0.29 | 0.00 |
| Hsa-miR-505-5p | 0.25 | 0.00 | 0.07 | 0.21 | 0.05 | 0.11 | 0.00 |
| Hsa-miR-106b-3p | 0.75 | 0.00 | 0.15 | 0.08 | 0.05 | 0.00 | 0.06 |
| Ssc-miR-130a | 0.00 | 0.05 | 0.00 | 0.17 | 0.15 | 0.00 | 0.06 |
| Ssc-miR-196b-5p | 0.00 | 0.22 | 0.07 | 0.00 | 0.05 | 0.06 | 0.11 |
| Ssc-miR-34a | 0.00 | 0.00 | 0.15 | 0.04 | 0.10 | 0.17 | 0.06 |
| Ssc-miR-129a | 0.00 | 0.11 | 0.07 | 0.13 | 0.00 | 0.06 | 0.06 |
| Ssc-miR-27a | 0.00 | 0.00 | 0.22 | 0.17 | 0.05 | 0.00 | 0.00 |
| Ssc-miR-30e-3p | 0.50 | 0.05 | 0.00 | 0.13 | 0.00 | 0.11 | 0.00 |
| Ssc-miR-331-5p | 0.00 | 0.00 | 0.00 | 0.04 | 0.00 | 0.29 | 0.11 |
| Hsa-miR-335-3p | 0.00 | 0.05 | 0.07 | 0.00 | 0.10 | 0.17 | 0.06 |
| Hsa-miR-378a-5p | 0.25 | 0.00 | 0.15 | 0.04 | 0.00 | 0.17 | 0.06 |
| Bta-miR-423-3p | 0.25 | 0.00 | 0.00 | 0.00 | 0.10 | 0.00 | 0.28 |
| Hsa-miR-4286 | 0.00 | 0.11 | 0.15 | 0.04 | 0.15 | 0.00 | 0.00 |
| Hsa-miR-551a | 0.25 | 0.05 | 0.00 | 0.04 | 0.25 | 0.00 | 0.00 |
| Mdo-miR-106 | 0.00 | 0.05 | 0.00 | 0.08 | 0.20 | 0.00 | 0.00 |
| Ssc-miR-195 | 0.00 | 0.00 | 0.15 | 0.08 | 0.05 | 0.11 | 0.00 |
| Ssc-miR-199b* | 0.00 | 0.05 | 0.00 | 0.04 | 0.05 | 0.23 | 0.00 |
| Ssc-miR-339-5p | 0.00 | 0.00 | 0.29 | 0.00 | 0.00 | 0.17 | 0.00 |
| Hsa-miR-9-3p | 0.00 | 0.00 | 0.00 | 0.08 | 0.20 | 0.06 | 0.00 |
| Hsa-let-7b-3p | 0.25 | 0.11 | 0.00 | 0.00 | 0.00 | 0.17 | 0.00 |
| Hsa-miR-125a-3p | 0.00 | 0.00 | 0.07 | 0.04 | 0.10 | 0.11 | 0.00 |
| Ssc-miR-17-5p | 0.00 | 0.00 | 0.07 | 0.04 | 0.10 | 0.11 | 0.00 |
| Ssc-miR-20 | 0.00 | 0.00 | 0.00 | 0.08 | 0.05 | 0.17 | 0.00 |
| Bta-miR-2483 | 0.00 | 0.05 | 0.00 | 0.00 | 0.20 | 0.06 | 0.00 |
| Ssc-miR-92b-3p | 0.00 | 0.05 | 0.07 | 0.13 | 0.00 | 0.06 | 0.00 |
| Ssc-miR-130b | 0.00 | 0.05 | 0.00 | 0.08 | 0.00 | 0.06 | 0.06 |
| Ssc-miR-181b | 0.00 | 0.11 | 0.00 | 0.04 | 0.05 | 0.00 | 0.06 |
| Mmu-miR-29b-2-5p | 0.25 | 0.05 | 0.07 | 0.04 | 0.00 | 0.00 | 0.06 |
| Ssc-miR-4334-3p | 0.00 | 0.00 | 0.22 | 0.04 | 0.05 | 0.00 | 0.00 |
| Eca-miR-545 | 0.00 | 0.00 | 0.00 | 0.08 | 0.15 | 0.00 | 0.00 |
| Sha-miR-716b | 0.00 | 0.22 | 0.07 | 0.00 | 0.00 | 0.00 | 0.00 |
| Hsa-miR-138-5p | 0.00 | 0.00 | 0.00 | 0.00 | 0.10 | 0.00 | 0.11 |
| Ssc-miR-183 | 0.00 | 0.05 | 0.15 | 0.04 | 0.00 | 0.00 | 0.00 |
| Hsa-miR-18a-3p | 0.00 | 0.00 | 0.00 | 0.04 | 0.05 | 0.00 | 0.11 |
| Ssc-miR-193a-3p | 0.50 | 0.11 | 0.00 | 0.00 | 0.00 | 0.00 | 0.00 |
| Hsa-miR-31-3p | 0.00 | 0.22 | 0.00 | 0.00 | 0.00 | 0.00 | 0.00 |
| Ssc-miR-340 | 0.00 | 0.05 | 0.00 | 0.00 | 0.15 | 0.00 | 0.00 |
| Ssc-miR-345-3p | 0.50 | 0.00 | 0.15 | 0.00 | 0.00 | 0.00 | 0.00 |
| Ssc-miR-424* | 0.00 | 0.22 | 0.00 | 0.00 | 0.00 | 0.00 | 0.00 |
| Ssc-miR-542-3p | 0.25 | 0.00 | 0.15 | 0.00 | 0.00 | 0.00 | 0.06 |
| Ssc-miR-574 | 0.00 | 0.00 | 0.07 | 0.04 | 0.10 | 0.00 | 0.00 |
| Ssc-miR-9-1 | 0.00 | 0.16 | 0.00 | 0.04 | 0.00 | 0.00 | 0.00 |
| Hsa-miR-99a-3p | 0.00 | 0.00 | 0.00 | 0.13 | 0.05 | 0.00 | 0.00 |
| Hsa-let-7e-3p | 0.00 | 0.00 | 0.00 | 0.00 | 0.00 | 0.17 | 0.00 |
| Ssc-let-7g | 0.00 | 0.00 | 0.00 | 0.04 | 0.05 | 0.06 | 0.00 |
| Hsa-miR-1271-3p | 0.00 | 0.00 | 0.07 | 0.00 | 0.10 | 0.00 | 0.00 |
| Ssc-miR-1343 | 0.00 | 0.00 | 0.00 | 0.00 | 0.00 | 0.11 | 0.06 |
| Hsa-miR-200b-5p | 0.00 | 0.16 | 0.00 | 0.00 | 0.00 | 0.00 | 0.00 |
| Mmu-miR-2137 | 0.00 | 0.05 | 0.00 | 0.08 | 0.00 | 0.00 | 0.00 |
| Hsa-miR-28-5p | 0.00 | 0.00 | 0.22 | 0.00 | 0.00 | 0.00 | 0.00 |
| Ssc-miR-345-5p | 0.00 | 0.00 | 0.07 | 0.00 | 0.05 | 0.00 | 0.06 |
| Ssc-miR-361-3p | 0.00 | 0.05 | 0.07 | 0.00 | 0.00 | 0.06 | 0.00 |
| Mmu-miR-5100 | 0.00 | 0.11 | 0.00 | 0.04 | 0.00 | 0.00 | 0.00 |

IB: Iberian breed, WB: European Wild Boar, LD: Landrace breed, LW: Large White breed, PT: Piétrain breed, ME: MeiShan breed, VT: Vietnamese breed.
miRNA name represents the most expressed sequence in the cluster.
Bta: *Bos taurus*, Dre: *Danio rerio*, Eca: *Equus caballus*, Hsa: *Homo sapiens*, Mdo: *Monodelphis domestica*, Mmu: *Mus musculus*, Rno: *Ratus norvegicus*, Sha: *Sarcophilus harrisii*, Ssc: *Sus scrofa*.
