# Supplementary material for: miRNA Expression Profile Analysis in Kidney of Different Porcine Breeds
Source: PLoS One. 2013 Jan 25;8(1):e55402. doi: 10.1371/journal.pone.0055402 (PMC3555835; doi:10.1371/journal.pone.0055402)
Supplement: Table S7 — Resulting clusters from sequence analysis for novel miRNAs discovery. 1: chromosome: start position: end position: strand. Sequences mapped at pig genome sequence (Sscrofa 9.62). (DOC) [file pone.0055402.s007.doc]

**Table S7. Resulting clusters from** sequence analysis for novel miRNAs discovery.

| **Cluster** | **Total Counts** | **IsomiRs** | **Sequence (5’-3’)** | **Nt** | **Chromosome position^1^** | **Compatible pre-miRNA folding structure** |
| --- | --- | --- | --- | --- | --- | --- |
| Cl-2 | 4 | 1 | CTGCTATGCCAACATATTGCCA | 22 | 1:209939258:209939279:+ | Yes |
| Cl-4 | 3 | 1 | ACTGCCCCAGGTGCTGCTGGAG | 22 | 1:289348095:289348116:- | No |
| Cl-5 | 12 | 2 | CTGTAACAGCAACTCCATGTGGAA | 24 | 2:5685220:5685243:+ | Yes |
| Cl-7 | 4 | 1 | GAGTGTCAGGAGGCAGGAGGAGC | 23 | 2:55972963:55972985:-+the number of sequence > 2, 19000000000000000000000000000000000000000000000000000000000000000000000000000000000000000000000000 | No |
| Cl-10 | 6 | 1 | TTTCCTGGGATTCCAGTGGGC | 21 | 5:7896417:7896437:+ | No |
| Cl-15 | 9 | 2 | GCGACCCACTCTTGGTTTCCATG | 23 | 6:43640521:43640543:- | Yes |
| Cl-16 | 6 | 1 | TTGGTGACCAGGTGCTCAGGGAG | 23 | 6:60299775:60299797:--000000000000000000000000000000000000000000000000000000000000000000000000000000000000000000000000000000000000000000000000000000 | Yes |
| Cl-20 | 6 | 1 | GAGTACCCTGAGGAAAAGAA | 20 | 7:16488827:16488846:+ | No |
| Cl-21 | 3 | 1 | TCACTCGAAAATACACGCTGCCC | 23 | 9:24072264:24072286:+ | No |
| Cl-22 | 6 | 1 | TTCTGTGATGGTTTCTGAGATGA | 23 | 9:26761843:26761865:+ | No |
| Cl-23 | 3 | 2 | AATCCCGGACGAGCCCCCAAAT | 22 | 9:96465803:96465824:- | No |
| Cl-24 | 3 | 2 | CTGCATTTCCTGGCTGCCTTAATT | 24 | 11:68226148: 68226171:+ | Yes |
| Cl-25 | 7 | 3 | CAGCTGGTGTTGTGAATCAGGCCG | 24 | 13:23336889:23336912:+ | Yes |
| Cl-29 | 3 | 1 | GTTGGTGTACACTGGAATAGCT | 22 | 16:15411586:15411607:+ | Yes |
| Cl-38 | 21 | 3 | TCTCCGTTTGCCTGTTTTGCTGA | 23 | X:50243082:50243104:+ | Yes |

^1^: chromosome : start position : end position : strand. Sequences mapped at pig genome sequence (Sscrofa 9.62).
