# Supplementary material for: miRNA Expression Profile Analysis in Kidney of Different Porcine Breeds
Source: PLoS One. 2013 Jan 25;8(1):e55402. doi: 10.1371/journal.pone.0055402 (PMC3555835; doi:10.1371/journal.pone.0055402)
Supplement: Table S8 — Putative target genes of the eight differentially expressed miRNAs analised by qRT-PCR. Bta: Bos taurus, Hsa: Homo sapiens, Ssc: Sus scrofa. Potential mRNA target genes for differentially expressed miRNAs predicted in silico with DIANA – microT v3.0 web server. (DOC) [file pone.0055402.s008.doc]

**Table S8. Putative target genes of the eight differentially expressed miRNAs analised by qRT-PCR.**

| **miRNA** | **Target genes** |
| --- | --- |
| Hsa-miR-200b-3p | ABAT; ABCC9; ABI2; ACTC1; ACVR2A; ACVR2B; ACY1L2; ADAM12; ADAMTS3; ADCY9; ADCYAP1; ADD3; ADIPOR2; AFF1; AFF3; AFF4; AKAP2; PALM2; AKT3; AMFR; AMMECR1L; AMOTL2; ANGEL2; ANK3; ANKRD25; ANKRD28; ANLN; AP1S2; APAF1; APLP2; APOO; ARHGAP19; ARHGAP20; ARHGAP6; ARID2; ARID4A; ARID4B; ARID5B; ARIH2; ARL2BP; ARL5A; ARL6IP2; ASF1A; ASH1L; ASXL1; ATF7; ATP11B; ATP11C; ATPAF1; ATRX; ATXN1; B3GALNT1; B3GALTL; B3GNT1; B3GNT2; B4GALT6; BACH2; BAP1; BASP1; BAT2D1; BCL11B; BCL2; BCL2L11; BDKRB2; BDP1; BICC1; BICD2; BMPER; BNC2; BPTF; BTF3L4; C10orf118; C10orf56; C10orf6; C14orf129; C14orf83; C16orf72; C19orf7; C1orf71; C1orf96; C21orf91; C2orf37; C3orf23; C5orf24; C6orf120; C6orf167; C8orf79; C9orf25; CACHD1; CACNB2; CALU; CAMSAP1L1; CASC4; CASD1; CASK; CASR; CASZ1; CBL; CBX4; CCDC100; CCDC144A; CCDC144B; CCDC82; CCNE2; CCNJ; CCNL2; CCNYL1; CD59; CDC14A; CDC14B; CDC14C; CDC42; CDC42BPA; CDC73; CDH11; CDH6; CDH7; CDK2; CDYL; CEP350; CFL2; CHD1; CHD9; CHM; CHMP5; CHN2; CHRDL1; CHSY1; CITED2; CLASP2; CLCC1; CLIC4; CLIP1; CLIP2; CLOCK; CNKSR3; CNOT6; CNOT6L; CNTNAP2; COL4A3; COL4A3BP; COMMD3; COPS8; CORO1C; COX11; CPNE2; CREB5; CRKL; CRTAP; CSMD3; CSNK1G3; CTBP2; CTDSPL2; CTNND2; CUGBP2; CYP1B1; DACH1; DAZ1; DAZ4; DAZ2; DAZ3; DCP2; DCX; DDEF1; DDX1; DDX26B; DDX3X; DDX3Y; DLC1; DLGAP2; DMD; DMRT2; DNA2L; DNAJB5; DNAJB9; DNAJC5; DOCK4; DPY19L1; DR1; DST; DTNA; DUSP1; DZIP1; E2F3; EDEM3; EDG2; EDNRA; EFNA1; EFNB2; EGLN1; EIF2S1; EIF4G1; EIF5B; ELAVL2; ELAVL4; ELF2; ELL; ELL2; ELMOD2; ENAH; ENDOD1; EP300; EPS15; EPS8; ERBB4; ERG; ERGIC2; ERRFI1; ESRRG; ETS1; ETS2; ETV5; EVI5; EXOC5; EXT1; FAM105B; FAM107B; FAM123A; FAM135B; FAM19A5; FAM26C; FAM60A; FAM76B; FAM8A1; FARP1; FAT3; FBXO33; FBXW11; FBXW7; FHL1; FHOD1; FIGN; FLI1; FLT1; FMR1; FN1; FNDC3B; FOXF1; FOXG1; FOXN2; FOXP1; FRAS1; FREM2; FRMD4A; FRMD4B; FRMD6; FRS2; FSD1L; FSTL1; FUBP3; FUT4; FXR1; G6PC; GABPA; GATA2; GDAP1; GDI2; GIT2; GJA7; GLCCI1; GLI3; GMFB; GNAI3; GOLGA1; GOLGA7; GOLGA8A; GOLGA8B; GOLGA8E; GOLGA8F; GOLGA8G; GOSR2; GPATCH8; GPM6A; GPR107; GPR158; GRB10; GRID2; GRIN2A; GTF2E1; GUCY1A3; HBS1L; HCCS; HCN1; HECTD2; HIPK1; HLF; HMBOX1; HMGB3; HNF1B; HNRNPU; HNRPD; HNRPK; HOOK1; HOXA5; HPS5; HRB; HS2ST1; HS3ST1; HSPA9; ICA1L; ICK; IFIT5; IHPK1; IKBKB; IKZF2; IMMP2L; ING2; INSM2; INTS8; IPO8; IRX5; ITM2B; ITPR1; IYD; JAG2; JAZF1; JHDM1D; JMJD2A; JUN; KATNAL1; KBTBD6; KCND2; KCND3; KCNJ2; KCNK2; KCNQ4; KCTD8; KDELC1; KDR; KHDRBS2; KIAA0101; KIAA0152; KIAA0182; KIAA0355; KIAA0423; KIAA0430; KIAA1012; KIAA1128; KIAA1244; KIAA1432; KIAA1462; KIAA1468; KIAA1546; KIAA1949; KIAA2018; KIF13A; KLF12; KLF9; KLHDC5; KLHL14; KLHL24; KLHL3; LAMC1; LASS6; LBR; LCORL; LEMD3; LEPR; LHFP; LIN28B; LMO7; LPHN2; LPIN1; LRIG1; LRP1; LRP1B; LRRC8A; LRRIQ2; LRRTM3; LYPLA2; LYPLA2P1; LYRM2; MAF; MAFG; MAMDC2; MAP1B; MAP2; MAP3K1; MAP3K7IP3; MAP4K3; MAP4K4; MAP4K5; MAPRE1; MARCKS; MATR3; MBNL2; MBOAT2; MCFD2; MED13; MEF2D; MEGF11; MEX3B; MEX3C; MFHAS1; MGA; MGAT2; MGAT4A; MIB1; MIER3; MIPOL1; MKL2; MKLN1; MKRN1; MLL; MLL5; MMAA; MMD; MMD2; MMP16; MOBKL1A; MON2; MRPL19; MSL2L1; MSN; MTAP; MTFR1; MTMR14; MTMR9; MXD1; MYB; MYCN; MYLK; MYT1; NAB1; NAP1L5; NARG1L; NCAM1; NCOA3; NCOA4; NCOR2; NDN; NDUFS4; NEDD1; NEDD4; NEGR1; NET1; NF1; NFASC; NFIB; NFYA; NGEF; NIN; NIPBL; NLGN4X; NOG; NOTCH1; NOVA1; NPC1; NPTX1; NPY1R; NR2C2; NR2F2; NR3C1; NR4A2; NR5A2; NRBF2; NRBP1; NRIP1; NTF3; NTRK3; NUDT4; NUMB; NUP153; OBFC2B; ODF2L; ODZ1; ONECUT2; OSBPL11; OSBPL8; OSTM1; OTUD4; OXR1; PAG1; PAIP2; PAK7; PAN3; PAPD5; PAPOLG; PARD6B; PAX6; PBX3; PCAF; PCDH10; PCDH19; PCDH7; PCDH8; PCMTD1; PCSK2; PCTK2; PDCD10; PDE5A; PDIK1L; PDS5B; PELI2; PERQ1; PGM2L1; PHACTR3; PHF10; PHF17; PHF21A; PHF21B; PHF6; PHTF2; PI4KB; PIGM; PIP5K3; PKD1; PKIA; PKN2; PKP4; PLCG1; PLCXD1; PLCXD3; PLEKHC1; PLEKHK1; PLK2; PLXNA2; PMAIP1; PMPCB; POLK; POU2F1; PPAPDC2; PPARGC1A; PPFIA1; PPM1E; PPM1F; PPP1CB; PPP1R12B; PPP1R2; PPP1R9A; PPP2R2C; PPP2R5C; PPP2R5E; PPP4R2; PRDM1; PRDM16; PRKACB; PRKCA; PRKG1; PROX1; PSCD1; PSCD3; PSIP1; PSPH; PSPHL; PTGER3; PTP4A1; PTPN11; PTPN12; PTPRZ1; PUM2; PVRL1; PVRL4; QKI; RAB11FIP2; RAB18; RAB33B; RAB37; RAB7A; RAB8B; RANBP10; RANBP9; RAP1B; RAP2C; RAPGEF2; RBM26; RBM35A; RDH10; RECK; REEP1; RELN; RET; REV1; RFXDC2; RGL1; RHOA; RHOT1; RIPK2; RLF; RND3; RNF19A; RNF2; RNF216L; ROCK2; ROD1; RPS6KA3; RPS6KB1; RRP15; RSBN1; RTF1; RUSC2; S100PBP; SAPS3; SASH1; SATB2; SCAMP1; SCN2A; SCN3A; SCN3B; SCN5A; SCRT2; SEC23A; SEMA6D; SENP6; SERPINI1; SESN1; SFRS1; SH3PXD2A; SHC4; SHROOM1; SHROOM4; SIAH1; SIRT1; SLAIN1; SLC14A1; SLC16A2; SLC1A1; SLC1A2; SLC23A2; SLC24A4; SLC25A16; SLC31A1; SLC35B4; SLC35F5; SLC38A2; SLC4A4; SLC4A7; SLC5A3; SLC6A1; SLC6A17; SLC7A11; SLITRK1; SLK; SMAD5; SMARCAD1; SMCR7L; SMURF2; SNAP25; SNAPC1; SNTB2; SNX16; SNX30; SOCS4; SOCS6; SORT1; SOS1; SOX1; SOX2; SPRED1; SPRYD4; SRF; SRP72; STAM2; STCH; STK38L; STRN3; STX16; STX1A; STXBP6; STYX; SULF1; SUPV3L1; SUSD5; SUZ12; SYDE1; SYNJ1; SYS1; SYT1; SYVN1; TAF12; TAF9B; TARDBP; TBC1D12; TBX5; TCEB1; TCP11L1; TEAD1; TFAP2A; TFEC; THAP1; THAP2; THEX1; THRA; THRB; THSD7A; TIMP2; TLL2; TLN2; TMCC1; TMEFF2; TMEM164; TMEM16E; TMEM16F; TMEM178; TMEM26; TMEM46; TMTC1; TNRC6B; TOPORS; TP53INP1; TRIM33; TRIM62; TRPS1; TSC22D1; TSC22D2; TSGA14; TSHZ2; TXNDC13; UBE1L2; UBE2B; UBE2I; UBE2R2; UBE2W; UBQLN1; UBTD2; ULK2; USH1G; USH2A; USP47; USP6NL; VASH2; VCPIP1; VEGFA; VEZF1; VKORC1L1; VTI1A; WAPAL; WASF1; WASF3; WDFY3; WDR68; WDR82; WDR91; WHSC1; WIPF1; WNT16; XKR4; XKR8; YME1L1; YOD1; YPEL2; YTHDF3; YWHAB; YWHAG; YWHAQ; ZBTB5; ZBTB8; ZC3H12B; ZC3H6; ZCCHC14; ZDHHC17; ZEB1; ZEB2; ZFAND6; ZFPM2; ZFR; ZFX; ZFYVE20; ZMYM4; ZMYND8; ZNF207; ZNF217; ZNF292; ZNF294; ZNF395; ZNF423; ZNF532; ZNF652; ZNF655; ZNF662; ZNF677; ZNF711. |
| Hsa-miR-200c-3p | ABAT; ABCC9; ABI2; ACTC1; ACVR2A; ACVR2B; ACY1L2; ADAM12; ADAMTS3; ADCY9; ADCYAP1; ADD3; ADIPOR2; AFF1; AFF3; AFF4; AKAP2; PALM2; AKT3; AMFR; AMMECR1L; AMOTL2; ANGEL2; ANK3; ANKRD25; ANKRD28; ANLN; AP1S2; APAF1; APLP2; APOO; ARHGAP19; ARHGAP20; ARHGAP6; ARID2; ARID4A; ARID4B; ARID5B; ARIH2; ARL2BP; ARL5A; ARL6IP2; ASF1A; ASH1L; ASXL1; ATF7; ATP11B; ATP11C; ATPAF1; ATRX; ATXN1; B3GALNT1; B3GALTL; B3GNT1; B3GNT2; B4GALT6; BACH2; BAP1; BASP1; BAT2D1; BCL11B; BCL2; BCL2L11; BDKRB2; BDP1; BICC1; BICD2; BMPER; BNC2; BPTF; BTF3L4; C10orf118; C10orf56; C10orf6; C14orf129; C14orf83; C16orf72; C19orf7; C1orf71; C1orf96; C21orf91; C2orf37; C3orf23; C5orf24; C6orf120; C6orf167; C8orf79; C9orf25; CACHD1; CACNB2; CALU; CAMSAP1L1; CASC4; CASD1; CASK; CASR; CASZ1; CBL; CBX4; CCDC144A; CCDC144B; CCDC82; CCNE2; CCNJ; CCNL2; CCNYL1; CD59; CDC14A; CDC14B; CDC14C; CDC42; CDC42BPA; CDC73; CDH11; CDH6; CDH7; CDK2; CDYL; CEP350; CFL2; CGGBP1; CHD1; CHD9; CHM; CHMP5; CHN2; CHRDL1; CHSY1; CITED2; CLASP2; CLCC1; CLIC4; CLIP1; CLIP2; CLOCK; CNKSR3; CNOT6; CNOT6L; CNTNAP2; COL4A3; COL4A3BP; COMMD3; COPS8; CORO1C; COX11; CPNE2; CREB5; CRKL; CRTAP; CSMD3; CSNK1G3; CTBP2; CTDSPL2; CTNND2; CUGBP2; CYP1B1; DACH1; DAZ1; DAZ4; DAZ2; DAZ3; DAZL; DCP2; DCX; DDEF1; DDX1; DDX26B; DDX3X; DDX3Y; DLC1; DLGAP2; DMD; DMRT2; DNA2L; DNAJB5; DNAJB9; DNAJC5; DOCK4; DPY19L1; DR1; DST; DTNA; DUSP1; DZIP1; E2F3; EDEM3; EDG2; EDNRA; EFNA1; EFNB2; EGLN1; EIF2S1; EIF4G1; EIF5B; ELAVL2; ELAVL4; ELF2; ELL; ELL2; ELMOD2; ENAH; ENDOD1; EP300; EPS15; EPS8; ERBB4; ERG; ERGIC2; ERRFI1; ESRRG; ETS1; ETS2; ETV5; EVI5; EXOC5; EXT1; FAM105B; FAM107B; FAM123A; FAM135B; FAM19A5; FAM26C; FAM60A; FAM76B; FAM8A1; FARP1; FAT3; FBXO33; FBXW11; FBXW7; FHL1; FHOD1; FIGN; FLI1; FLT1; FMR1; FN1; FNDC3B; FOXF1; FOXG1; FOXN2; FOXP1; FRAS1; FREM2; FRMD4A; FRMD4B; FRMD6; FRS2; FSD1L; FSTL1; FUBP3; FUT4; FXR1; G6PC; GABPA; GATA2; GDAP1; GDI2; GIT2; GJA7; GLCCI1; GLI3; GMFB; GNAI3; GOLGA1; GOLGA7; GOLGA8A; GOLGA8B; GOLGA8E; GOLGA8F; GOLGA8G; GOSR2; GPATCH8; GPM6A; GPR107; GPR158; GRB10; GRID2; GRIN2A; GTF2E1; GUCY1A3; HBS1L; HCCS; HCN1; HECTD2; HIPK1; HLF; HMBOX1; HMGB3; HNF1B; HNRNPU; HNRPD; HNRPK; HOOK1; HOXA5; HPS5; HRB; HS2ST1; HS3ST1; HSPA9; ICA1L; ICK; IFIT5; IHPK1; IKBKB; IKZF2; IMMP2L; ING2; INSM2; INTS8; IPO8; IRX5; ITM2B; ITPR1; IYD; JAG2; JAZF1; JHDM1D; JMJD2A; JUN; KATNAL1; KBTBD6; KCND2; KCND3; KCNJ2; KCNK2; KCNQ4; KCTD8; KDELC1; KDR; KHDRBS2; KIAA0101; KIAA0152; KIAA0182; KIAA0355; KIAA0423; KIAA0430; KIAA1012; KIAA1128; KIAA1244; KIAA1432; KIAA1462; KIAA1468; KIAA1546; KIAA1949; KIAA2018; KIF13A; KLF12; KLF9; KLHDC5; KLHL14; KLHL24; KLHL3; LAMC1; LASS6; LBR; LCORL; LEMD3; LEPR; LHFP; LIN28B; LMO7; LPHN2; LPIN1; LRIG1; LRP1; LRP1B; LRRC8A; LRRIQ2; LRRTM3; LYPLA2; LYPLA2P1; LYRM2; MAF; MAFG; MAMDC2; MAP1B; MAP2; MAP3K1; MAP3K7IP3; MAP4K3; MAP4K4; MAP4K5; MAPRE1; MARCKS; MATR3; MBNL2; MBOAT2; MCFD2; MED13; MEF2D; MEGF11; MEX3B; MEX3C; MFHAS1; MGA; MGAT2; MGAT4A; MIB1; MIER3; MIPOL1; MKL2; MKLN1; MKRN1; MLL; MLL5; MMAA; MMD; MMD2; MMP16; MOBKL1A; MON2; MRPL19; MSL2L1; MSN; MTAP; MTFR1; MTMR14; MTMR9; MXD1; MYB; MYCN; MYLK; MYT1; NAB1; NAP1L5; NARG1L; NCAM1; NCOA3; NCOA4; NCOR2; NDN; NDUFS4; NEDD1; NEDD4; NEGR1; NET1; NF1; NFASC; NFIB; NFYA; NGEF; NIN; NIPBL; NLGN4X; NOG; NOTCH1; NOVA1; NPC1; NPTX1; NPY1R; NR2C2; NR2F2; NR3C1; NR4A2; NR5A2; NRBF2; NRBP1; NRIP1; NTF3; NTRK3; NUDT4; NUMB; NUP153; OBFC2B; ODF2L; ODZ1; ONECUT2; OSBPL11; OSBPL8; OSTM1; OTUD4; OXR1; PAG1; PAIP2; PAK7; PAN3; PAPD5; PAPOLG; PARD6B; PBX3; PCAF; PCDH10; PCDH19; PCDH7; PCDH8; PCMTD1; PCSK2; PCTK2; PDCD10; PDE5A; PDIK1L; PDS5B; PELI2; PERQ1; PGM2L1; PHACTR3; PHF10; PHF17; PHF21A; PHF21B; PHF6; PHTF2; PI4KB; PIGM; PIP5K3; PKD1; PKIA; PKN2; PKP4; PLCG1; PLCXD1; PLCXD3; PLEKHC1; PLEKHK1; PLK2; PLXNA2; PMAIP1; PMPCB; POLK; POU2F1; PPAP2B; PPAPDC2; PPARGC1A; PPFIA1; PPM1E; PPM1F; PPP1CB; PPP1R12B; PPP1R2; PPP1R9A; PPP2R2C; PPP2R5C; PPP2R5E; PPP4R2; PRDM1; PRDM16; PRKACB; PRKCA; PRKG1; PROX1; PSCD1; PSCD3; PSIP1; PSPH; PSPHL; PTGER3; PTP4A1; PTPN11; PTPN12; PTPRZ1; PUM2; PVRL1; PVRL4; QKI; RAB11FIP2; RAB18; RAB33B; RAB37; RAB7A; RAB8B; RANBP10; RANBP9; RAP1B; RAP2C; RAPGEF2; RBM26; RBM35A; RDH10; RECK; REEP1; RELN; RET; REV1; RFXDC2; RGL1; RHOA; RHOT1; RIPK2; RLF; RND3; RNF19A; RNF2; RNF216L; RNF38; ROCK2; ROD1; RPS6KA3; RPS6KB1; RRP15; RSBN1; RTF1; RUSC2; S100PBP; SAPS3; SASH1; SATB2; SCAMP1; SCN2A; SCN3A; SCN3B; SCN5A; SCRT2; SEC23A; SEMA6D; SENP6; SERPINI1; SESN1; SFRS1; SGMS1; SH3PXD2A; SHC4; SHROOM1; SHROOM4; SIAH1; SIRT1; SLAIN1; SLC14A1; SLC16A2; SLC1A1; SLC1A2; SLC23A2; SLC24A4; SLC25A16; SLC31A1; SLC35B4; SLC35F5; SLC38A2; SLC4A4; SLC4A7; SLC5A3; SLC6A1; SLC6A17; SLC7A11; SLITRK1; SLK; SMAD5; SMARCAD1; SMCR7L; SMURF2; SNAP25; SNAPC1; SNTB2; SNX16; SNX30; SOCS4; SOCS6; SORT1; SOS1; SOX1; SOX2; SPRED1; SPRYD4; SRF; SRP72; STAM2; STCH; STK38L; STRN3; STX16; STX1A; STXBP6; STYX; SULF1; SUPV3L1; SUSD5; SUZ12; SYDE1; SYNJ1; SYS1; SYT1; SYVN1; TAF12; TAF9B; TARDBP; TBC1D12; TBX5; TCEB1; TCP11L1; TEAD1; TFAP2A; TFEC; THAP1; THAP2; THEX1; THRA; THRB; THSD7A; TIMP2; TLL2; TLN2; TMCC1; TMEFF2; TMEM164; TMEM16E; TMEM16F; TMEM178; TMEM26; TMEM46; TMTC1; TNRC6B; TOPORS; TP53INP1; TRIM33; TRIM62; TRPS1; TSC22D1; TSC22D2; TSGA14; TXNDC13; UBE1L2; UBE2B; UBE2I; UBE2R2; UBE2W; UBQLN1; UBTD2; ULK2; USH1G; USH2A; USP47; USP6NL; VASH2; VCPIP1; VEGFA; VEZF1; VKORC1L1; VTI1A; WAPAL; WASF1; WASF3; WDFY3; WDR68; WDR82; WDR91; WHSC1; WIPF1; WNT16; XKR4; XKR8; YME1L1; YOD1; YPEL2; YTHDF3; YWHAB; YWHAG; YWHAQ; ZBTB5; ZBTB8; ZC3H12B; ZC3H6; ZCCHC14; ZDHHC17; ZEB1; ZEB2; ZFAND6; ZFPM2; ZFR; ZFX; ZFYVE20; ZMYM4; ZMYND8; ZNF207; ZNF217; ZNF292; ZNF294; ZNF395; ZNF423; ZNF532; ZNF652; ZNF655; ZNF662; ZNF677; ZNF711 |
| Ssc-miR-126 | ADAM9; AKAP13; BMP1; C18orf23; RNF165; CDKN2AIP; CRK; DIP2C; FBXO33; FRS2; GOLPH3; IRS1; ITGA6; PHF15; PLK2; SDC2; SOX21; SPRED1; TRAF7; ZNF219 |
| Ssc-miR-126* | A2ML1; ABCC9; ADAM22; ADAMTS3; ADAMTS6; AFF4; AKAP2; PALM2; ALS2CR4; AP1S3; APC; APPBP2; ARID1A; ARL11; ARSK; ASXL2; BCL11B; BRCTD1; ANKRD32; BTBD1; BTF3L4; BZW1; C10orf126; C12orf23; C12orf65; C14orf129; C18orf17; C1orf141; C3orf23; C4orf18; C8orf46; C8orf49; C8orf79; C9orf41; CA13; CALCB; CAMK2A; CASK; CCDC52; CCDC88A; CCNT2; CCPG1; CD2AP; CDC14B; CDC14C; CDC2L6; CDH7; CHL1; CHM; CHODL; CLCN5; CLEC12A; CLEC12B; CLLU1; CNGA4; CNOT4; COMMD2; CPD; CRB1; CREB5; CRNKL1; CSNK1G3; CXXC4; DAPP1; DCLK3; DCX; DDX59; DHX33; DIRC2; DMXL1; DUXA; DYRK2; E2F6; E2F7; EDA2R; EDG3; EFHA2; EGFL11; EIF2AK2; EIF3J; ENOX2; ENPP5; ERBB2IP; ESRRG; ETV1; EYA4; F9; FAM105B; FAM129A; FAM26C; FAM46A; FAM76B; FAM8A1; FBN2; FGF7; FLRT2; FOXN3; FPGT; TNNI3K; FSD1L; G3BP1; GABRA4; GABRB2; GBP1; GGT6; GJA7; GNE; GNRHR2; GOLGA8E; GOLGA8F; GOLGA8G; GOSR1; GPR85; GRIK2; GRIN2A; HEATR5B; HECTD2; HELLS; HIF1A; HMG2L1; HMGB1; HOXA13; HOXB6; HOXC8; HSD11B1; HSPB8; IDS; IKZF2; IL23R; ITGB8; JPH1; KCTD3; KIAA1033; KIAA1468; KIAA1622; KL; KLF12; KLRA1; L2HGDH; LASS6; LDLRAD2; LMO7; LONRF2; LPHN3; LRRC8B; MANEA; MAP3K2; MAP9; MCART6; MED14; MEF2D; MFAP4; MIB1; MIER3; MIPOL1; MKLN1; MMAA; MMP16; MPHOSPH1; MPP7; MRPL42; MSH2; MSRB3; MYEF2; MYSM1; NAT1; NCAN; NCOA7; NDUFS1; NEBL; NFAT5; NFIB; NRK; NSL1; NT5DC1; NTRK2; NUDT12; NUP43; NWD1; ODF2L; OSBPL8; OTUD3; OTUD4; PAN3; PCAF; PCGF5; PDE1C; PDE4D; PDE7B; PDGFD; PDGFRA; PELI2; PHYHIPL; PI15; PIP5K3; PLAC2; PLAG1; PLCB1; PLCXD3; PLEKHH2; PLEKHK1; PLSCR4; POFUT1; POU2F1; PPARGC1A; PPIAL4; PPIL1; PPP1R15B; PPP1R1C; PRRX1; PTEN; PTENP1; PTGFR; PTGS2; PTPRD; RAB11FIP2; RAB8B; RABL3; REPS2; RFX4; RFXDC2; RGR; RGS5; RNMT; RPE; RSBN1; SCAMP1; SCYE1; SGCB; SGTB; SH3BGRL2; SLC12A2; SLC16A9; SLC1A2; SLC25A24; SLC26A4; SLC2A13; SLC35B4; SLC38A2; SLC5A3; SLC7A2; SMAD4; SOCS6; SPAST; SPRY3; SRFBP1; SSX2IP; ST8SIA3; STEAP2; STYX; SYT10; TBC1D15; TCFL5; TFEC; TFPI; TGFBR1; TLL1; TLOC1; TMEM16E; TMEM182; TMEM26; TMEM47; TNFAIP8L3; TNRC6A; TRDN; TRIM33; TRIM8; TRPS1; TSC22D4; TTF2; TXNDC10; UBE2W; UBR1; UGT2A3; UNC5D; USP10; USP14; VAPA; VCPIP1; VIL1; VTA1; WDR35; WHSC1; XIRP2; YAP1; YES1; YOD1; YTHDC1; ZBTB41; ZBTB7B; ZBTB8; ZC3H13; ZC3H6; ZDHHC17; ZFP14; ZIC4; ZNF10; ZNF264; ZNF294; ZNF33A; ZNF33B; ZNF507; ZNF566; ZNF608; ZNF615; ZNF81; ZPLD1 |
| Ssc-miR-99a | ADCY1; BAZ2A; CDC25A; CYP26B1; EIF2C2; EPC2; FAM126B; FGFR3; FRAP1; FZD5; FZD8; GIYD1; GIYD2; HS3ST2; HS3ST3B1; ICMT; INSM1; JMJD3; KBTBD8; MBNL1; MTMR3; NTRK3; PHOX2B; PODXL; PPP3CA; RAVER2; RRAGD; SETD1B; ST5; SULT1A3; SULT1A4; TRIB2; VLDLR; ZZEF1 |
| Bta-miR-193b | ABI2; ADAMTS13; AFF4; ARMC1; BAK1; BCL2L7P1; C1orf21; C20orf117; CTGLF1; CTGLF2; CTGLF7; FLI1; FOSL2; IPO9; KIAA1797; MACF1; NT5DC3; OLFML2A; PADI2; PAG1; PHF15; PTPRT; RAB22A; RAD21L1; SNPH; SNX1; STX16; SV2B; TMEM164; TMEM63A; TXLNA; VASH1; ZBTB5; ZNF346; ZNF445 |
| Ssc-miR-486 | MACF1; NSL1; NTRK3; PFDN6; PTEN; PTENP1; ZNF208 |
| Ssc-let-7f | AATK; ABCB9; ABCC10; ABCC13; ABCC5; ABCC8; ABCD4; ABL2; ACP1; ACSL6; ACTR10; ACTR2; ACVR1B; ACVR1C; ACVR2A; ACVR2B; ADAM15; ADAMTS1; ADAMTS14; ADAMTS5; ADAMTS6; ADAMTS8; ADCY9; ADIPOR2; ADRB1; ADRB2; ADRB3; AFF2; AGBL2; AGPAT6; AHCTF1; AHCTF1P; AKAP6; AKT2; ALKBH1; ALPK3; AMMECR1L; AMOT; AMT; ANGPTL2; ANK3; ANKFY1; ANKRA2; ANKRD18A; ANKRD28; ANKRD46; ANKRD49; AP1S1; AP4B1; APBB3; APC2; APPBP2; ARG2; ARHGAP12; ARHGAP20; ARHGAP28; ARHGEF15; ARID3A; ARID3B; ARL4D; ARL5A; ARL6IP6; ARMC8; ARRDC4; ASAH3L; ASH1L; ATF7; ATG16L1; ATG9A; ATP2A2; ATP2B1; ATP2B3; ATP2B4; ATP2C1; ATP6V1F; ATP7B; ATPAF1; ATXN1; ATXN2; ATXN7L2; AURKB; AVEN; B3GAT1; B3GNT1; B3GNT7; BACH1; BAHD1; BAT3; BCAP29; BCAT1; BCL2L1; BCL7A; BDP1; BEGAIN; BIN3; BMP2; BNC2; BOLL; BPTF; BRD3; BSDC1; BSN; BTBD3; BTF3L4; BZW1; BZW2; C10orf56; C10orf6; C10orf64; WDFY4; C11orf57; C14orf28; C14orf32; C15orf29; C15orf39; C15orf41; C16orf63; C18orf21; C18orf23; RNF165; C19orf39; C19orf47; C1orf21; C1orf26; C20orf54; C21orf29; C22orf30; C22orf9; C3orf52; C3orf57; C3orf63; C3orf64; C6orf107; C6orf120; C6orf167; C6orf168; C6orf211; C8orf44; C8orf58; C9orf100; C9orf41; C9orf7; CABLES2; CACNA1E; CACNA1I; CACNG4; CALD1; CALML4; CALU; CAP1; CAPN3; CASKIN1; CASP3; CBFA2T3; CBL; CBX2; CCDC100; CCDC113; CCDC4; CCDC76; CCL3; CCL3L1; CCL3L3; CCL7; CCND1; CCND2; CCNF; CCNJ; CCNJL; CCNY; CCR7; CD164; CD200; CD200R1; CD276; CD59; CD86; CDC25A; CDC34; CDC42SE1; CDCA8; CDH1; CDK6; CDKN1A; CDV3; CDYL; CECR6; CEECAM1; CEP110; CEP135; CFL2; CGNL1; CHD4; CHD7; CHD9; CHRD; CHRDL2; CHST3; CLASP2; CLCN5; CLDN12; CLP1; CMTM6; CNOT2; CNOT6L; COIL; COL11A1; COL14A1; COL15A1; COL19A1; COL1A1; COL1A2; COL24A1; COL27A1; COL3A1; COL4A1; COL4A2; COL4A3BP; COL4A5; COL4A6; COL5A2; COL9A1; COL9A3; CPA4; CPEB1; CPEB2; CPEB3; CPEB4; CPM; CPSF4; CRB1; CRB2; CRCT1; CREB3L2; CREM; CRTAP; CRY2; CTDSPL2; CTNS; CYB561D1; CYP19A1; CYP2E1; CYP46A1; DAB1; DAGLA; DAPK1; DARS2; DCLRE1B; DCUN1D2; DCUN1D3; DCX; DDEF1; DDI2; DDN; DDO; DDTL; DDX19A; DDX19B; DDX26B; DGAT2L4; DIABLO; DIAPH2; DICER1; DKK3; DLC1; DLGAP1; DLGAP4; DLST; DLSTP; DMD; DMP1; DNA2L; DNAH2; DNAJC11; DNMBP; DOCK3; DPF2; DPH1; DPYSL3; DST; DTX2; DTX4; DUSP1; DUSP16; DUSP4; DUSP9; DVL3; DYRK1A; DZIP1; E2F2; E2F5; E2F6; EDA; EDAR; EDEM3; EDN1; EEA1; EEF2K; EFHD2; EGLN2; EGR3; EIF2C4; EIF2S2; EIF4G2; ELF4; ELOVL4; ENOX2; ENPP3; EPB41; EPHA3; EPHA4; EPHB1; ERGIC2; ERO1L; ETNK1; ETNK2; EXOC3L; EZH1; FAM103A1; FAM104A; FAM105B; FAM116A; FAM118A; FAM125B; FAM135A; FAM135B; FAM43A; FAM70A; FAM83G; FAM96A; FARP1; FASLG; FASTK; FBN1; FBXL12; FBXL19; FBXO24; FBXO30; FCHSD1; FGF11; FGF5; FIGN; FLNA; FNDC3A; FNDC3B; FOXN3; FOXP1; FOXP2; FRAS1; FREM2; FRMD4A; FRMD4B; FRS2; FSD1L; FZD4; G3BP1; GAB2; GABBR2; GABPA; GALC; GALE; GALNT1; GALNT2; GALNTL2; GAN; GAS7; GATM; GCNT4; GDAP2; GDF6; GDPD1; GEMIN7; GFOD1; GGA3; GHR; GIPC1; GIYD1; GIYD2; GJA7; GLMN; GLRX; GLT8D3; GNAL; GNAT1; GNG5; GNPTAB; GNS; GOLGA4; GOLGA7; GOLT1B; GOPC; GPATCH3; GPR137; GPR137C; GPR162; LEPREL2; GPR26; GRAMD3; GRB10; GRIK2; GRIN3A; GRPEL2; GSG1L; GTF2I; GYG2; HABP4; HAND1; HAND2; HAS2; HDLBP; HDX; HECTD2; HFM1; HIC2; HIF3A; HK2; HLF; HMGA1; HOOK1; HOXA1; HOXA9; HOXB4; HOXC11; HOXD1; HS2ST1; HSD17B11; HSPA14; HTR1E; HTR4; ICK; ICMT; ICOS; IDE; IDH2; IGF1; IGF1R; IGF2BP1; IGF2BP2; IGF2BP3; IGSF1; IKBKAP; IKBKE; IKZF2; IL10; IL13; IL28RA; IL6; IL8; INDOL1; INPP5A; INSR; INTS2; IPO9; IQCB1; IRS2; ISG20L1; ITGA4; ITGB3; ITGB8; ITSN1; JMJD1A; KATNAL1; KCNC4; KCNE3; KCTD10; KCTD17; KCTD21; KIAA0040; KIAA0258; KIAA0319L; KIAA0329; KIAA0406; KIAA0427; KIAA0515; KIAA0776; KIAA1033; KIAA1147; KIAA1274; KIAA1467; KIAA1539; KIAA2022; KIF21B; KIF2A; KLF9; KLHDC8B; KLHL13; KLHL23; KLHL31; KLHL6; KPNA1; KPNA4; KPNA5; KREMEN1; KTELC1; L2HGDH; LASS2; LBH; LBR; LCOR; LEPROTL1; LGR4; LIMD1; LIMD2; LIMK2; LIN28; LIN28B; LINGO1; LIPH; LMX1A; LONRF3; LOR; LOXL3; LOXL4; LPGAT1; LRIG2; LRIG3; LRRC17; LRRC20; LRRC59; LRRC8B; LRRFIP1; LSM11; LYPLA3; LYVE1; MACF1; MAN2A2; MAP3K1; MAP3K3; MAP3K7IP2; MAP4K3; MAP4K4; MAPK4; MAPK6; MAPK8; MARCH9; MARS2; MDFI; MECP2; MED28; MED6; MEF2D; MEGF11; MEIS2; MEIS3; MESDC1; MEST; MGA; MGAT3; MGAT4A; MGLL; MIB1; MLL5; MLLT10; MLXIP; MMP11; MNT; MOBKL3; MON2; MRM1; MRS2L; MSN; MTPN; MUSTN1; TMEM110; MUTED; TXNDC5; MXD1; MYB; MYCBP; MYCL1; MYCN; MYO1F; MYO5B; MYRIP; NAB1; NAGA; NAP1L1; NARG1; NARG1L; NAT12; NCOA1; NCOA3; NCOA5; NCOA6; NCOR1; NDST2; NDST3; NEFM; NEK3; NEK9; NGFB; NIPA1; NKD1; NKIRAS2; NLK; NLN; NME4; NME6; NNT; NOVA1; NPAL1; NPEPL1; NR4A1; NR6A1; NRAS; NRK; NSMCE2; NT5C2; NUMBL; NXT2; OLFM4; ONECUT2; OPA3; OPRM1; OSBPL3; OSMR; OSTF1; OTOF; OTUD3; OTUD4; P4HA2; PAG1; PAK1; PANX2; PAPPA; PARD6B; PAX3; PBX2; PBX3; PCDH19; PCDH20; PCGF3; PCYT1B; PDE1C; PDGFB; PDSS1; PEX11B; PGM2L1; PGRMC1; PHACTR4; PHF8; PI4K2B; PIAS4; PIGA; PIK3IP1; PKIA; PKN2; PLA2G3; PLAGL2; PLCB2; PLCXD3; PLD3; PLD5; PLDN; PLEKHA6; PLEKHG6; PLEKHO1; PLXND1; PNKD; POGZ; POLL; POLR2D; POLR3D; POMT1; POU2F1; POU2F2; PPAPDC2; PPARA; PPARGC1A; PPARGC1B; PPP1R12B; PPP1R15B; PPP1R16B; PPP3CA; PPTC7; PQLC2; PRKAB2; PRMT8; PRPF38B; PRPF39; PRRX1; PRSS22; PRTG; PSCD3; PSD3; PTCH1; PTPN7; PTPRD; PTPRO; PTPRU; PURB; PXDN; PXMP4; PXT1; PYGO2; PYY2; RAB11FIP2; RAB11FIP4; RAB15; RAB22A; RAB3GAP2; RAB40C; RAB8B; RAG1; RAI16; RALB; RALGPS1; RANBP2; RAPGEF6; FNIP1; RASGRP1; RASL10B; RAVER2; RB1; RBM38; RBM9; RBPJ; RCN1; RCSD1; RDH10; RDX; REEP1; RFXDC1; RGAG1; RGS16; RIOK3; RNF20; RNF216L; RNF38; RNF44; RNF7; RNMT; RORC; RPS6KA3; RRAGD; RRM2; RRP1B; RSPO2; RSPRY1; RTKN; RUFY3; SAMD10; SBK1; SCD; SCN4B; SCN5A; SCRT2; SCUBE3; SCYL3; SDK1; SEC14L1; SEC14L5; SEC16B; SEC24C; SEC31B; SEMA3F; SEMA4C; SEMA4F; SEMA4G; SENP2; SENP5; SERPINB9; SETDB1; SFRS12; SFT2D3; SGCD; SH2B3; SIPA1L2; SLC10A2; SLC10A7; SLC12A9; SLC16A10; SLC16A9; SLC17A3; SLC1A4; SLC20A1; SLC25A18; SLC25A24; SLC25A27; SLC25A32; SLC25A4; SLC2A12; SLC30A4; SLC30A7; SLC31A1; SLC31A2; SLC35B1; SLC35D2; SLC37A4; SLC45A4; SLC4A4; SLC4A7; SLC5A6; SLC5A9; SLC6A1; SLC7A6; SLC8A2; SLC9A9; SLCO5A1; SMAD2; SMAP1; SMAP1L; SMARCAD1; SMARCC1; SMC1A; SMCR7; SMUG1; SNAP23; SNX11; SNX16; SNX30; SNX6; SOCS1; SOCS4; SOST; SP8; SPATA2; SPEG; SPIRE1; SPOCD1; SPRYD4; SPTBN4; SREBF2; SRGAP3; SSH1; ST7L; STAB2; STARD13; STARD3NL; STEAP3; STK24; STK40; STRBP; STX3; STXBP5; STYX; SUB1; SUCLG2; SUHW2; SULF1; SULF2; SULT1A3; SULT1A4; SURF4; SYT1; SYT11; SYT14; SYT2; SYT7; TACC3; TADA2L; TAF5; TAF9B; TARBP2; TAT; TBKBP1; TBX5; TEAD3; TEX261; TGDS; TGFBR1; TGFBR3; THBS1; THOC2; THRA; THRSP; TIMM17B; TMC7; TMED5; TMEM135; TMEM143; TMEM2; TMEM65; TMPRSS11F; TMTC3; TNFAIP3; TNFRSF1B; TNFSF9; TOB2; TOR1AIP2; TP53; TPP1; TRABD; TRAK2; TRAM2; TRHDE; TRIB1; TRIB2; TRIM33; TRIM41; TRIM67; TRIM71; TRPM6; TSC1; TSC22D2; TSPAN18; TSPAN2; TTC31; TTC9C; TTL; TTLL4; TTLL6; TUSC2; TXLNA; TXNDC13; UBE1; UBE2G2; UBN1; UBXD2; UFM1; UGCGL1; UHRF1; UHRF2; ULK2; UNC5A; USP12; USP21; USP24; USP32; USP38; USP44; USP47; USP49; USP6; UTRN; VANGL2; VASH2; VAV3; VCPIP1; VGLL3; VPS25; VSNL1; WAPAL; WARS2; WASL; WDFY3; WDR26; WDR37; WDR42A; WIPI2; WNT1; XK; XKR8; XRN1; XYLT1; XYLT2; YAF2; YAP1; YIPF1; YOD1; YPEL2; YTHDF3; ZBTB10; ZBTB39; ZBTB5; ZC3H3; ZCCHC3; ZFYVE16; ZFYVE26; ZMAT1; ZNF10; ZNF197; ZNF200; ZNF248; ZNF275; ZNF282; ZNF318; ZNF341; ZNF343; ZNF354A; ZNF354B; ZNF362; ZNF462; ZNF473; ZNF512; ZNF512B; ZNF518; ZNF566; ZNF583; ZNF641; ZNF644; ZNF689; ZNF710; ZNF740; ZNF784; ZNF81; ZPLD1; ZRF1; ZSWIM4; ZSWIM5 |
|  |  |

Bta: *Bos taurus*, Hsa: *Homo sapiens*, Ssc: *Sus scrofa*.
Potential mRNA target genes for differentially expressed miRNAs predicted *in silico* with DIANA - microT v3.0 web server.
